# Enantioselective Catalytic [4+1]‐Cyclization of *ortho*‐Hydroxy‐*para*‐Quinone Methides with Allenoates

**DOI:** 10.1002/chem.201901784

**Published:** 2019-05-21

**Authors:** Katharina Zielke, Ondřej Kováč, Michael Winter, Jiří Pospíšil, Mario Waser

**Affiliations:** ^1^ Institute of Organic Chemistry Johannes Kepler University Linz Altenbergerstraße 69 4040 Linz Austria; ^2^ Department of Organic Chemistry Faculty of Science Palacky University tř. 17. listopadu 1192/12 771 46 Olomouc Czech Republic; ^3^ Laboratory of Growth Regulators The Czech Academy of Sciences, Institute of Experimental Botany & Palacký University Šlechtitelů 27 78371 Olomouc Czech Republic

**Keywords:** allenoates, annulation, diastereoselectivity, enantioselectivity, organocatalysis

## Abstract

The first highly asymmetric catalytic synthesis of densely functionalized dihydrobenzofurans is reported, which starts from *ortho*‐hydroxy‐containing *para*‐quinone methides. The reaction relies on an unprecedented formal [4+1]‐annulation of these quinone methides with allenoates in the presence of a commercially available chiral phosphine catalyst. The chiral dihydrobenzofurans were obtained as single diastereomers in yields up to 90 % and with enantiomeric ratios up to 95:5.

## Introduction

**Scheme 1:**
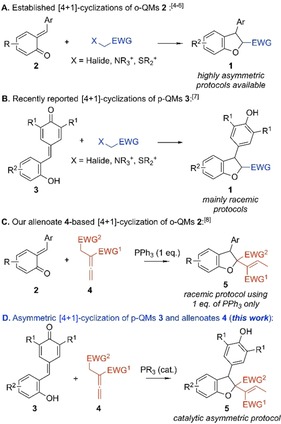
Previous [4+1]‐approaches for the syntheses of dihydrobenzofurans (A–C) and the herein reported novel catalytic asymmetric strategy (D).

The 2,3‐dihydrobenzofuran scaffold is a prominent structural motif found in numerous biologically active (natural) compounds^1^ and the development of novel synthesis strategies to access these targets has been a heavily investigated topic over the last years.^2^, ^3^, ^4^, ^5^, ^6^, ^7^, ^8^ One especially appealing approach to access chiral 2,3‐dihydrobenzofurans is the formal [4+1]‐cyclization^9^ between a suitable C1 building block and a carefully chosen (maybe in situ generated) acceptor–donor containing C4 building block. The most versatile class of C4 building blocks used to obtain the dihydrobenzofuran skeleton **1** via a formal [4+1]‐cyclization are *ortho*‐quinone methides (*o*‐QMs) **2**.^10^ These, usually in situ generated, reactive compounds have recently been very successfully used for racemic as well as highly stereoselective [4+1]‐annulations with either sulfonium or ammonium ylides,^4^ α‐halocarbonyl compounds,^5^ or with diazocompounds as C1 synthons (Scheme 1 A).^6^ Alternatively, the hydroxy‐containing *para*‐quinone methides **3** have very recently emerged as powerful building blocks for formal (4+n)‐annulations as well.^7^, ^11^, ^12^, ^13^ Interestingly however, their applicability for asymmetric [4+1]‐cyclizations to access dihydrobenzofurans **1** has so far been rather limited, with highly asymmetric protocols still being rare (Scheme 1 B).^7^ Two years ago we reported the first highly enantioselective synthesis of compounds **1** by reacting preformed chiral ammonium ylides with in situ formed *o*‐QMs **2**.4c More recently we found that the highly functionalized allenoates **4** can undergo a very unique (and up to then unprecedented) formal [4+1]‐cyclization with acceptors **2** in the presence of a stoichiometric amount of PPh_3_ (Scheme 1 C).^8^ The (unexpected) outcome of this reaction was in sharp contrast to other previously described reactions between *o*‐QMs **2** and (differently substituted) allenoates, which all resulted in formal [4+2]‐annulations.^14^ Unfortunately however, we were only able to carry out this reaction in racemic manner, as even the use of a stoichiometric amount of different commonly used chiral phosphine catalysts gave low yields and poor enantioselectivities only.^8^ In especially we found that the in situ formed *o*‐QMs **2** decomposed rather rapidly under the previously developed reaction conditions, thus making a catalytic approach difficult. Given these limitations in catalyst turnover and asymmetric induction, we thought that maybe an alternative and slightly more stable acceptor would be beneficial to address these challenges. Thus, we decided to investigate if this methodology may also be extended to the formal [4+1]‐cyclization of the *o*‐hydroxy‐containing *p*‐QMs **3** with allenoates **4**.^12^ We reasoned that this preformed and, compared to **2**, more stable acceptor molecule may be better suited to establish a truly catalytic as well as asymmetric protocol, which would then allow for the first enantioselective [4+1]‐annulation of *p*‐QMs **3** to access the highly functionalized chiral dihydrobenzofurans **5** (Scheme 1 D).

## Results and Discussion

### Initial optimization of the racemic reaction

**Scheme 2:**
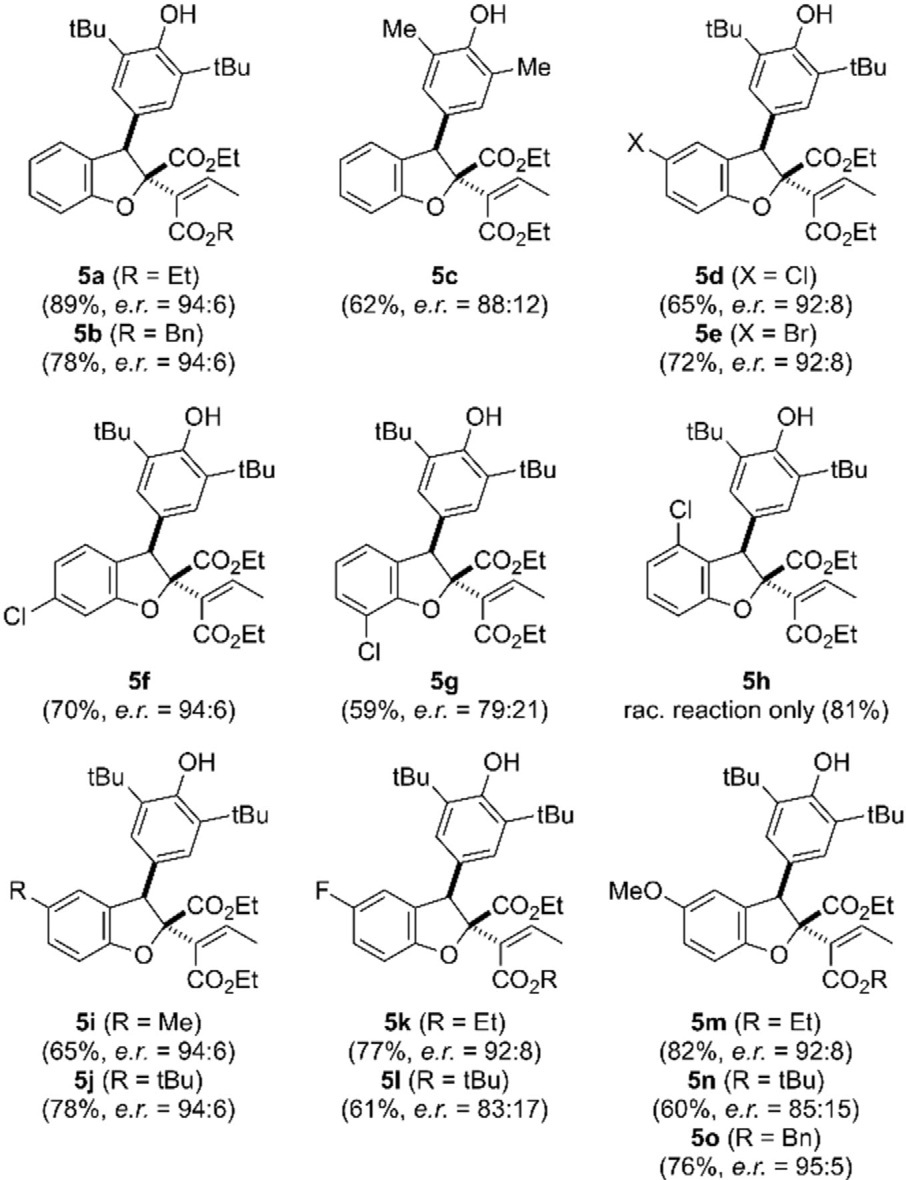
Application scope (Conditions: 0.05–0.2 mmol **3**, 2 equiv **4**, 2 equiv K_2_CO_3_, 20 mol % **B**, toluene (0.025 m), 10 °C, min. 20 h; All yields are isolated yields and enantiomeric ratios were determined by HPLC using a chiral stationary phase with the (+)‐enantiomer being the major product in each case).

We started our investigations by carrying out the racemic reaction between *p*‐QM **3 a** and diethyl allenoate **4 a** in the presence of PPh_3_ (Table 1 gives an overview of the most significant screening results). Our first reactions were carried out in analogy to the conditions developed for the annulation of *o*‐QMs **2** 8 (please note that we previously used a twofold excess of the quinone methide **2** to compensate for its competing decomposition under the reaction conditions). Gratifyingly the targeted dihydrobenzofuran **5 a** could be obtained as a single diastereomer in this initial attempt already (entry 1). The relative configuration of the product **5 a** was confirmed by NOESY experiments (as shown in Scheme 4) and we also observed the same correlations for other products **5** later (Scheme 2). As the reaction was found to be rather slow, with significant amounts of unreacted **3 a** being recovered (indicating its increased stability compared to *o*‐QMs **2**), we next increased the amount of base (entry 2), which however had a detrimental effect (complete decomposition of starting materials).

**Table 1:** Initial screening with PPh_3_.

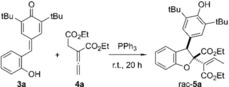					
Entry^[a]^	**3 a**:**4 a**	PPh_3_ [equiv.]	Base ([eq.])	Solvent	Yield [%]^[b]^
1	2:1	1	Cs_2_CO_3_ (2)	CH_2_Cl_2_	16^[c]^
2	2:1	1	Cs_2_CO_3_ (10)	CH_2_Cl_2_	–
3	1:2	1	Cs_2_CO_3_ (2)	CH_2_Cl_2_	33^[c]^
4	1:2	1	Cs_2_CO_3_ (2)	toluene	45^[d]^
5	1:2	1	Cs_2_CO_3_ (2)	EtOAc	–
6	1:2	1	Cs_2_CO_3_ (0.2)	CH_2_Cl_2_	63
7	1:2	1	K_2_CO_3_ (2)	CH_2_Cl_2_	82
8	1:2	0.2	K_2_CO_3_ (2)	CH_2_Cl_2_	81
9	1:2	0.1	K_2_CO_3_ (2)	CH_2_Cl_2_	49
10	1:2	0.2	K_2_CO_3_ (0.5)	CH_2_Cl_2_	89
11	1:2	0.2.	–	CH_2_Cl_2_	90 (86)^[e]^

[a] All reactions were carried out on 0.1 mmol scale (based on the *p*‐QM **3 a**), [b] Isolated yield; [c] Incomplete conversion of **3 a** and noticeable amounts of side products; [d] complete conversion of **3 a** and large amounts of unidentified side products; [e] 1 mmol scale reaction.

As decomposition of the acceptor **3 a** was not very fast in the first attempts with 2 equivalents of base, we next used an excess of allenoate **4 a**, which led to a measurable increase in yield (entry 3). The screening of different solvents revealed that toluene allows for a slightly higher yield (entry 4), but also accompanied with a more pronounced formation of various not identified side‐ or decomposition products. Other solvents did not give satisfactory results (see entry 5 for one example) and so further optimizations with CH_2_Cl_2_ were carried out. Very interestingly, lowering the amount of base (entry 6) significantly improved the yield and suppressed side product formation. By testing other bases, K_2_CO_3_ turned out to be the most promising (entry 7). It should be noted that other simple trialkylphosphines were tested as well,^15^ but in analogy to our previous observation^8^ these did not allow for this [4+1]‐annulation.

With these first high yielding conditions set, we next lowered the amount of PPh_3_. Gratifyingly, and in sharp contrast to the reaction with *o*‐QMs **2**,^8^ the use of 20 mol % PPh_3_ allowed for the same yield as when using a stoichiometric amount (compare entries 7 and 8). Further lowering of the catalyst amount unfortunately slowed down the reaction measurably (entry 9). Considering the beneficial effect of using less base when using Cs_2_CO_3_ (entry 6), we finally also lowered the amount of K_2_CO_3_ (entries 10, 11), and much to our surprise the reaction proceeded well even without any base (entry 11; the reaction was reproduced several times on different scales and also on 1 mmol scale).

### Development of an asymmetric catalytic protocol

Having established high yielding and robust catalytic procedures for the racemic synthesis of **5 a** we next focused on the use of chiral phosphine catalysts. As already mentioned before, we were not able to identify a suited asymmetric catalyst for our previous [4+1]‐annulation of *o*‐QMs **2**. However, given the fact that *p*‐QM **3 a** performed very well in the racemic reaction and also allowed for a catalytic approach, we were confident that the well‐described bulky chiral phosphines **A** 16 or **B** 17 may allow for a truly catalytic enantioselective protocol (Table 2). We first used the binaphthyl‐based phosphines **A1**–**3**, but unfortunately neither of them allowed for any product formation (entries 1–3). Gratifyingly however, by switching to the commercially available chiral spiro phosphine **B** ((*R*)‐SITCP)^17^ we observed a very clean and reasonably enantioselective product formation when using 20 mol % of this catalyst under base‐free conditions in CH_2_Cl_2_ (entry 4). Lowering the reaction temperature to 0 °C unfortunately did not allow for product formation anymore (entry 5). When carrying out the reaction in the presence of two equivalents of K_2_CO_3_, the outcome was only slightly affected in this solvent (entry 6).

**Table 2:** Development of an enantioselective catalytic protocol.

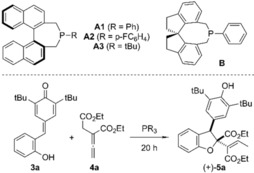						
Entry^[a]^	PR_3_ ([mol %])	Solvent	Base ([eq.])	Temp. [°C]	Yield [%]^[b]^	e.r.^[c]^
1	A1 (20)	CH_2_Cl_2_	–	25	–	–
2	A2 (20)	CH_2_Cl_2_	–	25	–	–
3	A3 (20)	CH_2_Cl_2_	–	25	–	–
4	B (20)	CH_2_Cl_2_	–	25	84	88:12
5	B (20)	CH_2_Cl_2_	–	0	–	–
6	B (20)	CH_2_Cl_2_	K_2_CO_3_ (2)	25	70	87:13
7	B (20)	toluene	–	25	87	94:6
8	B (20)	toluene	K_2_CO_3_ (2)	25	90	92:8
9	B (20)	toluene	K_2_CO_3_ (2)	10	89	94:6
10	B (20)	toluene	K_2_CO_3_ (2)	0	–	–
11	B (10)	toluene	K_2_CO_3_ (2)	10	–	–

[a] All reactions were carried out on 0.1 mmol scale (based on **3 a**). [b] Isolated yield. [c] Determined by HPLC using a chiral stationary phase with the (+)‐enantiomer as the major product. The relative configuration was assigned by NOESY experiments (see Scheme 4) but the absolute configuration could not be determined yet.

Interestingly, when changing to toluene (other solvents like THF were found to be not suited), we were able to improve the enantioselectivity significantly (entries 7–9). At room temperature reactions in the presence of K_2_CO_3_ as well as under base‐free conditions performed very similarly, with a slightly higher e.r. in the absence of base (compare entries 7 and 8). However, when we further investigated the application scope, we realized that the base‐mediated conditions were more robust when using differently substituted starting materials **3**, while not all of those allowed for good conversions under base‐free conditions. Other bases were found to be less satisfactory (with for example, Cs_2_CO_3_ giving lower yields and K_3_PO_4_ giving no product at all). We thus tested if any further improvement in the presence of 2 equivalents of K_2_CO_3_ may be possible (entries 8–11). However, lowering of the reaction temperature was possible to some extent only (entries 9, 10), but reducing the catalyst loading to 10 mol % was unfortunately not possible anymore (entry 11). Accordingly, the best‐suited and most robust catalytic enantioselective approach to access **5 a** as a single diastereomer was to carry out the reaction in toluene at 10 °C in the presence of 2 equivalents of K_2_CO_3_ by using 20 mol % of the commercially available phosphine catalyst **B** (entry 9, the reaction was reproduced by different persons on 0.05–0.1 mmol **3 a** scale giving identical results).

### Application scope

Having established a high yielding and robust catalytic procedure for the synthesis of dihydrobenzofuran **5 a**, we next tested the use of differently substituted quinone methides **3** and allenoates **4** (Scheme 2).

First, we could show that replacement of one of the allenoate ethyl ester groups for a benzyl ester was tolerated very well (see product **5 b**). Then it turned out that a dimethyl‐based *p*‐QM **3** can be used as well to obtain the enantioenriched product **5 c** (albeit with a slightly lower selectivity than for the parent *t*Bu‐based **5 a**). Interestingly, substituents in the 5 and 6‐position of the benzofuran backbone were very well tolerated (see compounds **5 d**–**f**, **5 i**–**k**, **5 m**). In contrast, substituents in positions 4 and 8 turned out to be more limiting and product **5 g** was only accessible with a rather low enantiomeric ratio of 79:21. Surprisingly, compound **5 h** was not formed at all under the asymmetric conditions (even with longer reaction times). We were however able to obtain racemic **5 h** in high yield when using PPh_3_ as an achiral catalyst. Very interestingly, while we found initially that benzyl ester containing allenoates were tolerated similarly well as ethyl ester‐based ones (see targets **5 a** and **5 b**), we found that *tert*‐butyl esters resulted in somewhat lower enantiomeric ratios compared to ethyl and benzyl esters (compare **5 k** and **5 l** as well as **5 m**, **5 n**, and **5 o**). All asymmetric reactions were initially carried out on 0.05 mmol scale of the limiting agent **3** and we also reproduced selected reactions on up to 0.2 mmol scale without affecting the outcome, thus indicating that the asymmetric procedure is of similar robustness as the racemic one (Table 1, entry 11).

All substrate combinations gave the (+)‐enantiomer as the major product, but unfortunately, it has not been possible to obtain suitable crystals of any of the products **5** to determine the absolute configuration by single‐crystal X‐ray analysis.

**Scheme 3:**
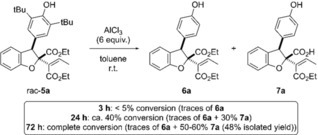
AlCl_3_‐mediated transformations of *rac*‐**5 a**.

It has been described by others that the *tert*‐butyl groups of the phenol derivatives obtained by addition of nucleophiles to QMs can be cleaved off under (Lewis) acidic conditions.7a, 11c We thus carried out a few (unoptimized) test reactions to see if a similar debutylation is also possible on the highly functionalized diester‐containing dihydrobenzofuranes **5** (Scheme 3). Carrying out the reaction at elevated temperature only led to decomposition. In contrast, at room temperature, the slow formation of the debutylated diester **6 a** was observed by MS. Interestingly however, the major product was found to be the debutylated monoester **7 a** that was formed in around 30 % after one day and around 50–60 % after 3 days (accompanied with some decomposition products) and which could also be isolated after column chromatography (NMR clearly confirmed that the ester group on the stereogenic center was hydrolyzed). It should be noted that no further attempts to optimize this reaction were undertaken, but this result clearly shows that the highly functionalized compounds **5 a** can be used for further transformations and that the two ester groups have different reactivities.

### Mechanistic considerations

**Scheme 4:**
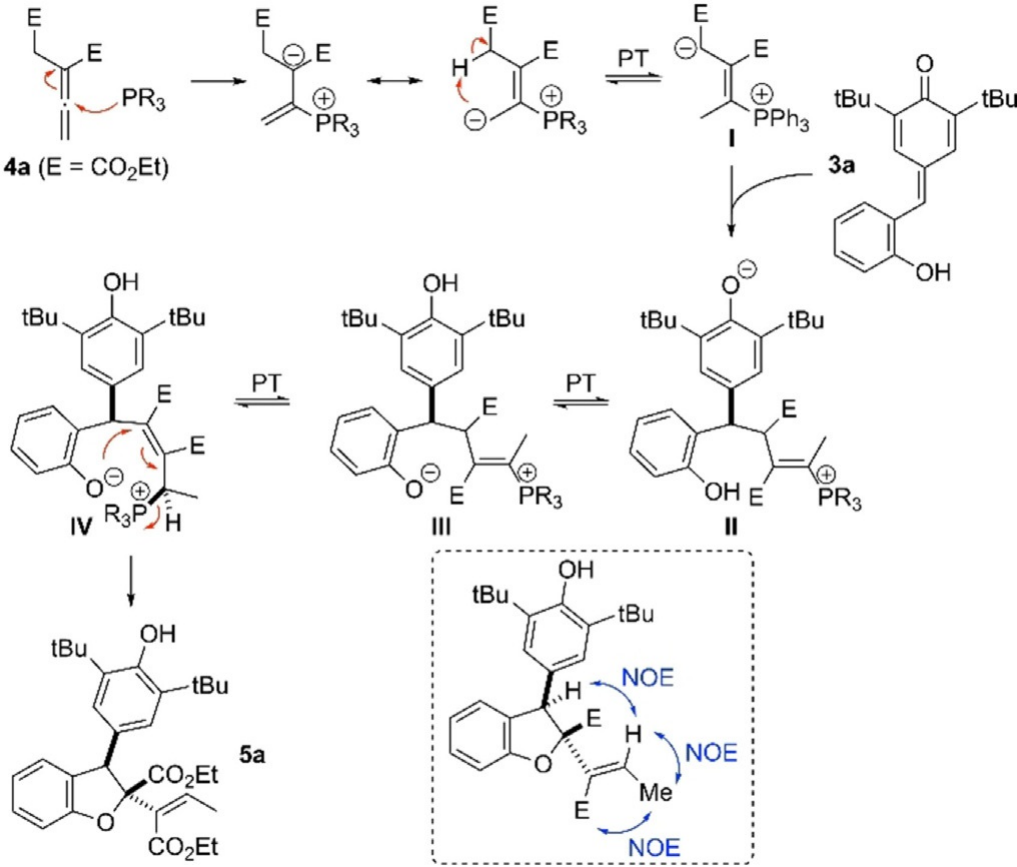
Postulated mechanism and NOESY results to determine the relative configuration.

Mechanistically this is a rather complex reaction and it should be admitted that so far, we only have some hints that may allow us to postulate the mechanistic scenario depicted in Scheme 4. This proposal is also based on our recent observations made for the racemic [4+1]‐annulation of *o*‐QMs **2** where we found that intramolecular rapid proton transfers are crucial to explain the outcome of this [4+1]‐cyclization.^8^

Addition of the phosphine to the allenoate is supposed to give the required zwitterion **I** after proton transfer on the primary addition product. Following the reaction between PPh_3_ and **4 a** by ^31^P NMR shows the appearance of two new signals around 27 ppm (the parent PPh_3_ peak is at −5 ppm) substantiating the formation of alkylated phosphine species (these addition products decomposed very quickly in the absence of any electrophile). Upon addition of the quinone methide **3 a** immediately a strong red color evolves, which can be rationalized by the 1,6‐addition of **I** to **3 a** to give the phenolate **II**. Similar color changes can also be observed when adding different nucleophiles to other *p*‐QMs, substantiating the assumed initial 1,6‐addition. With respect to the nature of the electrophile **3 a** one could however also postulate that a prototropic shift from the phenol to the *para*‐QM moiety gives an *ortho*‐QM in situ, which then reacts with **I** to give **III** directly.^18^ However, we found compound **3 a** being rather stable under basic conditions and we never observed any other species or got any experimental hint that supports this pathway, but it should not be ruled out completely with the current state of knowledge. The phenolate **II** then needs to undergo two proton transfer reactions towards the betaine **IV**, which can then finally react to the product **5 a** via an Sn2′‐type cyclization. We have recently shown for the cyclizations of *o*‐QMs **2** that these proton transfers are rather likely processes and we reason that the presence of a base is beneficial for these reactions, which would be an explanation why the herein presented [4+1]‐cyclization is more robust under basic conditions. This beneficial effect of base became especially pronounced in those cases where no electron‐donating ring substituent *para* to the OH group is present (these reactions usually proceeded a bit slower as well). This observation supports a scenario where the final ring closure may be the rate‐determining step, which also rationalizes why slightly larger amounts of catalyst were necessary to obtain satisfying catalyst turnover.

With respect to the observed stereoselectivity it is likely that the catalyst controls the absolute configuration in the 1,6‐addition step. An alternative may be a less selective 1,6‐addition followed by base‐mediated isomerization of the benzylic position on one of the chiral catalyst‐bound intermediates **II** or **III**. However, as the observed enantioselectivity was more or less the same under basic and base‐free conditions (compare with Table 2), this option seems less likely. The diastereoselectivity is then controlled in the final proton transfer–cyclization sequence. Given the fact that S_N_2′ reactions usually proceed with a *cis*‐orientation of nucleophile and leaving group^19^ the proton transfer towards **IV** is supposed to be highly selective, and may be steered by electrostatic attraction between the phenolate anion and the phosphonium cation in the nonpolar reaction solvent. However, it should clearly be pointed out that this is just a mechanistic hypothesis and although we were able to observe the presence of some alkylated phosphonium species by ^31^P NMR during the reaction, none of these intermediates could be isolated or more carefully analyzed.

## Conclusions

The first highly asymmetric catalytic formal [4+1]‐annulation of *o*‐hydroxy‐*p*‐quinone methides **3** with allenoates **4** has been developed. The outcome of this reaction is in sharp contrast to other recently reported reactions between quinone methides **3** and allenoates.^12^ Key to success was the use of the commercially available chiral phosphine **B** as a catalyst under carefully optimized reaction conditions. This methodology allowed for the so far unprecedented synthesis of the chiral dihydrobenzofurans **5** as single diastereomers in yields up to 90 % and with enantiomeric ratios up to 95:5.

## Experimental Section

General details can be found in the online Supporting Information. This document also contains detailed synthesis procedures and analytical data of novel compounds and reaction products as well as copies of NMR spectra and HPLC traces.

### General asymmetric [4+1]‐cyclization procedure

A mixture of the *para*‐quinone methide **3** (0.05–0.2 mmol), K_2_CO_3_ (2 equiv), and chiral phosphine **B** (20 mol %) was cooled to 10 °C and a solution of the allenoate **4** (2 equiv) in dry toluene (20 mL per mmol **4**) was added. The resulting mixture was stirred at 10 °C under an Ar atmosphere for approximately 20 h. The mixture was diluted by adding CH_2_Cl_2_ (5 mL), filtrated over a pad of Na_2_SO_4_ and the residue was rinsed with CH_2_Cl_2_ (5×5 mL). The combined organic layers were evaporated to dryness (under reduce pressure) and the products were purified by silica gel column chromatography (gradient of heptanes and EtOAc) giving the corresponding dihydrobenzofurans **5** in the reported yields and enantiopurities (Syntheses of racemic samples were carried out in analogy using PPh_3_ instead).

### Dihydrobenzofuran 5 a

Obtained as a yellow residue in 89 % and e.r.=94:6. ${\left[\alpha \right]}_{D}^{23}$ =64.6 (*c*=0.15, CHCl_3_, *e.r*.=94:6); 1H NMR (300 MHz, δ, CDCl_3_, 298 K): *δ*=0.82 (t, *J=*7.2 Hz, 3 H), 1.36 (t, *J=*7.1 Hz, 3 H), 1.36 (s, 18 H), 1.89 (d, *J=*7.2 Hz, 3 H), 3.52–3.77 (m, 2 H), 4.26–4.36 (m, 2 H), 5.11 (s, 1 H), 5.24 (s, 1 H), 6.40 (q, *J=*7.1 Hz, 1 H), 6.83 (s, 2 H), 6.94 (t, *J=*7.3 Hz, 1 H), 7.01–7.09 (m, 2 H), 7.19–7.26 ppm (m, 1 H). ^13^C NMR (75 MHz, δ, CDCl_3_, 298 K): *δ*=13.4, 14.2, 15.5, 30.2, 34.2, 55.8, 60.8, 61.1, 94.0, 110.2, 121.8, 125.7, 126.1, 128.9, 129.4, 130.0, 132.7, 133.2, 135.1, 153.0, 158.1, 167.2, 168.4 ppm; HRMS (ESI): *m*/*z* calcd for C_31_H_40_O_6_: 509.2898 [*M*+H]+; found: 509.2897. The enantioselectivity was determined by HPLC (YMC Chiral Art Cellulose‐SB, eluent: hexane/*i*PrOH=95:5, 0.5 mL min^−1^, 10 °C, retention times: *t*_major_=9.4 min, *t*_minor_=11.0 min).

## Conflict of interest

The authors declare no conflict of interest.

## Supporting information

As a service to our authors and readers, this journal provides supporting information supplied by the authors. Such materials are peer reviewed and may be re‐organized for online delivery, but are not copy‐edited or typeset. Technical support issues arising from supporting information (other than missing files) should be addressed to the authors.

Supplementary
