# Supplementary material for: Enantioselective Catalytic [4+1]‐Cyclization of ortho‐Hydroxy‐para‐Quinone Methides with Allenoates
Source: Chemistry. 2019 May 21;25(34):8163–8. doi: 10.1002/chem.201901784 (PMC6618147; doi:10.1002/chem.201901784)
Supplement: Supplementary file 1 — Supplementary [file CHEM-25-8163-s001.pdf]

CHEMISTRY
A European Journal

Supporting Information

Enantioselective Catalytic [4+1]-Cyclization of *ortho*-Hydroxy-*para*-Quinone Methides with Allenoates

Katharina Zielke+,[a] Ondřej Kováč+,[b] Michael Winter,[a] Jiří Pospíšil,[b, c] and Mario Waser*[a]

chem_201901784_sm_miscellaneous_information.pdf

SUPPORTING INFORMATION

Enantioselective Catalytic (4+1)-Cyclization of ortho-Hydroxy para-Quinone Methides with Allenoates

Katharina Zielke[a], Ondřej Kováč[b], Michael Winter[a], Jiří Pospíšil[b,c] and Mario Waser[a]*

[a] Institute of Organic Chemistry, Johannes Kepler University Linz, Altenbergerstraße 69, 4040 Linz, Austria

[b] Department of Organic Chemistry, Faculty of Science, Palacký University, tř. 17. listopadu 1192/12, CZ-771 46 Olomouc, Czech Republic

[c] Laboratory of Growth Regulators, The Czech Academy of Sciences, Institute of Experimental Botany & Palacký University, Šlechtitelů 27, CZ-783 71 Olomouc, Czech Republic

E-mail: Mario.waser@jku.at

1 General Information:

1.1 General Methods

^{1}H- and ^{13}C-NMR spectra were recorded on a Bruker Avance III 300 MHz spectrometer with a broad band observe probe and a sample changer for 16 samples, on a Bruker Avance DRX 500 MHz spectrometer, and on a Bruker Avance III 700 MHz spectrometer with an Ascend magnet and TCI cryoprobe, which are both property of the Austro-Czech NMR-Research Center "RERI-uasb". All NMR spectra were referenced on residual solvent peak $CHCl_3$ (δ= 7.26 ppm for ^{1}H and 77.2 for ^{13}C). High resolution mass spectra were obtained using an Agilent 6520 Q-TOF mass spectrometer with an ESI source and an Agilent G1607A coaxial sprayer or a Thermo Fisher Scientific LTQ Orbitrap XL with an Ion Max API Source. Analyses were made in the positive ionization mode if not otherwise stated. Purine (exact mass for $[M+H]^+$ = 121.050873) and 1,2,3,4,5,6-hexakis(2,2,3,3-tetrafluoropropoxy)-1,3,5,2,4,6-triazatriphosphinane (exact mass for $[M+H]^+$ = 922.009798) were used for internal mass calibration.

The ratio of enantiomers was determined by HPLC Thermo Scientific Dionex Ultimate 3000 system with diode array detector with a CHIRAL ART Amylose-SA (250 x 4.6 mm, 5 µm), CHIRAL ART Celulose-SB (250 x 4.6 mm, 5 µm) as a chiral stationary phase with mobile phase 2-propanol/*n*-hexane.

All chemicals were purchased from commercial suppliers and used without further purification unless otherwise stated. All reactions were carried out under Argon.

The allenoates **4** were prepared as described recently. [1]

[1] H. Guo, Q. Xu, O. Kwon, *J. Am. Chem. Soc.* **2009**, *131*, 6318; Li R., Leng P., Liu B., Wang X., Ge Z., Li R. *Tetrahedron*, **2016**, *72*, 5707-5712.

2 Syntheses

2.1 Preparation of para-Quinone Methides[2]

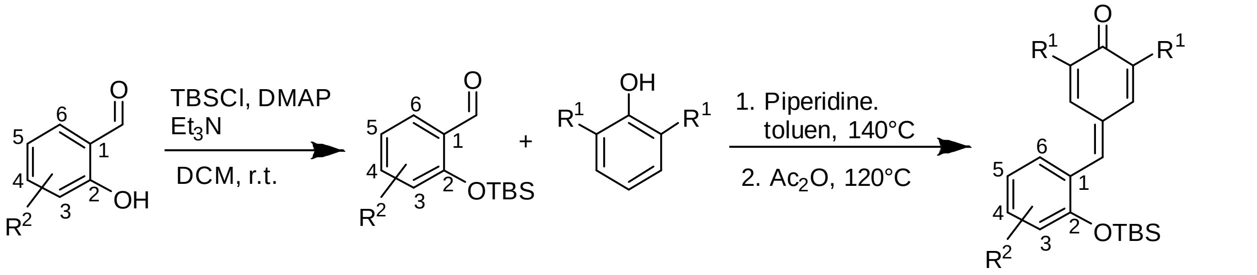

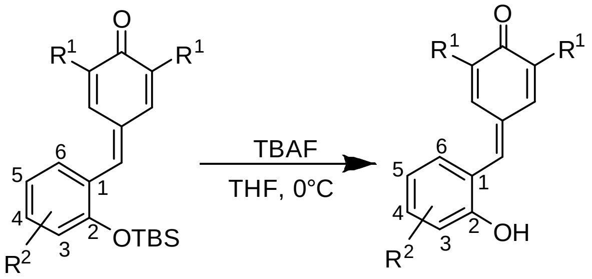

3a: R^1 = *t*-Bu, R^2 = H
3b: R^1 = *t*-Bu, R^2 = 5-Me
3c: R^1 = *t*-Bu, R^2 = 5-Br
3d: R^1 = *t*-Bu, R^2 = 5-Cl
3e: R^1 = *t*-Bu, R^2 = 5-OMe
3f: R^1 = *t*-Bu, R^2 = 5-F
3g: R^1 = *t*-Bu, R^2 = 5-*t*-Bu
3h: R^1 = *t*-Bu, R^2 = 4-Cl
3i: R^1 = *t*-Bu, R^2 = 3-Cl
3j: R^1 = *t*-Bu, R^2 = 6-Cl
3k: R^1 = Me, R^2 = H

To a stirred solution of hydroxy-aldehyde (2.5 mmol, 1.0 eq.), triethylamine (2.98 mmol, 1.2 eq.), and 4-(dimethylamino) pyridine (0.02 mmol, 0.05 eq.) in CH_2Cl_2 (10 mL, 0.25 M) was added *tert*-butyldimethylsilyl chloride (2.98 mmol, 1.2 eq.) in portions at r.t. The resulting mixture was then stirred for 24 h. The reaction was quenched by the addition of sat. sol. $NaHCO_3$ (20 mL). The aqueous phase was extracted with EtOAc (3x15 mL). The combined organic layers were washed with sat. sol. NaCl (2x10 mL) and dried over anhydrous Na_2SO_4. Organic solvents were evaporated under reduce pressure. The protected hydroxy-aldehydes were used without further purification in next reaction.

A solution of phenols (2.1 mmol, 1.1 eq.) and protected hydroxy-aldehydes (1.9 mmol, 1.0 eq.) in toluene (6 mL) was placed in a Dean-Stark apparatus which was heated to reflux. Piperidine (3.8 mmol, 2.0 eq.) in toluene (4mL) was added dropwise slowly within 1 hour. Then, the temperature was elevated to 140°C and stirred overnight (approximately 12h). After that, the reaction mixture was cooled to 120°C and acetic anhydride (3.8 mmol, 2.0 eq.) was added dropwise. The reaction was continued to stir for 30 min and the solution was poured on ice-water and the aqueous phase was extracted with EtOAc (3 ×15 mL). The combined organic phase was washed with sat. sol. NaCl (2x10 mL), dried over anhydrous Na_2SO_4 and the solvents were evaporated under reduced pressure. The crude products were purified using column chromatography (silica gel, heptanes:Et_2O = 100:1 to 20:1).

The protected *p*-QMs (2.9 mmol, 1.0 eq.) were dissolved in THF (29 mL, 0.1 M) and cooled to 0°C. TBAF (1M solution in THF, 2.2 mmol, 1.1 eq.) was added dropwise and the resulting mixture was

[2] Zhao, K.; Zhi, Y.; Shu, T.; Valkonen, A.; Rissanen, K.; Enders, D. *Angew. Chem. Int. Ed.* **2016**, *55*, 12104

stirred for 10 min at 0°C. The reaction was quenched by the addition of sat. sol. NH_4Cl (20 mL) and the aqueous phase was extracted with EtOAc (5x15 mL). The combined organic solvents were washed with sat. sol. NaCl (2x10mL) and dried over anhydrous Na_2SO_4. Solvents were evaporated under reduced pressure to obtain the crude products, which were purified using column chromatography (silica gel, heptanes:EtOAc - 30:1 to 5:1) to afford the desired p-QMs **3a**-**3k**. Analytical data matched those presented in literature.

2.2 Formal (4+1)-Addition of Allenoates to para-Quinone Methides using PPh_3 or chiral PR_3

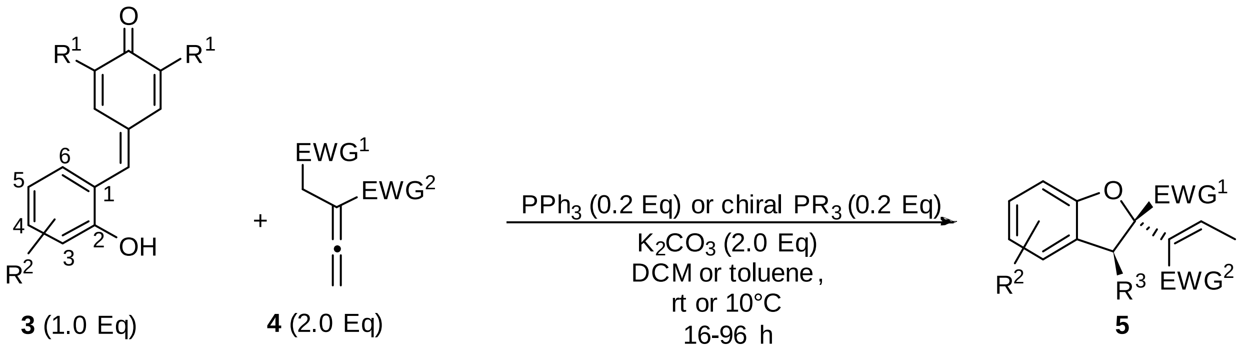

3a: R^1 = *t*-Bu, R^2 = H
3b: R^1 = *t*-Bu, R^2 = 5-Me
3c: R^1 = *t*-Bu, R^2 = 5-Br
3d: R^1 = *t*-Bu, R^2 = 5-Cl
3e: R^1 = *t*-Bu, R^2 = 5-OMe
3f: R^1 = *t*-Bu, R^2 = 5-F
3g: R^1 = *t*-Bu, R^2 = 5-*t*-Bu
3h: R^1 = *t*-Bu, R^2 = 4-Cl
3i: R^1 = *t*-Bu, R^2 = 3-Cl
3j: R^1 = *t*-Bu, R^2 = 6-Cl
3k: R^1 = Me, R^2 = H

4a: EWG^1 = CO_2Et, EWG^2 = CO_2Et
4b: EWG^1 = CO_2Et, EWG^2 = CO_2t-Bu
4c: EWG^1 = CO_2Et, EWG^2 = CO_2Bn

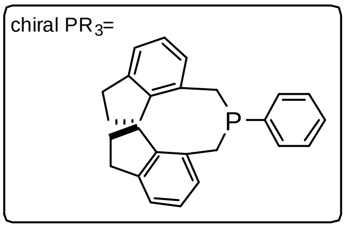

General Racemic Procedure:

The solution of the allenoate **4** (2.0 eq.) in dry CH_2Cl_2 (20 mL per mmol **4**) was added to the mixture of the para-quinone methide **3** (1.0 eq.), K_2CO_3 (2.0 eq.) and PPh_3 (0.2 eq.). The resulting mixture was stirred at in an Argon atmosphere at r.t. for the indicated time. The reaction was diluted with CH_2Cl_2 (5 mL), filtered over a pad of Na_2SO_4 and the filtration cake was washed with CH_2Cl_2 (5x 5 mL). The solvent was removed under reduce pressure and the crude products were purified using column chromatography to give the products **5** in the given yields.

General Chiral Procedure:

The mixture of the para-quinone methide **3** (0.05-0.2 mmol, 1.0 eq.), K_2CO_3 (2.0 eq.) and chiral PR_3 (20 mol%) was cooled to 10°C and a solution of allenoate **4** (2.0 eq.) in toluene (20 mL per mmol **4**) was added. The resulting mixture was stirred in an Ar-atmosphere at 10°C for the indicated time. The reaction was diluted by addition of 5 mL CH_2Cl_2 at 10°C, filtrated over pad of Na_2SO_4 and washed with CH_2Cl_2 (5x5mL). The solvent was removed under reduce pressure and the crude products were purified using column chromatography to give the chiral products **5** in the given yields.

5a: Starting from *p*-QM **3a** (31 mg, 0.1 mmol), allenoate **4a** (40 mg, 0.2 mmol) following the chiral procedure the reaction mixture was stirred for 20 h and the crude product purified using column chromatography (silica gel, heptanes:EtOAc = 20:1 to 10:1) giving the enantioenriched product **5a** as yellow oil (45 mg, 89%, *e.r.*= 94:6). This reaction was also carried out on racemic 1.0 mmol scale giving the product in 86% yield after 20 h.

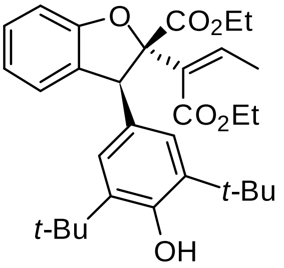

5a

α_D^{23}= +64.6 (c = 0.15, $CHCl_3$) ^{1}H NMR (300 MHz, δ, $CDCl_3$, 298 K): 0.82 (t,*J* = 7.2 Hz, 3H), 1.36 (t, *J* = 7.1 Hz, 3H), 1.36 (s, 18H), 1.89 (d, *J* = 7.2 Hz, 3H), 3.52-3.77 (m, 2H), 4.26-4.36 (m, 2H), 5.11 (s, 1H), 5.24 (s, 1H), 6.40 (q, *J* = 7.1 Hz, 1H), 6.83 (s, 2H), 6.94 (t, *J* = 7.3 Hz, 1H), 7.01-7.09 (m, 2H), 7.19-7.26 (m, 1H) ppm. ^{13}C NMR (75 MHz, δ, $CDCl_3$, 298 K): 13.4, 14.2, 15.5, 30.2, 34.2, 55.8, 60.8, 61.1, 94.0, 110.2, 121.8, 125.7, 126.1, 128.9, 129.4, 130.0, 132.7, 133.2, 135.1, 153.0, 158.1, 167.2, 168.44 ppm. HRMS (ESI): m/z calcd for $C_{31}H_{40}O_6$: 509.2898 $[M+H]^+$; found: 509.2897. HPLC (CHIRAL ART Cellulose-SB, eluent: Hexane:*i*-PrOH = 95:5, 0.5 mL/min, 10 °C, retention times: t_{major} = 9.4 min, t_{minor} = 11.0 min).

5b: Starting from *p*-QM **3a** (0.05 mmol, 15.5 mg) and allenoate **4c** (0.1 mmol, 27.3 mg) following the chiral procedure the reaction mixture was stirred for 24h and crude product was purified using column chromatography (silica gel, heptanes:aceton = 10:1 to 3:1) giving the enantioenriched product **5b** as a pale yellow oil (22.2 mg, 78%, *e.r.*= 94:6). This reaction was also carried out on racemic 0.05 mmol scale giving the product in 81% yield after 16h.

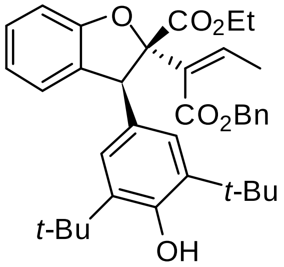

5b

α_D^{23}= +68 (c 0.5, *$CHCl_3$*). ^{1}H NMR (300 MHz, δ, $CDCl_3$, 298 K): 0.71 (t, *J* = 7.1 Hz, 3H), 1.34 (s, 18H), 1.84 (d, *J* = 7.1 Hz, 3H), 3.39 – 3.62 (m, 2H), 5.09 (s, 1H), 5.18 – 5.28 (m, 2H), 5.31 (d, *J* = 12.4 Hz, 1H), 6.41 (q, *J* = 7.1 Hz, 1H), 6.81 (s, 2H), 6.90 (td, *J* = 7.4, 1.0 Hz, 1H), 6.96 – 7.12 (m, 2H), 7.15 – 7.26 (m, 1H), 7.29 – 7.45 (m, 5H) ppm. ^{13}C NMR (75 MHz, δ, $CDCl_3$, 298 K): 13.6, 15.7, 30.4, 34.3, 56.0, 61.3, 66.8, 94.1, 110.3, 121.9, 125.8, 126.2, 128.3, 128.6, 128.9, 129.5, 130.0, 133.1, 133.4, 135.3, 135.9, 153.2, 158.2, 167.2, 168.5

ppm. HRMS (ESI): *m/z* calcd for $C_{36}H_{42}O_6$: 571.3054 $[M+H]^+$; found: 571.3053. HPLC (CHIRAL ART Amylose-SA, eluent: Hexane:*i*-PrOH = 90:10, 0.5 mL/min, 10 °C, retention times: t_{minor} = 9.5 min, t_{major} = 11.6 min).

5c: Starting from *p*-QM **3k** (0.05 mmol, 11.3 mg) and allenoate **4a** (0.1 mmol, 19.8 mg) following the chiral procedure the reaction mixture was stirred for 24h and crude product was purified using column chromatography (silica gel, heptanes:aceton = 10:1 to 1:1) giving the enantioenriched product **5c** as a colourless solid (13 mg, 62%, *e.r.*=88:12). This reaction was also carried out on racemic 0.05 mmol scale giving the product in 81% yield after 24h.

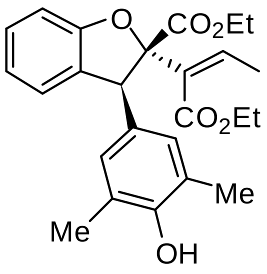

5c

α_D^{24}= +62.8 (c 0.25, *CHCl₃*). ^{1}H NMR (300 MHz, δ, $CDCl_3$, 298 K): 0.89 (t, *J* = 7.1 Hz, 3H), 1.34 (t, *J* = 7.1 Hz, 3H), 1.86 (d, *J* = 7.1 Hz, 3H), 2.14 (s, 6H), 3.60 – 3.81 (m, 2H), 4.20 – 4.39 (m, 2H), 4.50 (s, 1H), 5.20 (s, 1H), 6.36 (q, *J* = 7.1 Hz, 1H), 6.66 (s, 2H), 6.91 (td, *J* = 7.4, 1.0 Hz, 1H), 6.95 – 7.07 (m, 2H), 7.16 – 7.25 (m, 1H) ppm. ^{13}C NMR (75 MHz, δ, $CDCl_3$, 298 K): 13.6, 14.3, 15.6, 15.9, 55.4, 61.0, 61.5, 94.0, 110.5, 122.1, 122.5, 125.6, 128.9, 129.6, 129.7, 131.0, 132.8, 133.3, 151.7, 158.2, 167.3, 168.5 ppm. HRMS (ESI): *m/z* calcd for $C_{25}H_{28}O_6$: 425.1959 $[M+H]^+$; found: 425.1960. HPLC (CHIRAL ART Amylose-SA, eluent: Hexane:*i*-PrOH = 90:10, 0.5 mL/min, 10 °C, retention times: t_{minor} = 13.6 min, t_{major} = 20.9 min).

5d: Starting from *p*-QM **3d** (0.13 mmol, 45 mg) and allenoate **4a** (0.26 mmol, 50 mg) following the chiral procedure the reaction mixture was stirred for 21 h and the crude product was purified using column chromatography (silica gel, heptanes:EtOAc = 20:1 to 7:1) giving the enantioenriched product **5d** as a yellow oil (46 mg, 65%, 92:8). This reaction was also carried out on racemic 0.05 mmol scale giving the product in 65% after 21h.

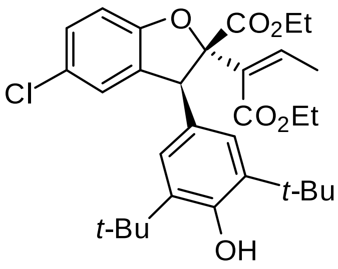

5d

α_D^{22}= +65 (*c* 0.3, *CHCl₃*). ^{1}H NMR (300 MHz, δ, $CDCl_3$, 298 K): 0.79 (t, *J* = 7.2 Hz, 3H), 1.35 (m, 21H), 1.88 (d, *J* = 7.1 Hz, 3H), 3.47 – 3.84 (m, 2H), 4.28 (qd, *J* = 7.1, 1.4 Hz, 2H), 5.13 (s, 1H), 5.18 (s, 1H), 6.35 (q, *J* = 7.1 Hz, 1H), 6.81 (s, 2H), 6.95 (d, *J* = 8.6 Hz, 1H), 7.03 (d, *J* = 2.3 Hz, 1H), 7.16 (dd, *J* = 8.5, 2.3 Hz, 1H) ppm. ^{13}C NMR (75 MHz, δ, $CDCl_3$, 298 K): 13.5, 14.2, 15.5, 30.2, 34.2, 55.7, 60.9, 61.2, 94.6, 111.3, 125.7, 125.9, 126.5, 128.8, 129.3, 131.6, 132.9, 133.0, 135.4, 153.3, 156.6, 166.9, 168.0 ppm. HRMS (ESI): *m/z* calcd for $C_{31}H_{39}ClO_6$: 543.2508 $[M+H]^+$; found: 543.2007. HPLC (CHIRAL ART Cellulose-SB, eluent: Hexane:*i*-PrOH = 98:2, 0.5 mL/min, 10 °C, retention times: t_{major} = 11.9 min, t_{minor} = 14.3 min).

5e: Starting from *p*-QM **3c** (0.05 mmol, 19.5 mg) and allenoate **4a** (0.1 mmol, 19.8 mg) following the chiral procedure the reaction mixture was stirred for 48h and crude product was purified using column chromatography (silica gel, heptanes:EtOAc = 20:1 to 7:1) giving the enantioenriched product **5e** as a yellow oil (21 mg, 72%, *e.r.*= 92:8). This reaction was also carried out on racemic 0.05 mmol scale giving the product in 76% after 19h.

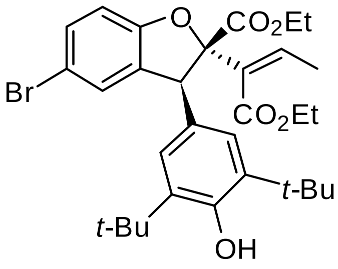

5e

α_D^{23}= +36.3 (*c* 0.4, *CHCl*$_3$) ^{1}H NMR (300 MHz, δ, $CDCl_3$, 298 K): 0.79 (t, *J* = 7.2 Hz, 3H), 1.36 (m, 21H), 1.88 (d, *J* = 7.1 Hz, 3H), 3.48 – 3.85 (m, 2H), 4.28 (qd, *J* = 7.1, 1.3 Hz, 2H), 5.14 (s, 1H), 5.18 (s, 1H), 6.35 (q, *J* = 7.1 Hz, 1H), 6.81 (s, 2H), 6.91 (d, *J* = 8.5 Hz, 1H), 7.17 (dd, *J* = 2.2, 0.9 Hz, 1H), 7.30 (dd, *J* = 8.4, 2.0 Hz, 1H) ppm. ^{13}C NMR (75 MHz, δ, $CDCl_3$, 298 K): 13.7, 14.3, 15.6, 30.3, 34.4, 55.7, 61.0, 61.4, 94.7, 112.0, 113.8, 126.0, 128.7, 129.4, 131.8, 132.2, 133.0, 133.2, 135.5, 153.4, 157.3, 167.0, 168.1 ppm. HRMS (ESI): *m/z* calcd for $C_{31}H_{39}BrO_6$: 587.2003 $[M+H]^+$; found: 587.2001. HPLC (CHIRAL ART Cellulose-SB, eluent: Hexane:*i*-PrOH = 95:5, 0.5 mL/min, 10 °C, retention times: t_{major} = 10.1 min, t_{minor} = 11.8 min).

5f: Starting from *p*-QM **3h** (0.2 mmol, 69 mg) and allenoate **4a** (0.4 mmol, 74 mg) following the chiral procedure the reaction mixture was stirred for 68 h and crude product was purified using column chromatography (silica gel, heptanes:EtOAc = 20:1 to 7:1) giving the enantioenriched product **5f** as a colourless oil (76 mg, 70%, *e.r.*= 94:6). This reaction was also carried out on racemic 0.05 mmol scale giving the product in 67% after 48h.

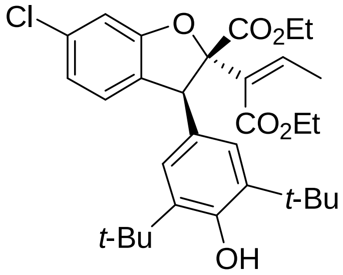

5f

α_D^{22}= +97.1 (c 0.35, *CHCl*$_3$). ^{1}H NMR (300 MHz, δ, $CDCl_3$, 298 K): 0.79 (t, *J* = 7.2 Hz, 3H), 1.35 (s, 21H), 1.89 (d, *J* = 7.1 Hz, 3H), 3.47 – 3.76 (m, 2H), 4.28 (qd, *J* = 7.2, 2.1 Hz, 2H), 5.13 (s, 1H), 5.18 (s, 1H), 6.36 (q, *J* = 7.1 Hz, 1H), 6.81 (s, 2H), 6.89 (dd, *J* = 8.0, 1.8 Hz, 1H), 6.96 (dd, *J* = 8.0, 0.9 Hz, 1H), 7.02 (d, *J* = 1.8 Hz, 1H) ppm. ^{13}C NMR (75 MHz, δ, $CDCl_3$, 298 K): 13.7, 14.3, 15.6, 30.4, 34.4, 55.4, 61.0, 61.4, 95.0, 111.1, 122.1, 126.1, 126.4, 128.4, 129.5, 133.1, 133.1, 134.1, 135.4, 153.3, 159.0, 167.1, 168.1 ppm. HRMS (ESI): *m/z* calcd for $C_{31}H_{39}ClO_6$: 543.2508 $[M+H]^+$; found: 543.2508. HPLC (CHIRAL ART Cellulose-SB, eluent: Hexane:*i*-PrOH = 100:1, 0.5 mL/min, 10 °C, retention times: t_{major} = 12.4 min, t_{minor} = 15.4 min).

5g: Starting from *p*-QM **3i** (0.05 mmol, 17.2 mg) and allenoate **4a** (0.1 mmol, 19.8 mg) following the chiral procedure the reaction mixture was stirred for 42h and crude product was purified using column chromatography (silica gel, heptanes:EtOAc = 15:1 to 4:1) giving the enantioenriched product **5g** as a colourless oil (16 mg, 59%, *e.r.*= 79:21). This reaction was also carried out on racemic 0.05 mmol scale giving the product in 74% after 18h.

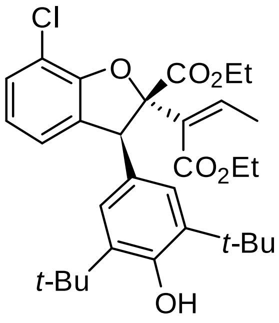

5g

α_D^{24}= +36 (c 0.25, *CHCl₃*). ^{1}H NMR (300 MHz, δ, $CDCl_3$, 298 K): 0.79 (t, *J* = 7.2 Hz, 3H), 1.35 (m, 21H), 1.88 (d, *J* = 7.1 Hz, 3H), 3.51 – 3.72 (m, 2H), 4.20 – 4.41 (m, 2H), 5.13 (s, 1H), 5.37 (s, 1H), 6.36 (q, *J* = 7.1 Hz, 1H), 6.79 – 6.90 (m, 3H), 6.89 – 6.99 (m, 1H), 7.19 – 7.23 (m, 1H) ppm. ^{13}C NMR (75 MHz, δ, $CDCl_3$, 298 K): 13.7, 14.3, 15.6, 30.4, 34.4, 56.8, 61.0, 61.3, 94.7, 115.6, 122.7, 124.2, 126.2, 129.2, 129.4, 131.2, 132.4, 132.9, 135.4, 153.3, 154.5, 167.2, 167.8 ppm. HRMS (ESI): *m/z* calcd for $C_{31}H_{39}ClO_6$: 543.2508 $[M+H]^+$; found: 543.2507. HPLC (CHIRAL ART Cellulose-SB, eluent: Hexane:*i*-PrOH = 100:1, 0.5 mL/min, 10 °C, retention times: t_{major} = 11.9 min, t_{minor} = 18.5 min).

5h: Starting from *p*-QM **3j** (0.05 mmol, 17.2 mg) and allenoate **4a** (0.1 mmol, 19.8 mg). following the racemic procedure the reaction mixture was stirred for 30h and crude product was purified using column chromatography (silica gel, heptanes:EtOAc = 20:1 to 4:1) giving the racemic product **5h** as a yellow oil (22 mg, 81%).

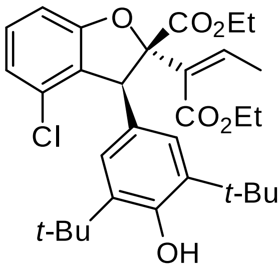

5h

^{1}H NMR (300 MHz, δ, $CDCl_3$, 298 K) δ 0.80 (t, *J* = 7.1 Hz, 3H), 1.34 (m, 21H), 1.88 (d, *J* = 7.1 Hz, 3H), 3.55 – 3.78 (m, 2H), 4.29 (q, *J* = 7.1 Hz, 2H), 5.09 (s, 1H), 5.20 (s, 1H), 6.35 (q, *J* = 7.1 Hz, 1H), 6.80 (bs, 2H), 6.88 (dd, *J* = 8.0, 0.9 Hz, 1H), 6.94 (d, *J* = 8.0 Hz, 1H), 7.15 (t, *J* = 8.0 Hz, 1H) ppm. ^{13}C NMR (75 MHz, δ, $CDCl_3$, 298 K): 13.7, 14.3, 15.7, 30.4, 34.3, 55.2, 61.1, 61.4, 94.3, 109.0, 122.4, 125.9, 127.7, 128.4, 130.1, 131.2, 132.9, 133.3, 135.2, 153.2, 158.7, 167.0, 168.0 ppm. HRMS (ESI): *m/z* calcd for $C_{31}H_{39}ClO_6$: 543.2508 $[M+H]^+$; found: 543.2507.

5i: Starting from *p*-QM **3b** (0.05 mmol, 16.2 mg) and allenoate **4a** (0.1 mmol, 19.8 mg) following the chiral procedure the reaction mixture was stirred for 16h and crude product was purified using column chromatography (silica gel, heptanes:EtOAc = 20:1 to 7:1) giving the enantioenriched product **5i** as a yellow oil (17 mg, 65%, *e.r.*= 94:6). This reaction was also carried out on racemic 0.05 mmol scale giving the product in 85% after 16h.

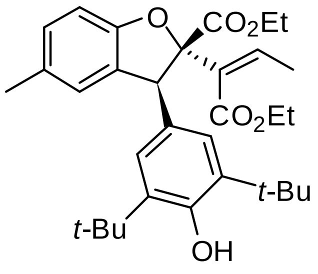

5i

α_D^{21}= +50.6 (*c* 0.15, *CHCl*$_3$). ^{1}H NMR (300 MHz, δ, $CDCl_3$, 298 K): 0.79 (t, *J* = 7.1 Hz, 3H), 1.35 (s, 21H), 1.86 (d, *J* = 7.1 Hz, 3H), 2.23 (s, 3H), 3.48 – 3.76 (m, 2H), 4.28 (q, *J* = 7.4 Hz, 2H), 5.10 (s, 1H), 5.16 (s, 1H), 6.36 (q, *J* = 7.1 Hz, 1H), 6.82 (s, 2H), 6.86 – 7.03 (m, 3H) ppm. ^{13}C (75 MHz, δ, $CDCl_3$, 298 K): 13.7, 14.3, 15.6, 21.0, 30.4, 34.4, 56.0, 60.9, 61.2, 94.2, 109.9, 126.1, 126.2, 129.3, 129.5, 130.3, 131.2, 132.7, 133.5, 135.2, 153.2, 156.1, 167.3, 168.6. HRMS (ESI): *m/z* calcd for $C_{32}H_{42}O_6$: 523.3054 $[M+H]^+$; found: 523.3054. HPLC (CHIRAL ART Cellulose-SB, eluent: Hexane:*i*-PrOH = 95:5, 0.5 mL/min, 10 °C, retention times: t_{major} = 9.6 min, t_{minor} = 11.1 min).

5j: Starting from *p*-QM **3g** (0.05 mmol, 18.3 mg) and allenoate **4a** (0.1 mmol, 19.8 mg) following the chiral procedure the reaction mixture was stirred for 19h and crude product was purified using column chromatography (silica gel, heptanes:EtOAc = 20:1 to 4:1) giving the enantioenriched product **5j** as a colourless oil (22 mg, 78%, *e.r.*= 94:6). This reaction was also carried out on racemic 0.05 mmol scale giving the product in 75% after 19h.

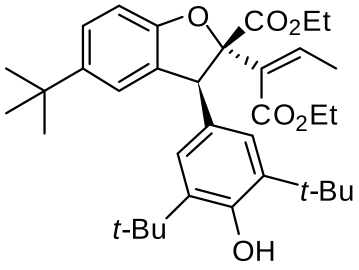

5j

α_D^{22}= +21 (c 0.3, *CHCl*$_3$). ^{1}H NMR (300 MHz, δ, $CDCl_3$, 298 K): 0.78 (t, *J* = 7.1 Hz, 3H), 1.25 (s, 9H), 1.34 (m, 21H), 1.89 (d, *J* = 7.1 Hz, 3H), 3.60 (ddq, *J* = 38.0, 10.7, 7.2 Hz, 2H), 4.30 (q, *J* = 7.2 Hz, 2H), 5.10 (s, 1H), 5.26 (s, 1H), 6.39 (q, *J* = 7.1 Hz, 1H), 6.80 (s, 2H), 6.92 (d, *J* = 8.4 Hz, 1H), 7.06 (d, *J* = 2.1 Hz, 1H), 7.23 (dd, *J* = 8.5, 2.1 Hz, 1H) ppm. ^{13}C NMR (75 MHz, δ, $CDCl_3$, 298 K): 13.7, 14.3, 15.6, 30.4, 31.8, 34.4, 34.6, 56.1, 60.9, 61.2, 94.4, 109.3, 122.9, 125.7, 126.4, 128.5, 130.1, 132.9, 133.4, 135.1, 144.9, 153.1, 156.3, 167.5, 168.8 ppm. HRMS (ESI): *m/z* calcd for $C_{35}H_{48}O_6$: 565.3524 $[M+H]^+$; found: 565.3522. HPLC (CHIRAL ART Cellulose-SB, eluent: Hexane:*i*-PrOH = 98:2, 0.5 mL/min, 10 °C, retention times: t_{major} = 10.5 min, t_{minor} = 12.5 min).

5k: Starting from *p*-QM **3f** (0.05 mmol, 16.4 mg) and allenoate **4a** (0.1 mmol, 19.8 mg) following the chiral procedure the reaction mixture was stirred for 17h and crude product was purified using column chromatography (silica gel, heptanes:EtOAc = 20:1 to 4:1) giving the enantioenriched product **5k** as a yellow oil (20 mg, 77%, *e.r.*= 92: 8). This reaction was also carried out on racemic 0.05 mmol scale giving the product in 84% after 12h.

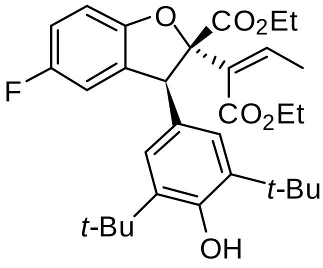

5k

α_D^{22}= +101.7 (c 0.35, *CHCl3*). ¹H NMR (300 MHz, δ, $CDCl_3$, 298 K): 0.79 (t, *J* = 7.1 Hz, 3H), 1.35 (m, 21H), 1.88 (d, *J* = 7.1 Hz, 3H), 3.49 – 3.77 (m, 2H), 4.29 (qd, *J* = 7.1, 1.3 Hz, 2H), 5.13 (s, 1H), 5.19 (s, 1H), 6.36 (q, *J* = 7.1 Hz, 1H), 6.76 (dd, *J* = 7.9, 2.6 Hz, 1H), 6.81 (s, 2H), 6.82 – 6.99 (m, 2H) ppm. ^{13}C NMR (75 MHz, δ, $CDCl_3$, 298 K): 13.7, 14.3, 15.6, 30.3, 34.4, 56.0, 61.0, 61.3, 94.8, 110.7 (d, *J* = 8.5 Hz), 112.7 (d, *J* = 25.0 Hz), 115.3 (d, *J* = 24.3 Hz), 126.1, 129.4, 131.1 (d, *J* = 8.7 Hz), 133.0, 133.2, 135.5, 153.4, 154.1, 158.4 (d, *J* = 238.3 Hz), 167.2, 168.3 ppm. ^{19}F NMR (282 MHz, δ, $CDCl_3$, 298 K): -122.7 (s, 1F) ppm. HRMS (ESI): *m/z* calcd for $C_{31}H_{39}FO_6$: 527.2803 $[M+H]^+$; found: 527.2802. HPLC (CHIRAL ART Cellulose-SB, eluent: Hexane:*i*-PrOH = 99:1, 0.5 mL/min, 10 °C, retention times: t_{major} = 14.9 min, t_{minor} = 18.6 min).

5l: Starting from *p*-QM **3f** (0.05 mmol, 16.4 mg) and allenoate **4b** (0.1 mmol, 22.6 mg). following the chiral procedure the reaction mixture was stirred for 96h and crude product was purified using column chromatography (silica gel, heptanes:aceton = 30:1 to 10:1) giving the enantioenriched product **5l** as a pale yellow oil (17 mg, 61%, *e.r.*= 83:17). This reaction was also carried out on racemic 0.05 mmol scale giving the product in 65% after 96h.

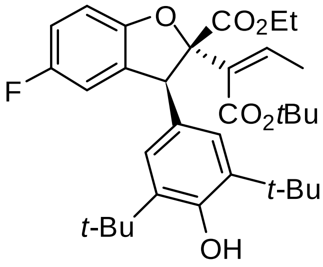

5l

α_D^{24}= +55.6 (c 0.5, *CHCl3*). ¹H NMR (300 MHz, δ, $CDCl_3$, 298 K): 0.75 (t, *J* = 7.1 Hz, 3H), 1.35 (s, 18H), 1.54 (s, 9H), 1.87 (d, *J* = 7.1 Hz, 3H), 3.65 (q, *J* = 7.2 Hz, 2H), 5.12 (s, 1H), 5.19 (s, 1H), 6.27 (q, *J* = 7.0 Hz, 1H), 6.70 – 6.80 (m, 1H), 6.82 (bs, 2H), 6.84 – 6.97 (m, 2H) ppm. ^{13}C NMR (75 MHz, δ, $CDCl_3$, 298 K): 13.7, 15.4, 28.3, 30.4, 34.4, 55.9, 61.3, 82.0, 94.9, 110.6 (d, *J* = 8.5 Hz), 112.7 (d, *J* = 24.8 Hz), 115.2 (d, *J* = 24.2 Hz), 126.0, 129.6, 131.2 (d, *J* = 8.4 Hz), 131.3, 134.5, 135.5, 153.3, 154.2, 158.3 (d, *J* = 238.0 Hz), 166.3, 168.3 ppm. ^{19}F NMR (282 MHz, δ, $CDCl_3$, 298 K): -123.0 (s, 1F) ppm. HRMS (ESI): *m/z* calcd for $C_{33}H_{43}FO_6$: 555.3116 $[M+H]^+$; found: 555.3115. HPLC (CHIRAL ART Amylose-SA, eluent: Hexane:*i*-PrOH = 98:2, 0.5 mL/min, 10 °C, retention times: t_{minor} = 9.2 min, t_{major} = 13.3 min).

5m: Starting from *p*-QM **3e** (0.14 mmol, 48 mg) and allenoate **4a** (0.28 mmol, 52 mg) following the chiral procedure the reaction mixture was stirred for 17h and crude product was purified using column chromatography (silica gel, heptanes:EtOAc = 20:1 to 4:1) giving the enantioenriched product **5m** as a yellow oil (60 mg, 82%, *e.r.*= 92:8). This reaction was also carried out on racemic 0.05 mmol scale giving the product in 85% after 22h.

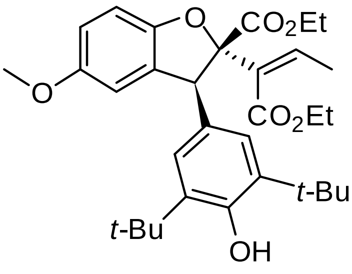

5m

α_D^{22}= +62 (*c* 0.45, *CHCl3*). ^{1}H NMR (300 MHz, δ, $CDCl_3$, 298 K): 0.80 (t, *J* = 7.2 Hz, 3H), 1.35 (m, 21H), 1.86 (d, *J* = 7.1 Hz, 3H), 3.49 – 3.72 (m, 2H), 3.71 (s, 3H), 4.17 – 4.49 (m, 2H), 5.10 (s, 1H), 5.17 (s, 1H), 6.36 (q, *J* = 7.1 Hz, 1H), 6.62 (d, *J* = 2.7 Hz, 1H), 6.75 (dd, *J* = 8.7, 2.7 Hz, 1H), 6.83 (s, 2H), 6.93 (d, *J* = 8.7 Hz, 1H) ppm. ^{13}C NMR (75 MHz, δ, $CDCl_3$, 298 K): 13.7, 14.3, 15.6, 30.4, 34.4, 56.1, 56.3, 60.9, 61.2, 94.4, 110.6, 111.1, 114.6, 126.1, 129.9, 130.4, 132.8, 133.4, 135.3, 152.3, 153.2, 155.2, 167.3, 168.6 ppm. HRMS (ESI): *m/z* calcd for $C_{32}H_{42}O_7$: 539.3003 $[M+H]^+$; found: 539.3004. HPLC (CHIRAL ART Cellulose-SB, eluent: Hexane:*i*-PrOH = 95:5, 0.5 mL/min, 10 °C, retention times: t_{major} = 12.0 min, t_{minor} = 14.1 min).

5n: Starting from *p*-QM **3e** (0.05 mmol, 17.0 mg) and allenoate **4b** (0.1 mmol, 22.6 mg) following the chiral procedure the reaction mixture was stirred for 96h and crude product was purified using column chromatography (silica gel, heptanes:EtOAc = 20:1 to 4:1) giving the enantioenriched product **5n** as a colourless oil (17 mg, 60%, *e.r.*= 85:15). This reaction was also carried out on racemic 0.05 mmol scale giving the product in 55% after 96h.

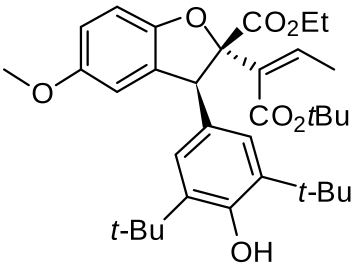

5n

α_D^{24}= +35.7 (c 0.45, *CHCl3*). ^{1}H NMR (300 MHz, δ, $CDCl_3$, 298 K): 0.75 (t, *J* = 7.1 Hz, 3H), 1.35 (s, 18H), 1.54 (s, 9H), 1.85 (d, *J* = 7.1 Hz, 3H), 3.65 (q, *J* = 7.1 Hz, 2H), 3.70 (s, 3H), 5.10 (s, 1H), 5.18 (s, 1H), 6.27 (q, *J* = 7.0 Hz, 1H), 6.61 (d, *J* = 2.7 Hz, 1H), 6.74 (dd, *J* = 8.7, 2.6 Hz, 1H), 6.83 (bs, 2H), 6.91 (d, *J* = 8.7 Hz, 1H) ppm. ^{13}C NMR (75 MHz, δ, $CDCl_3$, 298 K): 13.8, 15.4, 28.3, 30.4, 34.4, 56.1, 56.2, 61.2, 81.8, 94.6, 110.5, 111.1, 114.5, 126.1, 130.1, 130.5, 131.0, 134.6, 135.3, 152.4, 153.2, 155.1, 166.5, 168.6 ppm. HRMS (ESI): *m/z* calcd for $C_{34}H_{46}O_7$: 567.3316 $[M+H]^+$; found: 567.3315. HPLC (CHIRAL ART Amylose-SA, eluent: Hexane:*i*-PrOH = 95:5, 0.5 mL/min, 10 °C, retention times: t_{minor} = 9.1 min, t_{major} = 11.6 min).

5o: Starting from *p*-QM **3e** (0.05 mmol, 17 mg) and allenoate **4c** (0.1 mmol, 27.3 mg) following the chiral procedure the reaction mixture was stirred for 24h and crude product was purified using column chromatography (silica gel, heptanes:aceton = 10:1 to 3:1) giving the enantioenriched product **5o** as a pale yellow oil (20.1 mg, 76%, *e.r.*= 95:5). This reaction was also carried out on racemic 0.05 mmol scale giving the product in 50% after 24h.

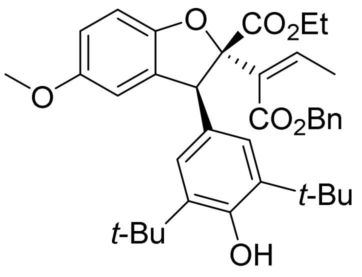

5o

α_D^{23}= +66 (c 0.5, *CHCl*$_3$). ^{1}H NMR (300 MHz, δ, $CDCl_3$, 298 K): 0.71 (t, *J* = 7.1 Hz, 3H), 1.34 (s, 18H), 1.83 (d, *J* = 7.1 Hz, 3H), 3.42 – 3.60 (m, 2H), 3.71 (s, 3H), 5.10 (s, 1H), 5.18 (s, 1H), 5.23 (d, *J* = 12.4 Hz, 1H), 5.31 (d, *J* = 12.4 Hz, 1H), 6.39 (q, *J* = 7.1 Hz, 1H), 6.62 (d, *J* = 2.7 Hz, 1H), 6.74 (dd, *J* = 8.7, 2.7 Hz, 1H), 6.82 (bs, 2H), 6.91 (d, *J* = 8.7 Hz, 1H), 7.36 (m, 5H) ppm. ^{13}C NMR (75 MHz, δ, $CDCl_3$, 298 K): 13.6, 15.7, 30.4, 34.3, 56.1, 56.3, 61.2, 66.8, 94.4, 110.6, 111.1, 114.6, 126.2, 128.3, 128.6, 129.8, 130.3, 133.1, 133.2, 135.3, 135.9, 152.3, 153.2, 155.2, 167.2, 168.5 ppm. HRMS (ESI): *m/z* calcd for $C_{37}H_{44}O_7$: 601.3160 $[M+H]^+$; found: 601.3162. HPLC (CHIRAL ART Amylose-SA, eluent: Hexane:*i*-PrOH = 90:10, 0.5 mL/min, 10 °C, retention times: t_{minor} = 11.3 min, t_{major} = 15.1 min).

2.3 Debutylation of Dihydrobenzofurane 5a

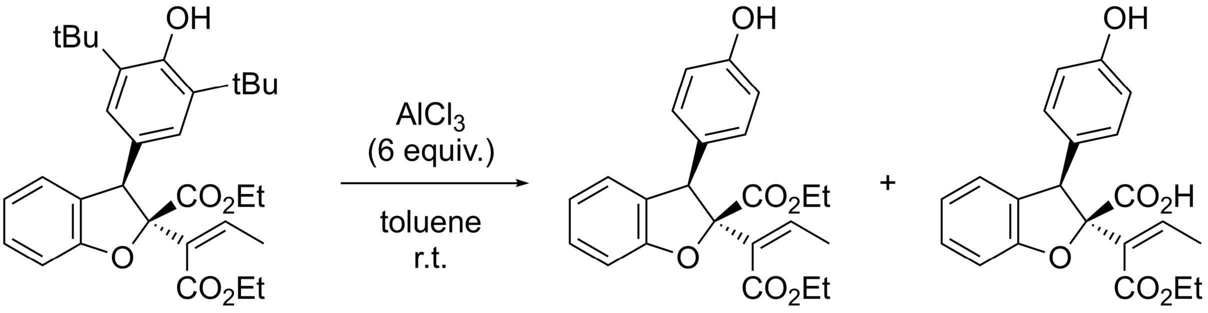

5a **6a** **7a**

3 h: < 5% conversion (traces of **6a**
24 h: ca. 40% conversion (traces of **6a** + 30% **7a**)
72 h: complete conversion (traces of **6a** + 50-60% **7a**)

A mixture of racemic compound **5a** (51 mg, 0.1 mmol) and $AlCl_3$ (80 mg, 6 equiv.) in toluene (4 mL) was stirred at room temperature for 72 h. MS analysis of the reaction mixture shows disappearance of the starting material, traces of **6a** (MS (ESI): m/z calcd for $C_{23}H_{24}O_6$: 397.16 [M+H]+; found: 397.14) and formation of **7a**. The mixture was quenched with H2O (4 mL), extracted with EtOAc (3x) and evaporated to dryness. 1H NMR analysis of the crude product shows around 50-60% 7a accompanied with unidentified side products. Purification by column chromatography (CH_2Cl_2:MeOH = 20:1) gave the acid **7a** as an oily residue in 48% (18 mg); ^{1}H NMR (500 MHz, δ, $CDCl_3$, 298 K): 1.36 (t,

J = 7.1 Hz, 3H), 1.94 (d, J = 7.0 Hz, 3H), 4.37 (q, J = 7.1 Hz, 2H), 4.99 (s, 1H), 6.63-6.71 (m, 3H), 6.88 (d, J = 8.4 Hz, 2H), 6.95 (t, J = 7.4 Hz, 1H), 6.98-7.07 (m, 2H), 7.21-7.26 (m, 1H) ppm. ^{13}C NMR (125 MHz, δ, $CDCl_3$, 298 K): 14.2, 16.0, 55.6, 62.1, 94.0, 110.6, 115.6, 122.8, 125.8, 129.2, 129.3, 130.5, 130.9, 131.8, 137.5, 155.3, 157.4, 168.8, 169.5 ppm; MS (ESI): m/z calcd for $C_{21}H_{20}O_6$: 367.12 [M-H+]; found: 367.08.

3 Copies of ^{1}H and ^{13}C spectra

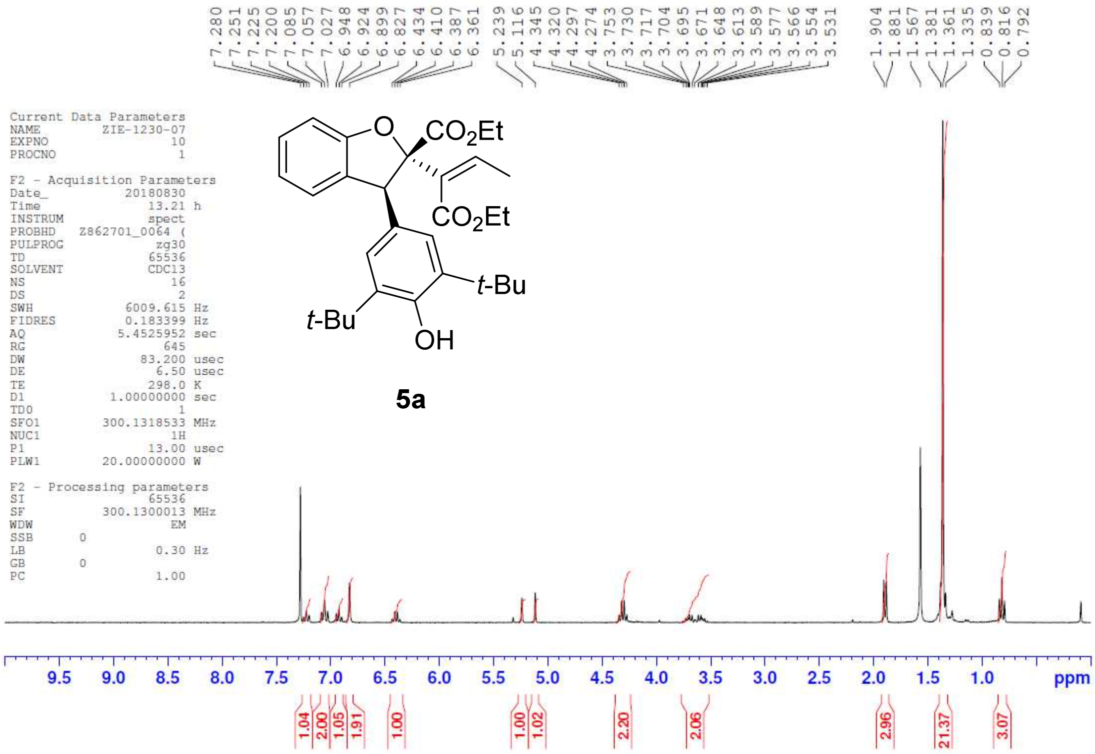

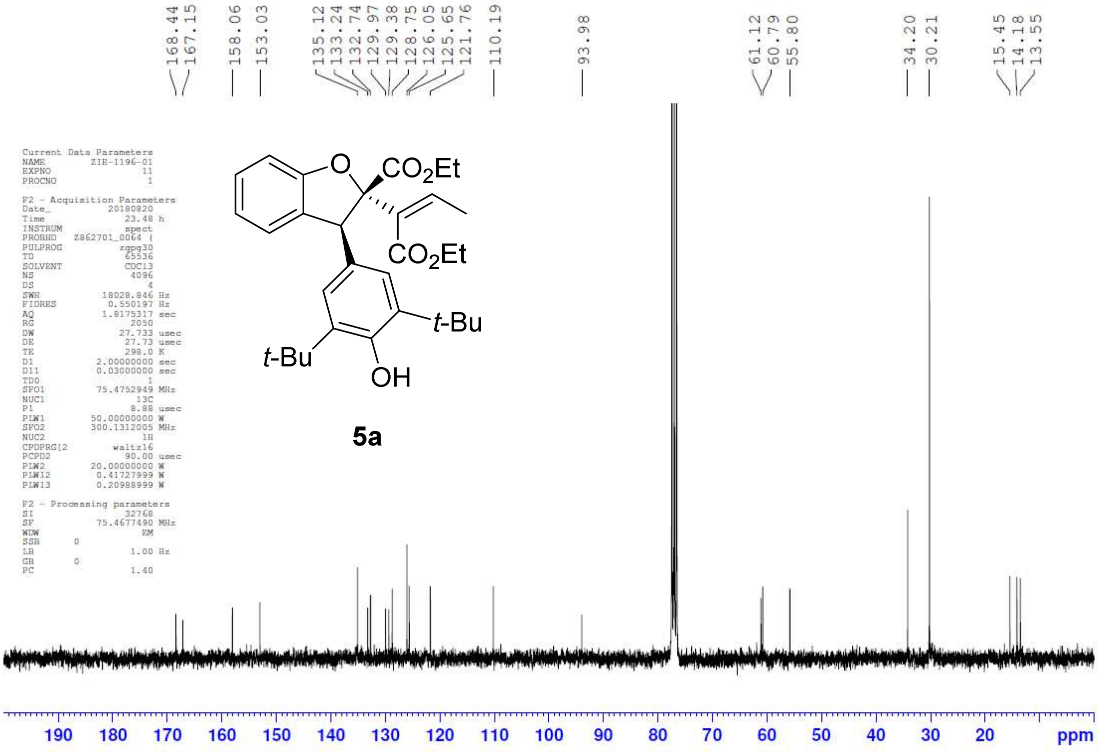

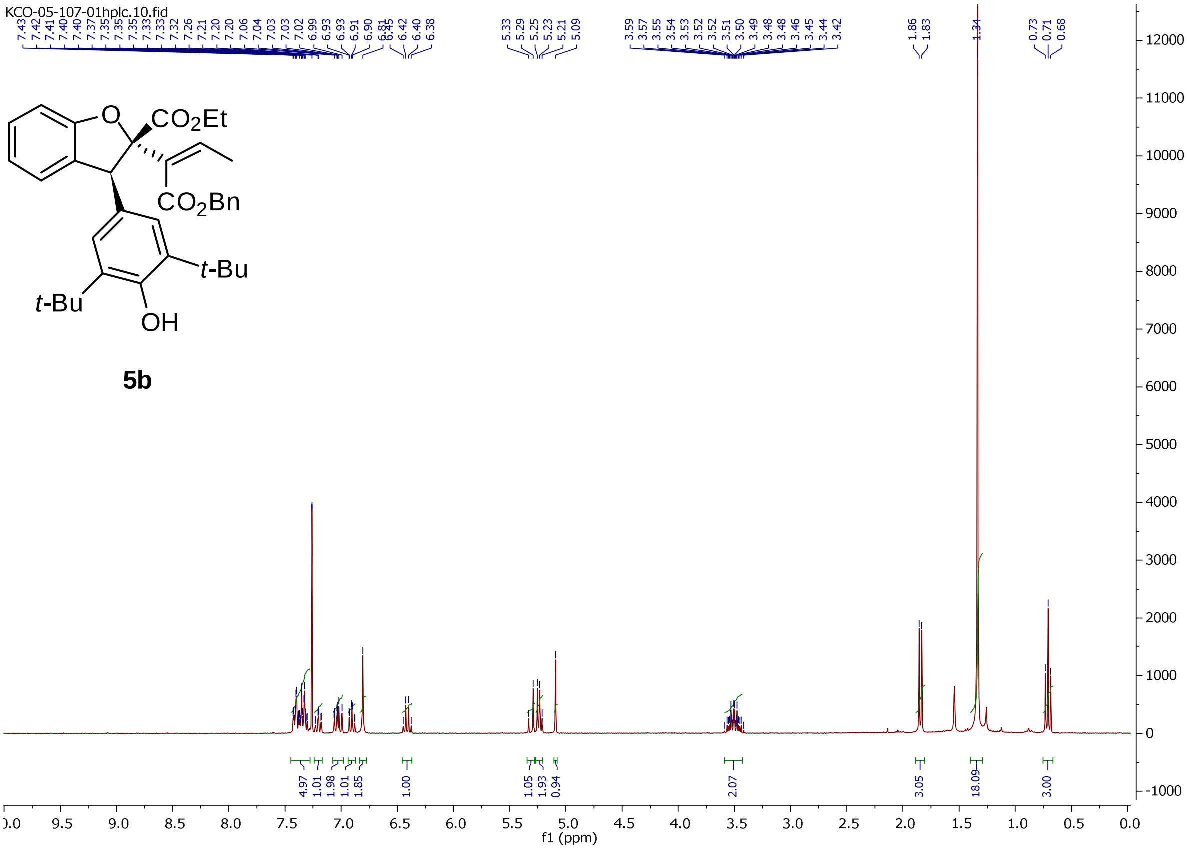
KCO-05-107-01hplc.10.fid
CO2Et
CO2Bn
t-Bu
t-Bu
OH
5b
f1 (ppm)

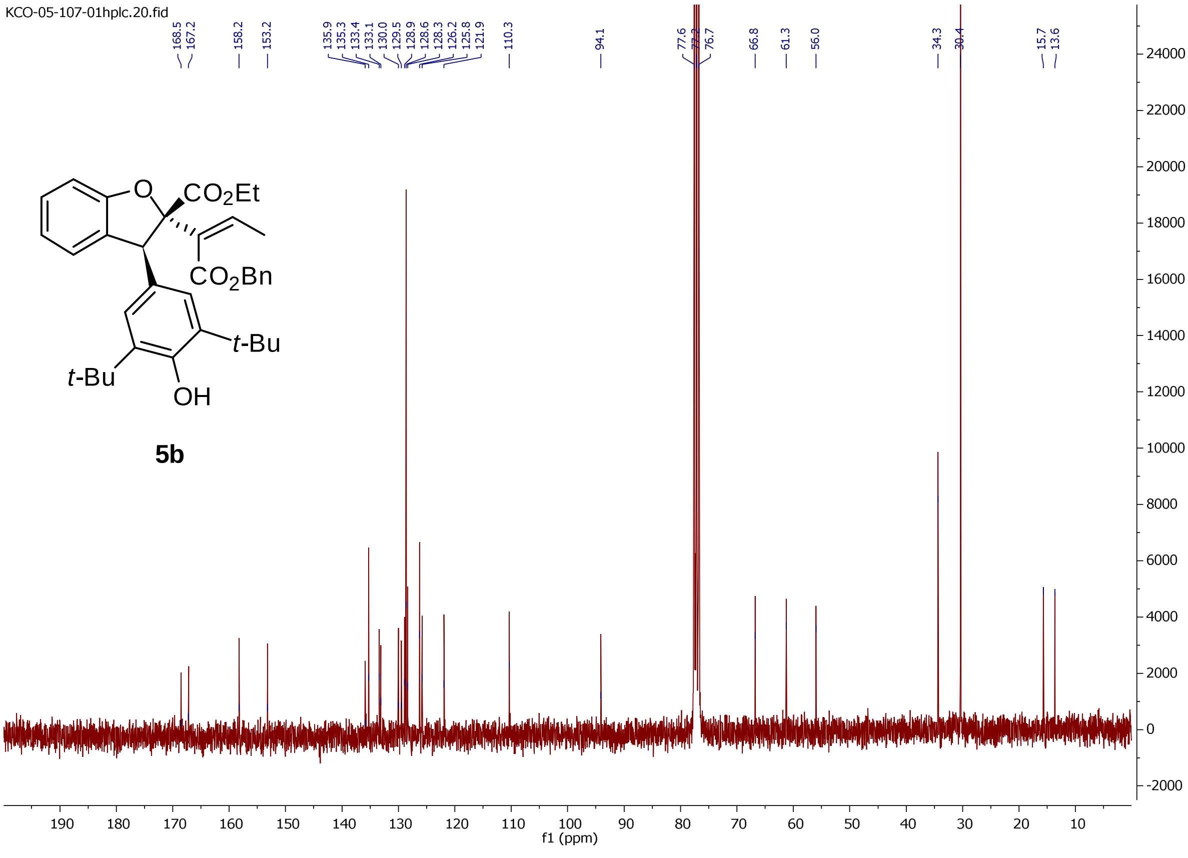
KCO-05-107-01hplc.20.fid
CO2Et
CO2Bn
t-Bu
t-Bu
OH
5b
f1 (ppm)

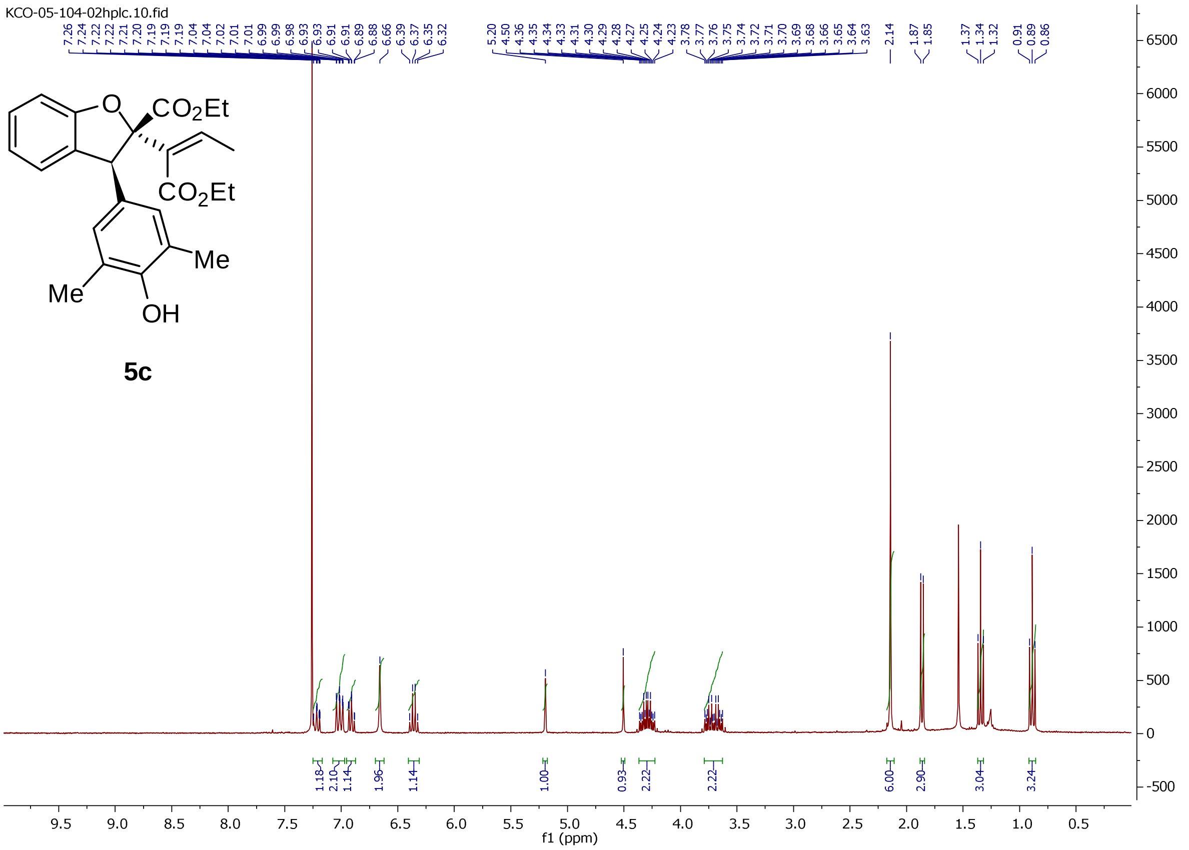
KCO-05-104-02hplc.10.fid
CO2Et
CO2Et
Me
Me
OH
5c
f1 (ppm)

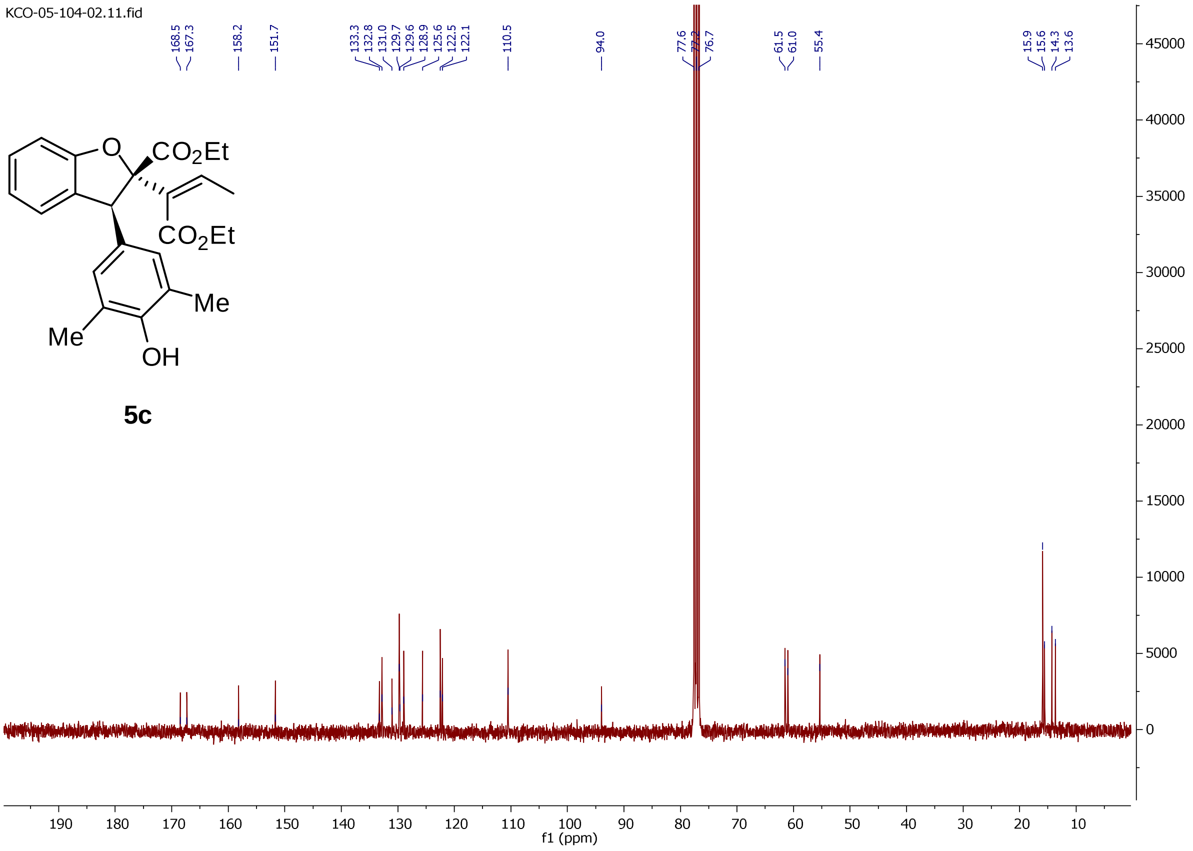
KCO-05-104-02.11.fid
CO2Et
CO2Et
Me
Me
OH
5c
f1 (ppm)

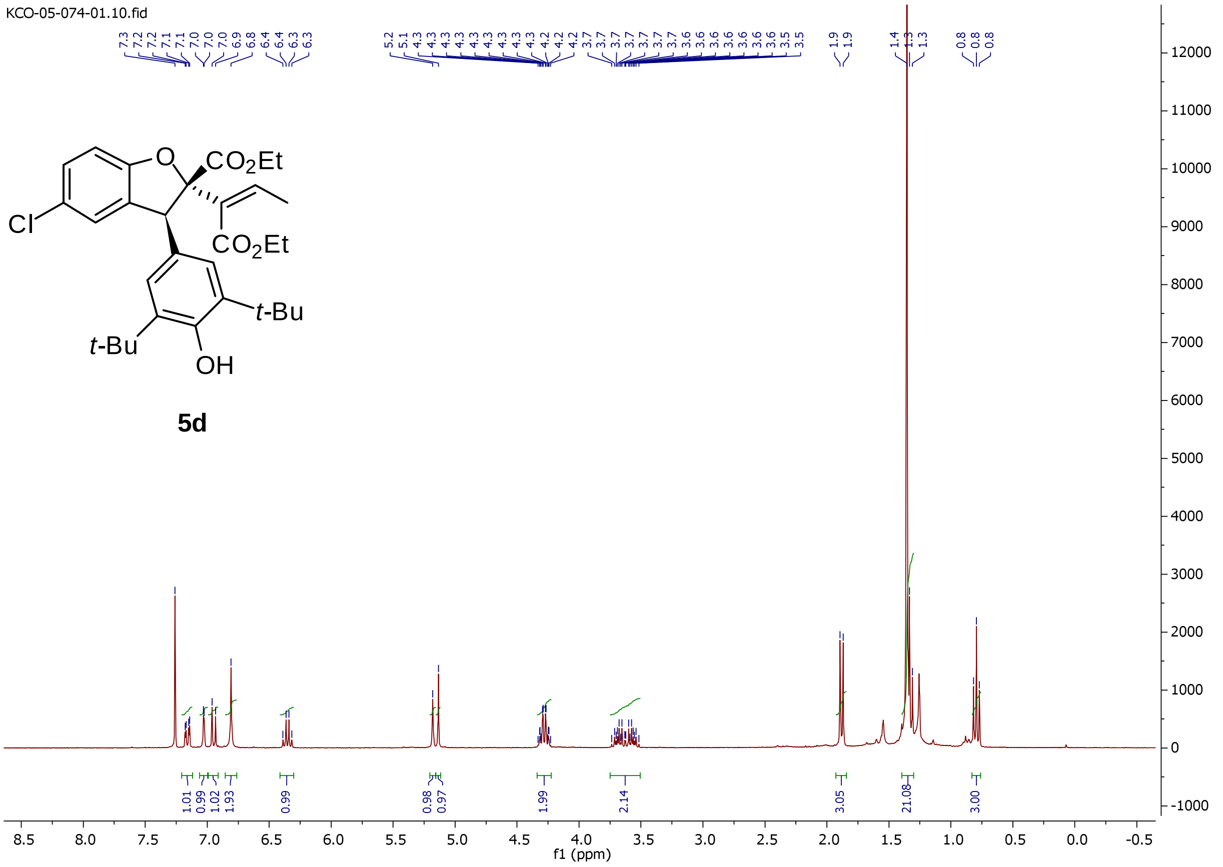
KCO-05-074-01.10.fid
7.3 7.2 7.2 7.1 7.1 7.0 7.0 7.0 6.9 6.8 6.4 6.4 6.3 6.3
5.2 5.1 4.3 4.3 4.3 4.3 4.3 4.3 4.3 4.3 4.2 4.2 4.2 3.7 3.7 3.7 3.7 3.7 3.7 3.7 3.6 3.6 3.6 3.6 3.6 3.6 3.6 3.5
1.9 1.9
1.4 1.3 1.3
0.8 0.8 0.8
CO2Et
Cl
CO2Et
t-Bu
t-Bu
OH
5d
1.01 0.99 1.02 1.93 0.99 0.98 0.97 1.99 2.14 3.05 21.08 3.00
f1 (ppm)

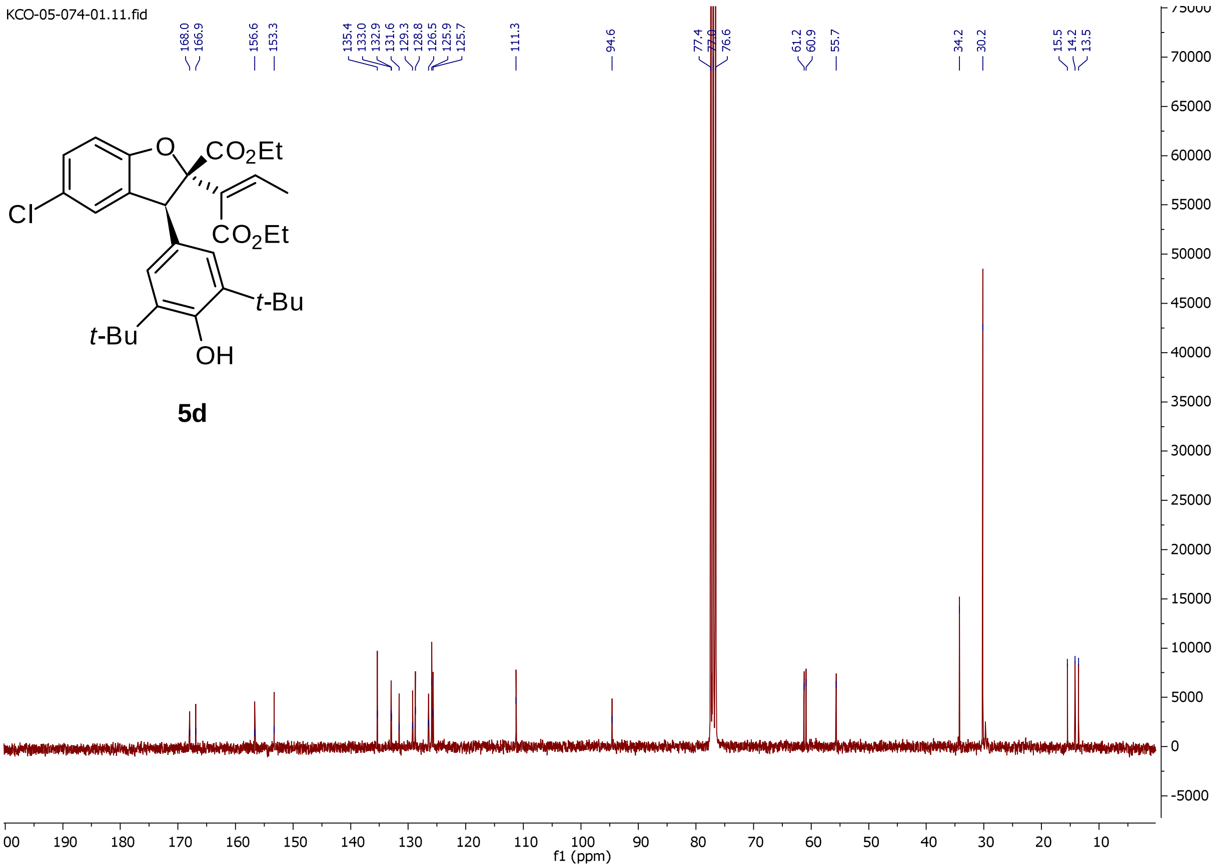
KCO-05-074-01.11.fid
168.0 166.9 156.6 153.3
135.4 133.0 132.9 131.6 129.3 128.8 126.5 125.9 125.7
111.3
94.6
77.4 77.0 76.6
61.2 60.9 55.7
34.2 30.2
15.5 14.2 13.5
CO2Et
Cl
CO2Et
t-Bu
t-Bu
OH
5d
f1 (ppm)

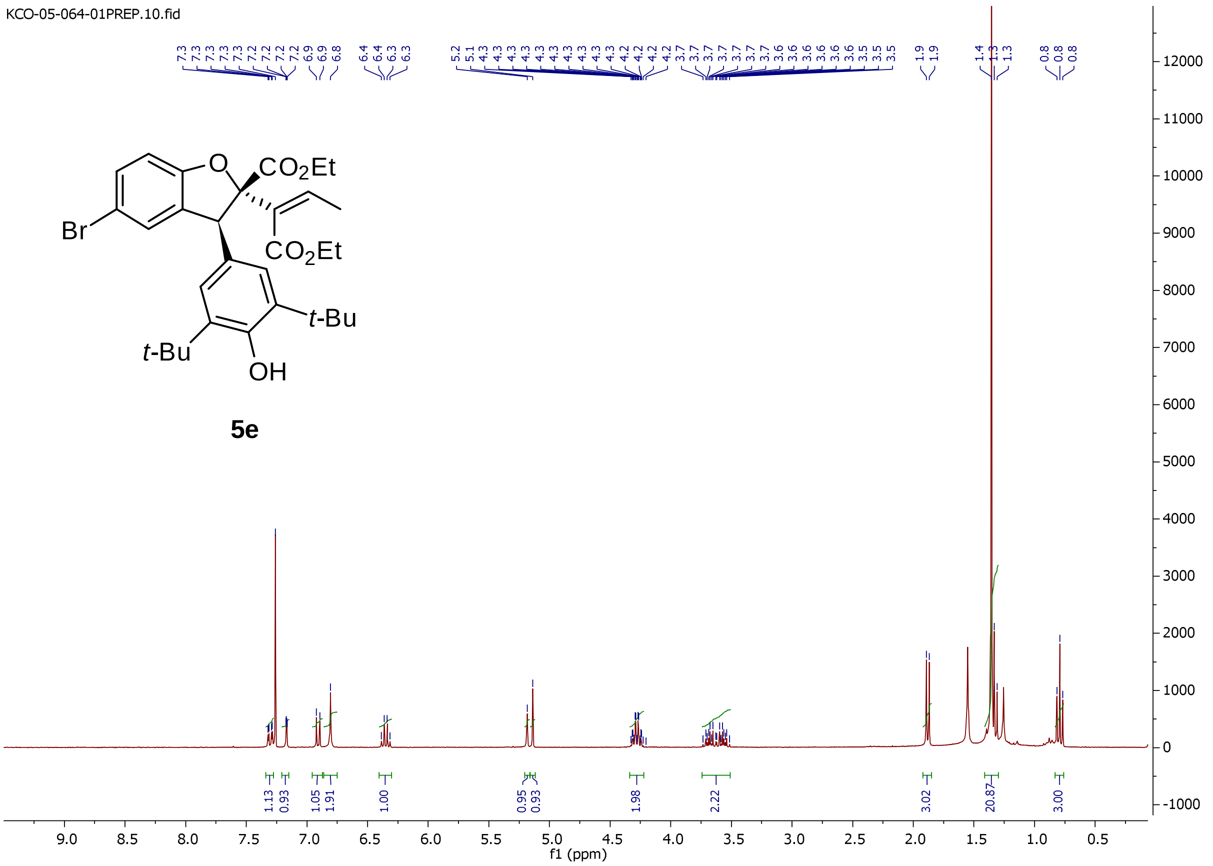
KCO-05-064-01PREP.10.fid
CO2Et
Br
CO2Et
t-Bu
t-Bu
OH
5e
f1 (ppm)

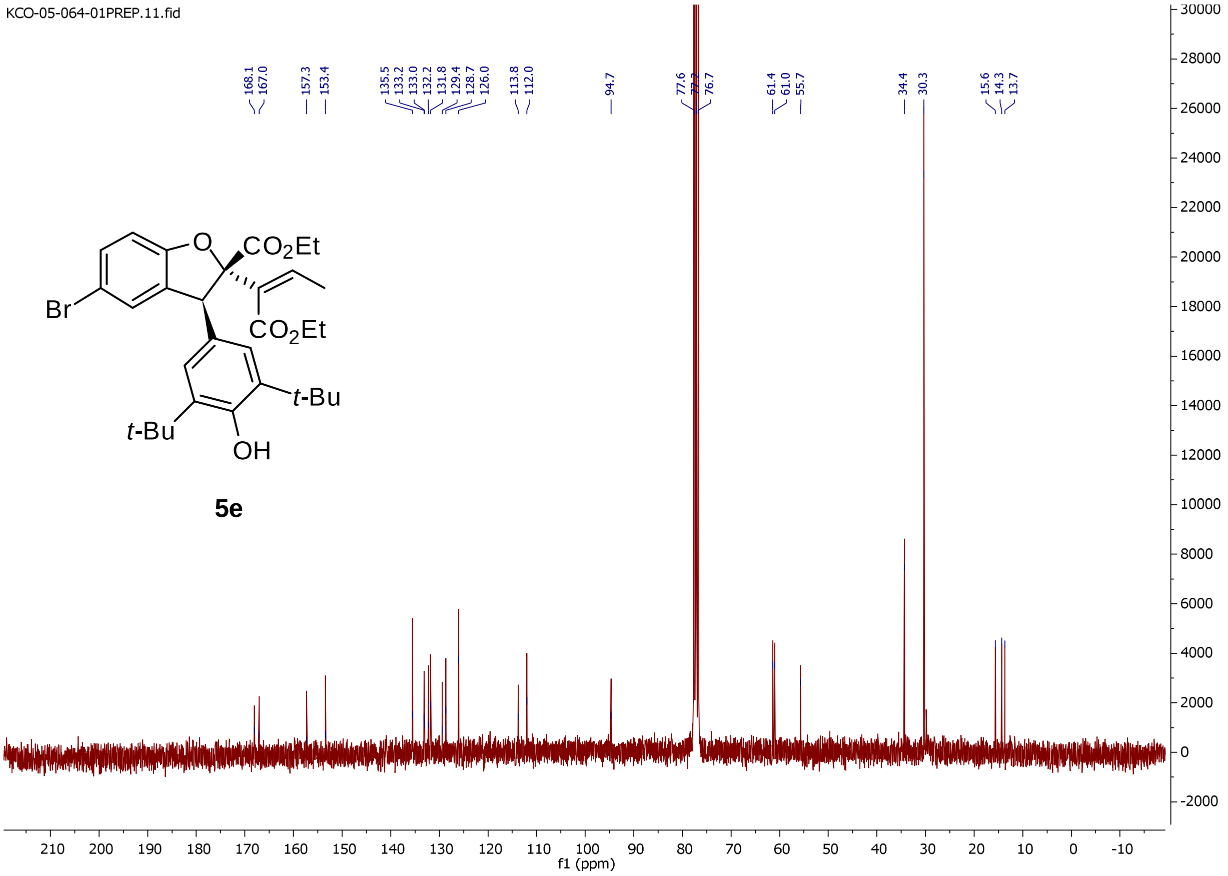
KCO-05-064-01PREP.11.fid
CO2Et
Br
CO2Et
t-Bu
t-Bu
OH
5e
f1 (ppm)

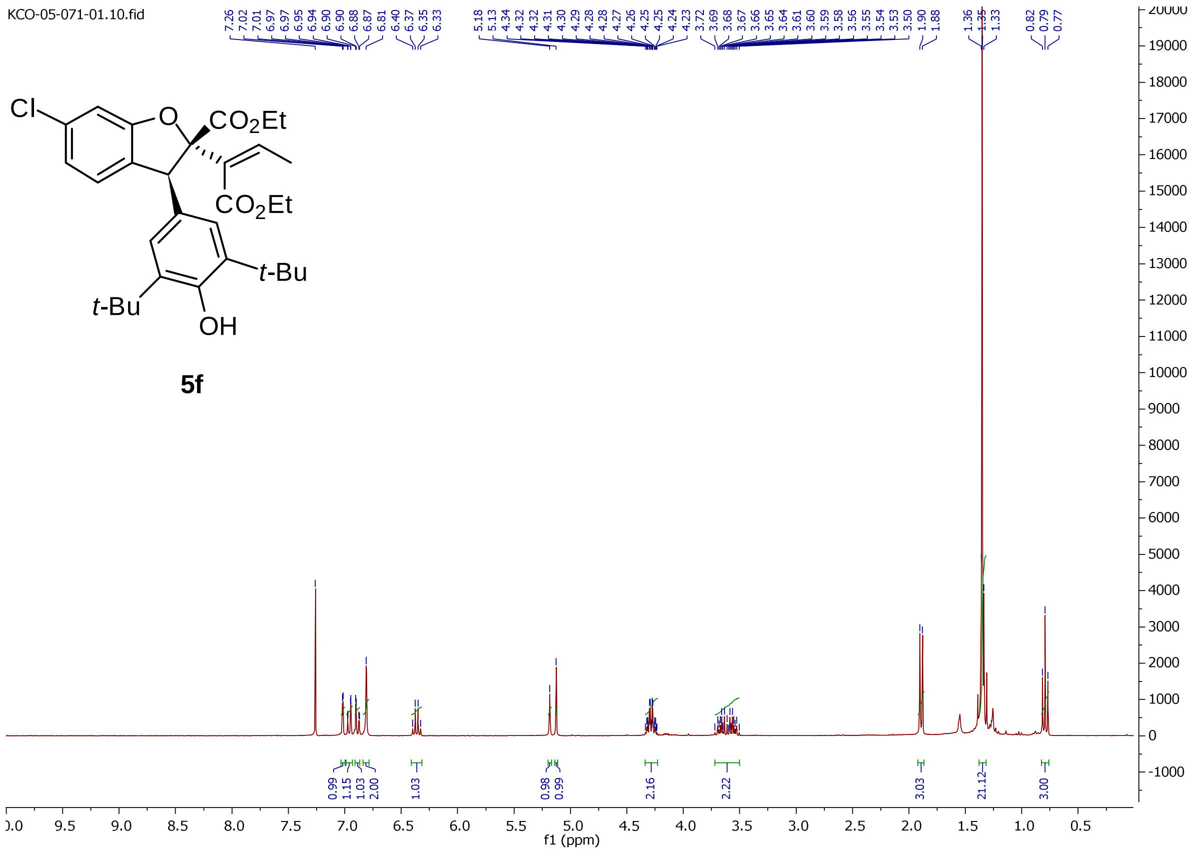
KCO-05-071-01.10.fid
7.26 7.02 7.01 6.97 6.95 6.94 6.90 6.90 6.88 6.87 6.81 6.40 6.37 6.35 6.33
5.18 5.13 4.34 4.32 4.32 4.31 4.30 4.29 4.28 4.28 4.27 4.26 4.25 4.25 4.24 4.23 3.72 3.69 3.68 3.67 3.66 3.65 3.64 3.61 3.60 3.59 3.58 3.56 3.55 3.54 3.53 3.50 1.90 1.88
1.36 1.35 1.33 0.82 0.79 0.77
Cl
O
CO2Et
CO2Et
t-Bu
t-Bu
OH
5f
0.99 1.15 1.03 2.00 1.03 0.98 0.99 2.16 2.22 3.03 21.12 3.00
f1 (ppm)

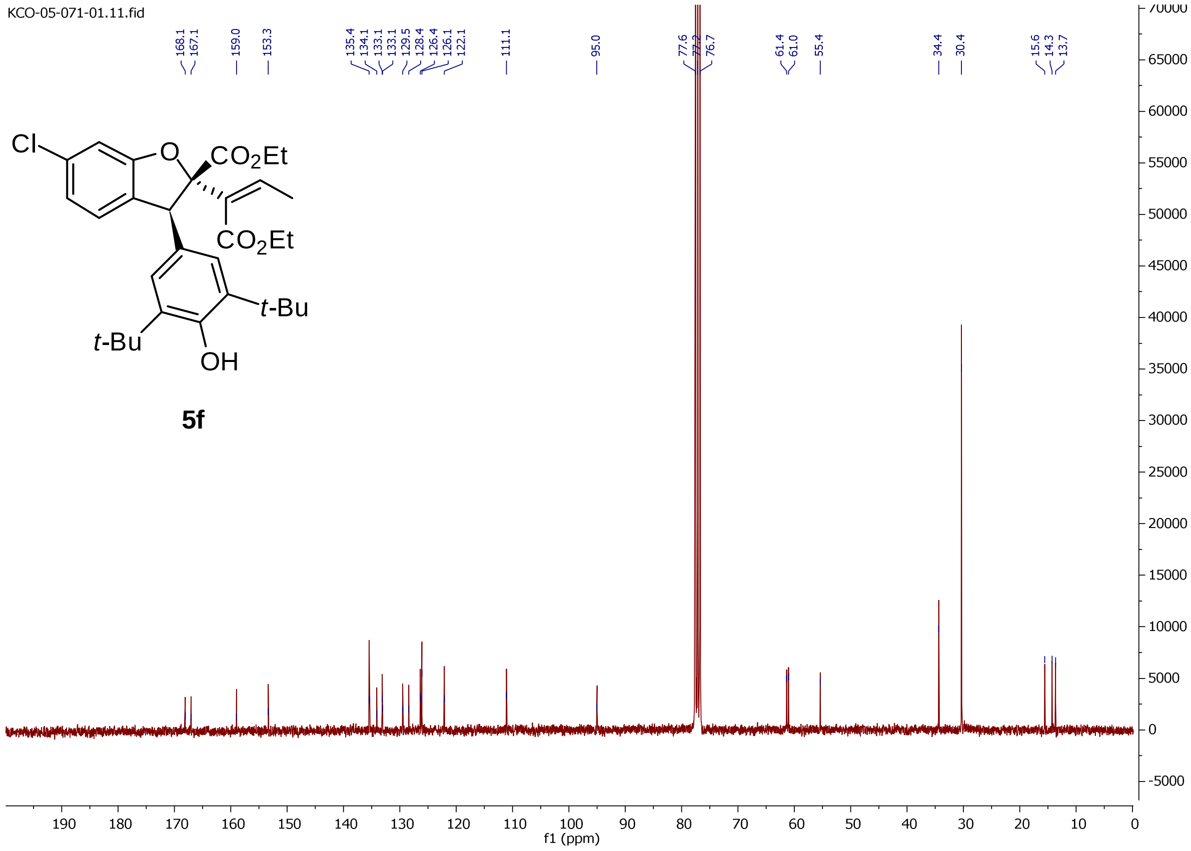
KCO-05-071-01.11.fid
168.1 167.1 159.0 153.3 135.4 134.1 133.1 133.1 129.5 128.4 126.4 126.1 122.1 111.1 95.0 77.6 77.2 76.7 61.4 61.0 55.4 34.4 30.4 15.6 14.3 13.7
Cl
O
CO2Et
CO2Et
t-Bu
t-Bu
OH
5f
f1 (ppm)

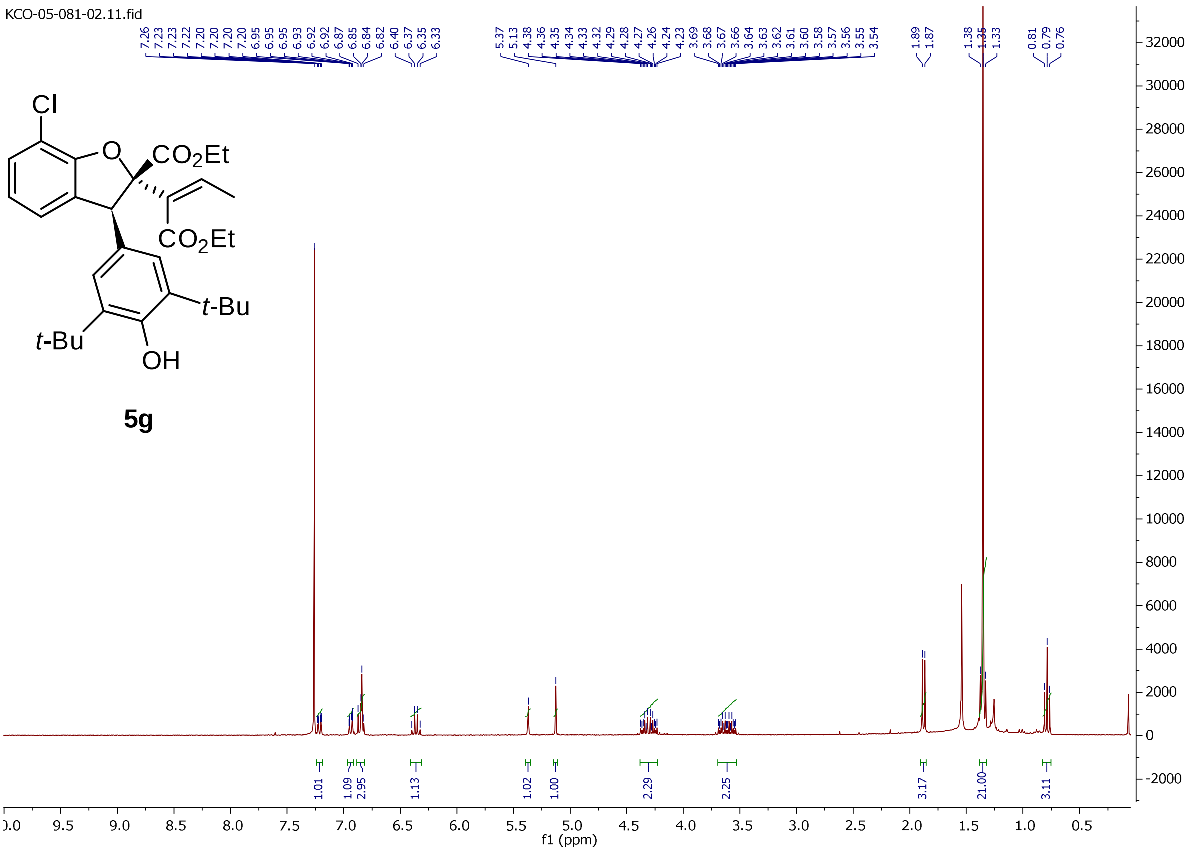
KCO-05-081-02.11.fid
7.26 7.23 7.23 7.22 7.20 7.20 7.20 7.20 6.95 6.95 6.95 6.93 6.92 6.92 6.87 6.85 6.84 6.82 6.40 6.37 6.35 6.33
5.37 5.13 4.38 4.36 4.35 4.34 4.33 4.32 4.29 4.28 4.27 4.26 4.24 4.23 3.69 3.68 3.67 3.66 3.64 3.63 3.62 3.61 3.60 3.58 3.57 3.56 3.55 3.54
1.89 1.87 1.38 1.35 1.33 0.81 0.79 0.76
Cl
O
CO2Et
CO2Et
t-Bu
t-Bu
OH
5g
1.01 1.09 2.95 1.13 1.02 1.00 2.29 2.25 3.17 21.00 3.11
f1 (ppm)

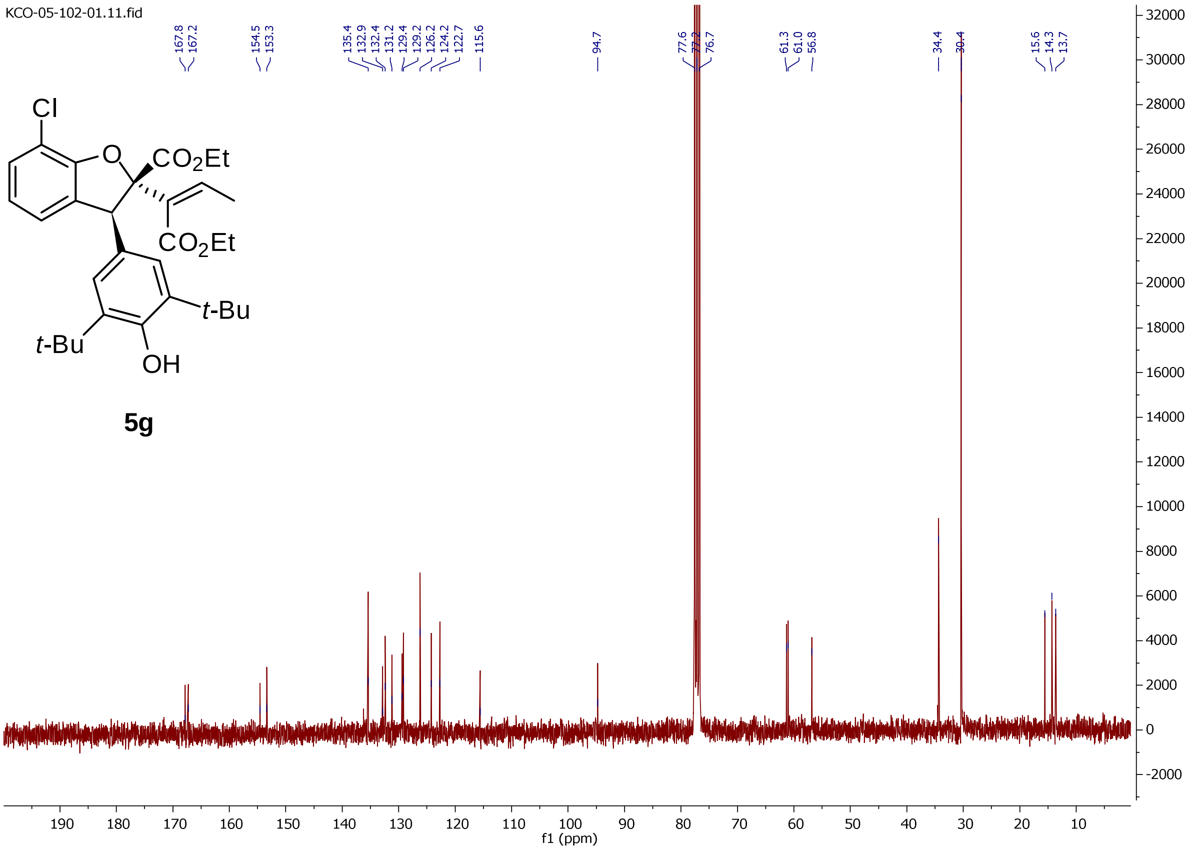
KCO-05-102-01.11.fid
167.8 167.2 154.5 153.3 135.4 132.9 132.4 131.2 129.4 129.2 126.2 124.2 122.7 115.6 94.7 77.6 77.2 76.7 61.3 61.0 56.8 34.4 30.4 15.6 14.3 13.7
Cl
O
CO2Et
CO2Et
t-Bu
t-Bu
OH
5g
f1 (ppm)

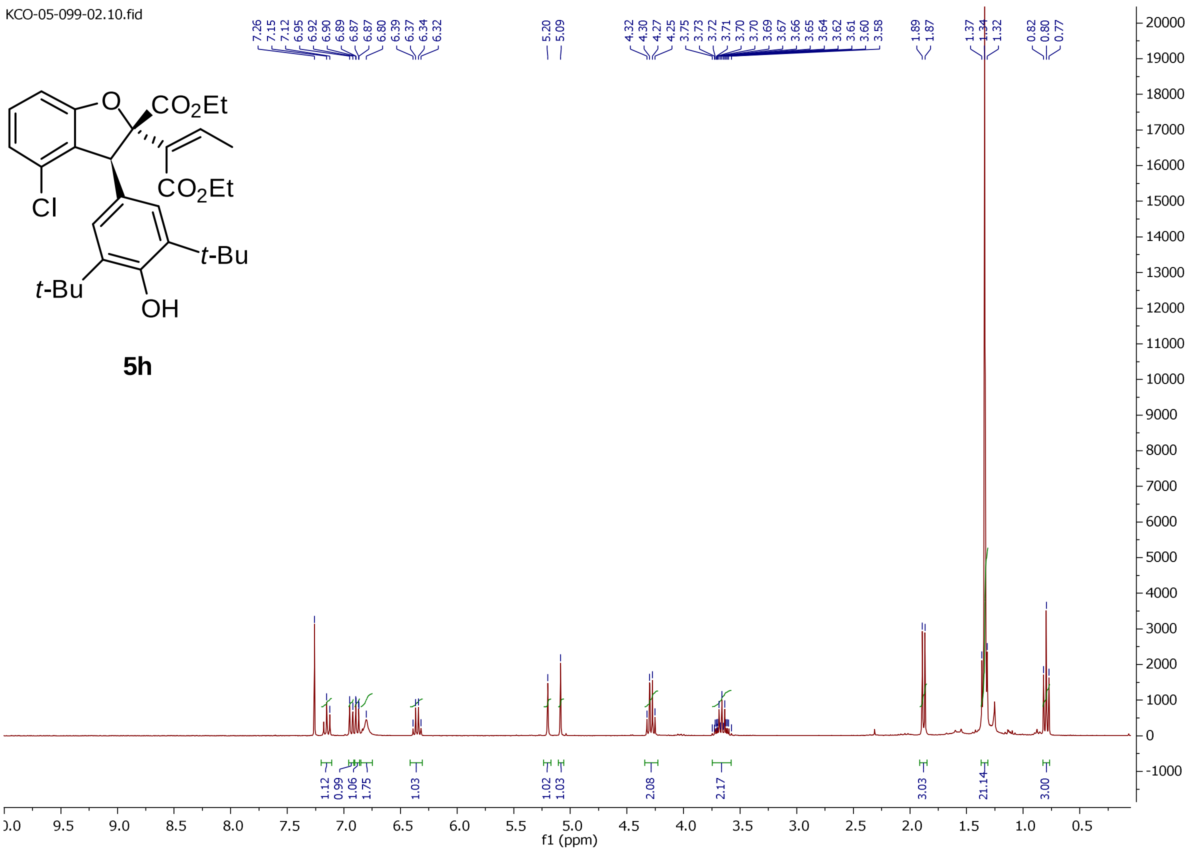
KCO-05-099-02.10.fid
5h
f1 (ppm)

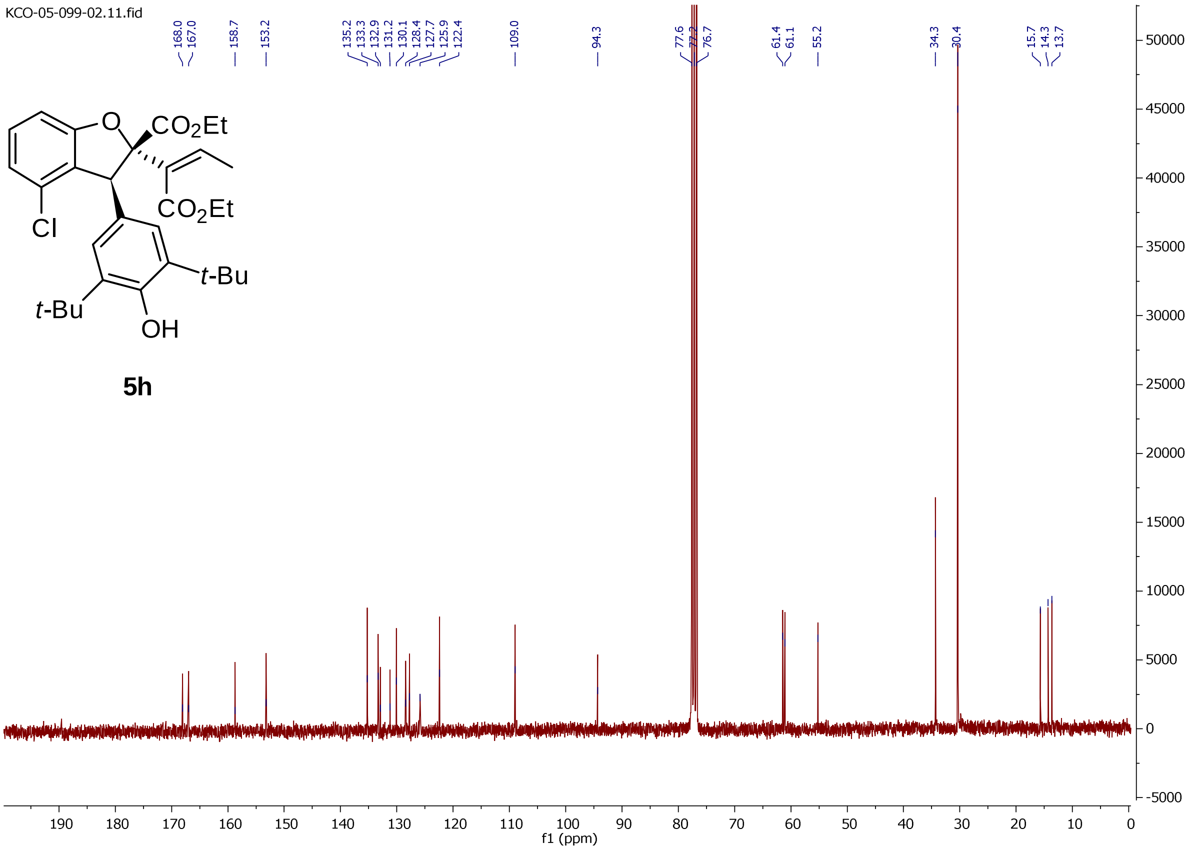
KCO-05-099-02.11.fid
5h
f1 (ppm)

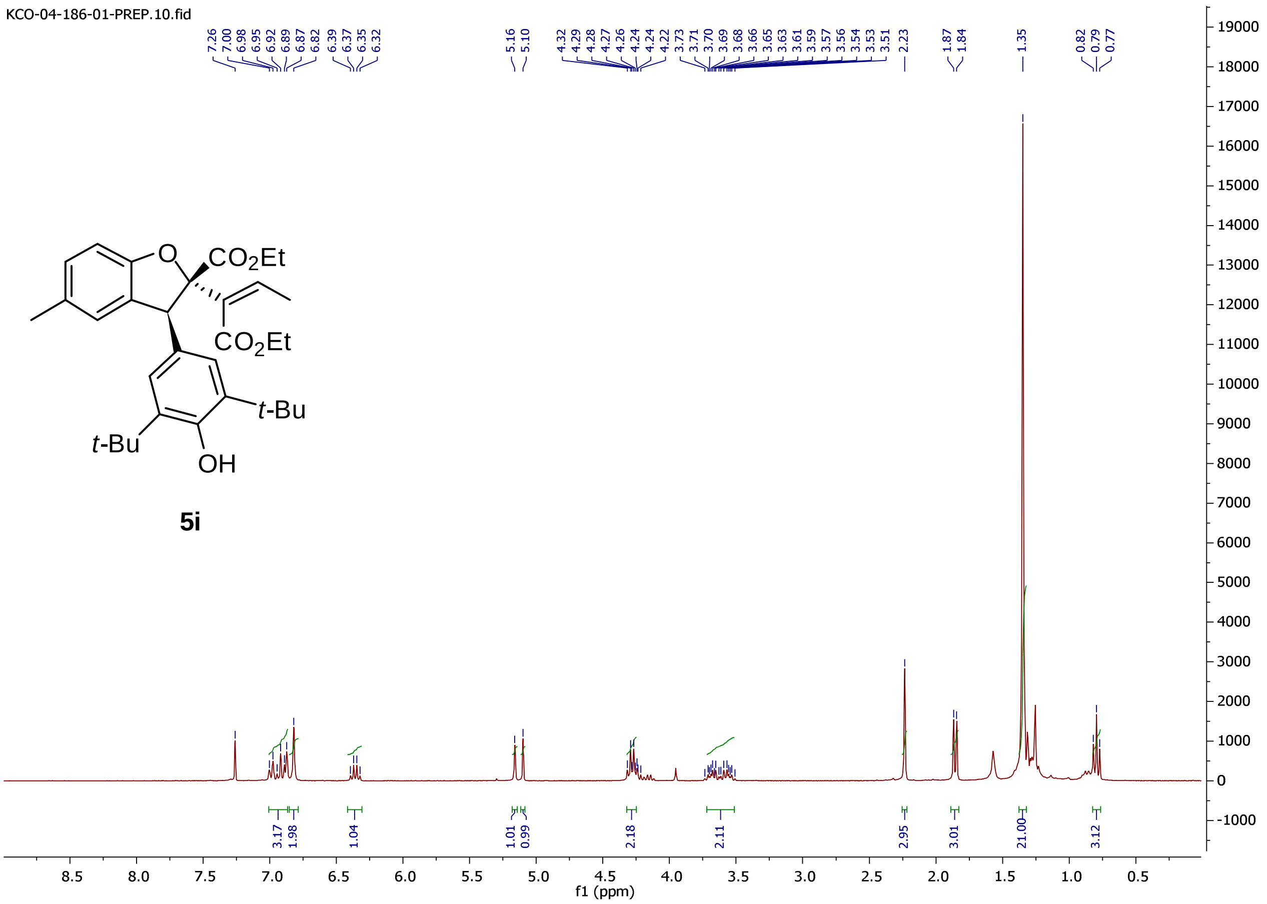
KCO-04-186-01-PREP.10.fid
7.26 7.00 6.98 6.95 6.92 6.89 6.87 6.82 6.39 6.37 6.35 6.32
5.16 5.10
4.32 4.29 4.28 4.27 4.26 4.24 4.24 4.22 3.73 3.71 3.70 3.69 3.68 3.66 3.65 3.63 3.61 3.59 3.57 3.56 3.54 3.53 3.51
2.23
1.87 1.84
1.35
0.82 0.79 0.77
CO2Et
CO2Et
O
t-Bu
t-Bu
OH
5i
3.17 1.98 1.04 1.01 0.99 2.18 2.11 2.95 3.01 21.00 3.12
f1 (ppm)

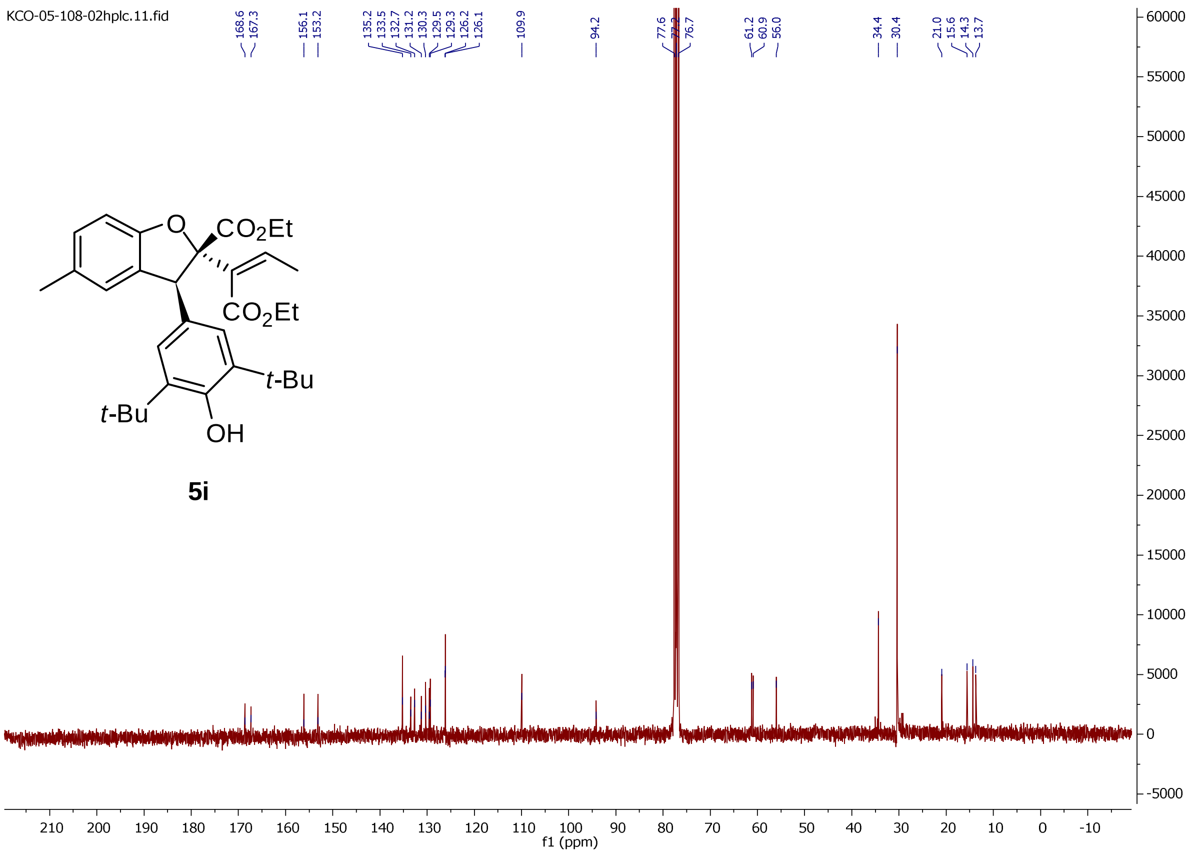
KCO-05-108-02hplc.11.fid
168.6 167.3 156.1 153.2 135.2 133.5 132.7 131.2 130.3 129.5 129.3 126.2 126.1 109.9 94.2 77.6 76.7 61.2 60.9 56.0 34.4 30.4 21.0 15.6 14.3 13.7
CO2Et
CO2Et
O
t-Bu
t-Bu
OH
5i
f1 (ppm)

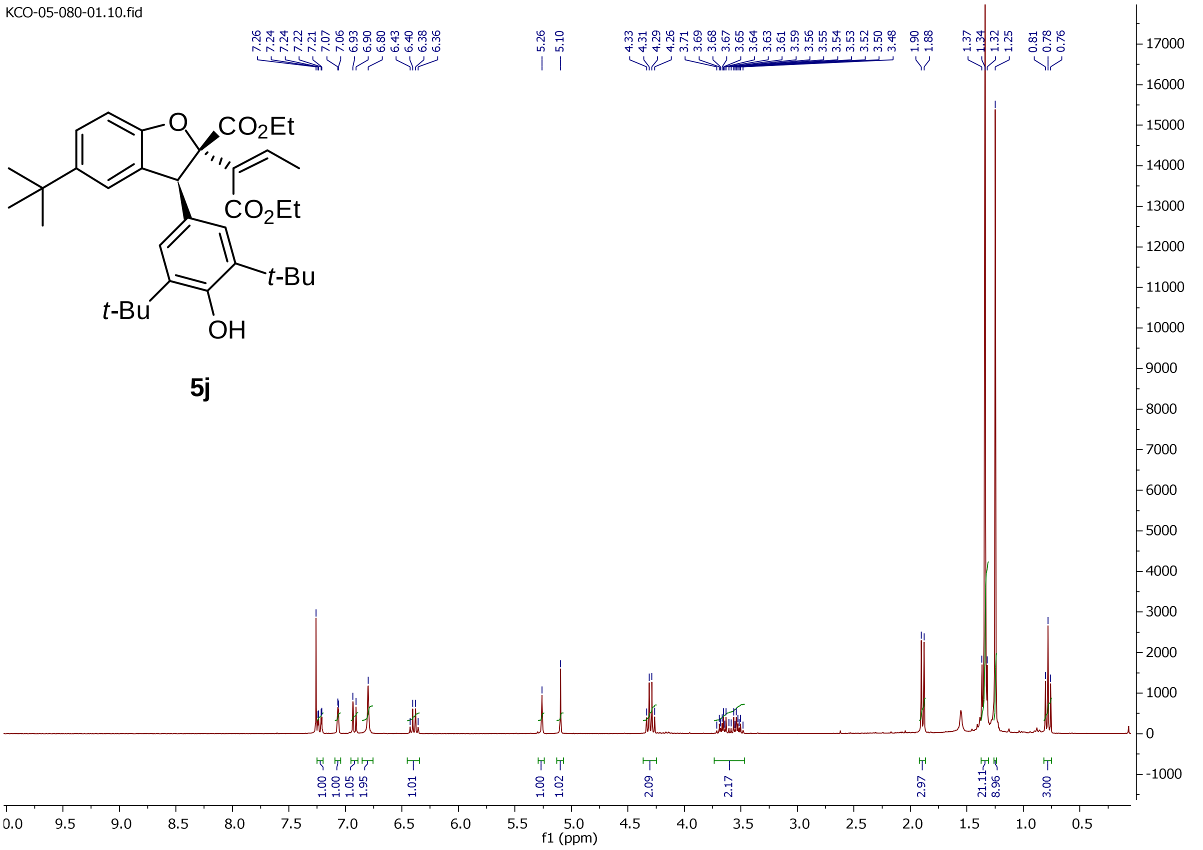
KCO-05-080-01.10.fid
CO2Et
CO2Et
t-Bu
t-Bu
OH
5j
f1 (ppm)

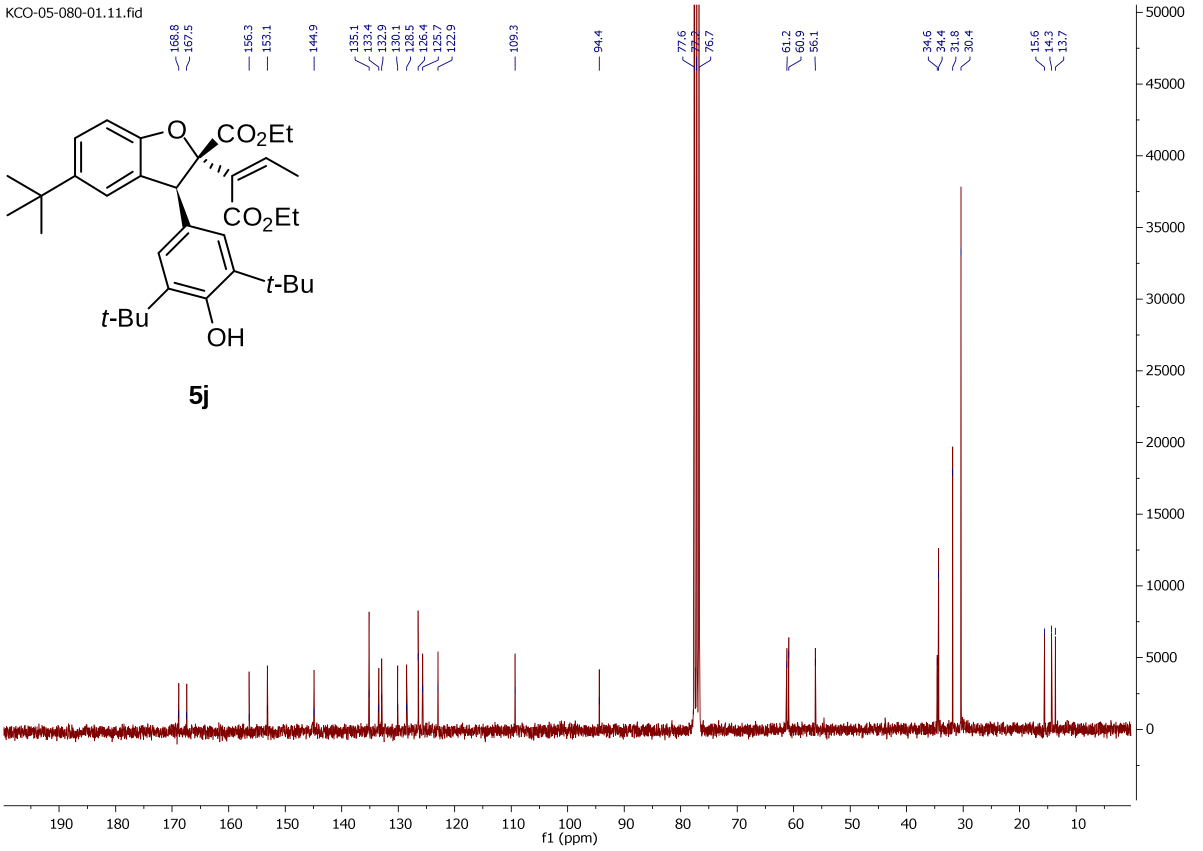
KCO-05-080-01.11.fid
CO2Et
CO2Et
t-Bu
t-Bu
OH
5j
f1 (ppm)

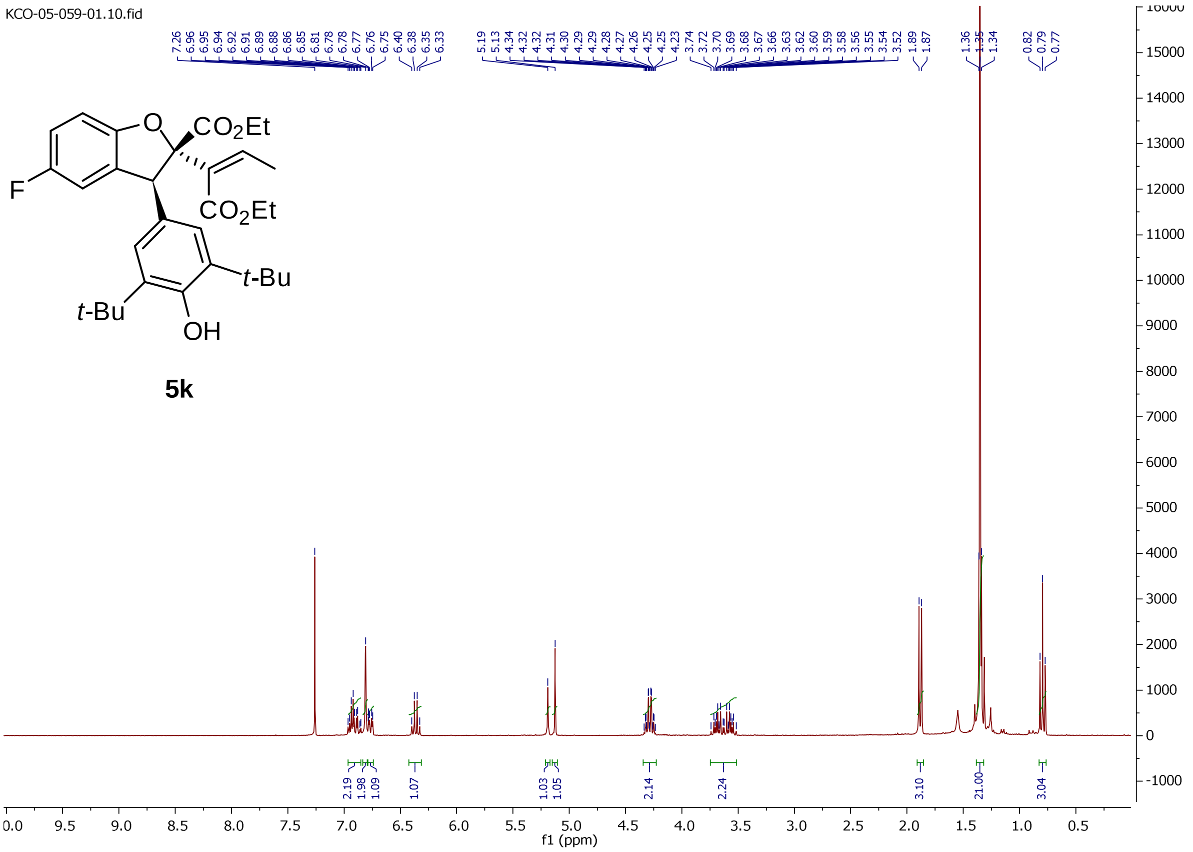

KCO-05-059-01.10.fid
7.26
6.96
6.95
6.94
6.92
6.91
6.89
6.88
6.86
6.85
6.81
6.78
6.77
6.76
6.75
6.40
6.38
6.35
6.33
5.19
5.13
4.34
4.32
4.32
4.31
4.30
4.29
4.29
4.28
4.27
4.26
4.25
4.25
4.23
3.74
3.72
3.70
3.69
3.68
3.67
3.66
3.63
3.62
3.60
3.59
3.58
3.56
3.55
3.54
3.52
1.89
1.87
1.36
1.35
1.34
0.82
0.79
0.77
CO2Et
CO2Et
F
t-Bu
t-Bu
OH
5k
2.19
1.98
1.09
1.07
1.03
1.05
2.14
2.24
3.10
21.00
3.04
f1 (ppm)

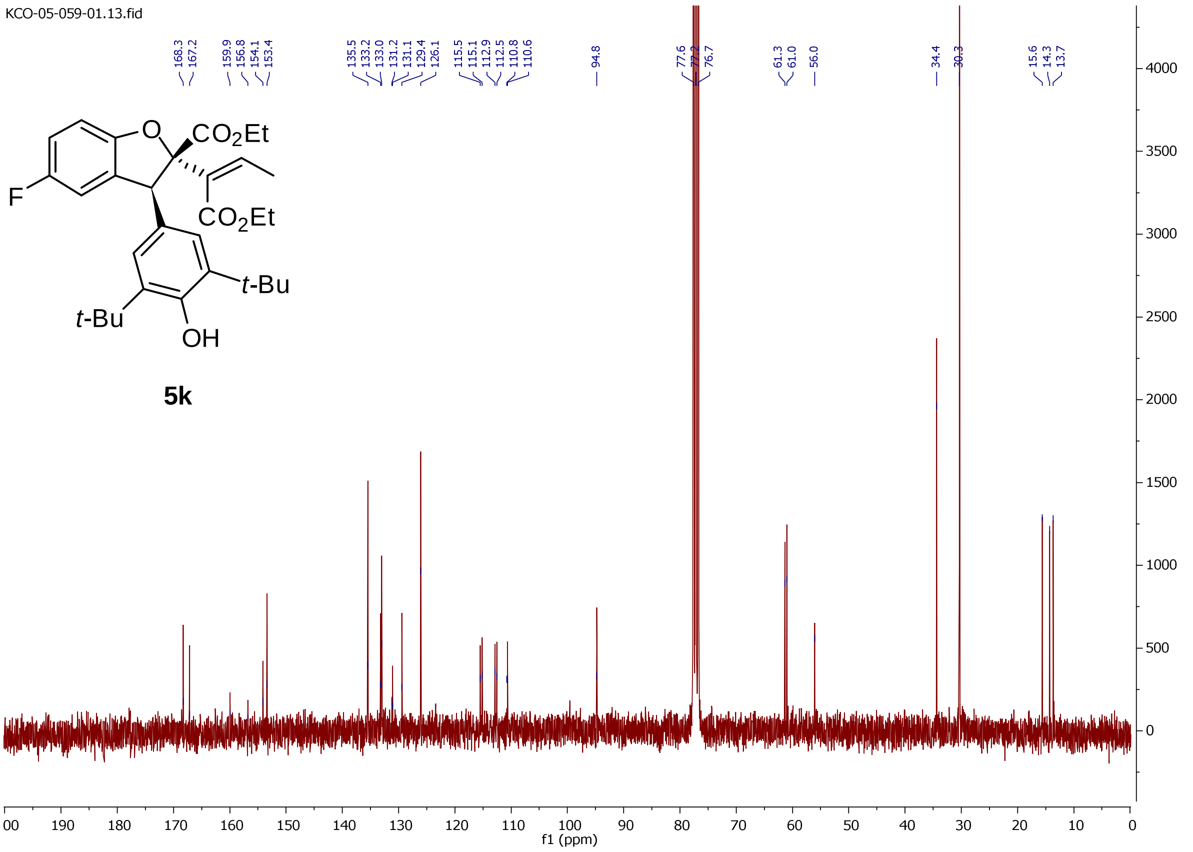

KCO-05-059-01.13.fid
168.3
167.2
159.9
156.8
154.1
153.4
135.5
133.2
133.0
131.2
131.1
129.4
126.1
115.5
115.1
112.9
112.5
110.8
110.6
94.8
77.6
77.2
76.7
61.3
61.0
56.0
34.4
30.3
15.6
14.3
13.7
CO2Et
CO2Et
F
t-Bu
t-Bu
OH
5k
f1 (ppm)

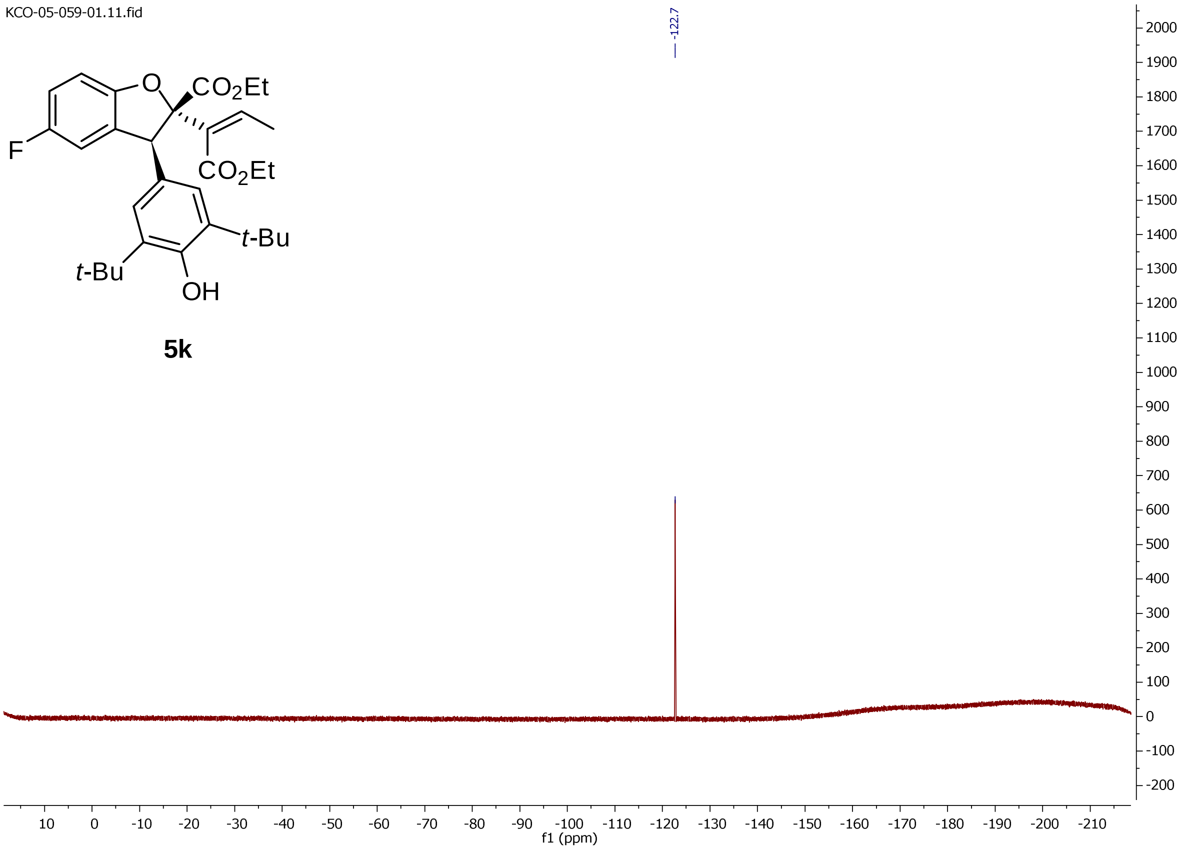
KCO-05-059-01.11.fid
-122.7
CO2Et
CO2Et
F
t-Bu
t-Bu
OH
5k
f1 (ppm)

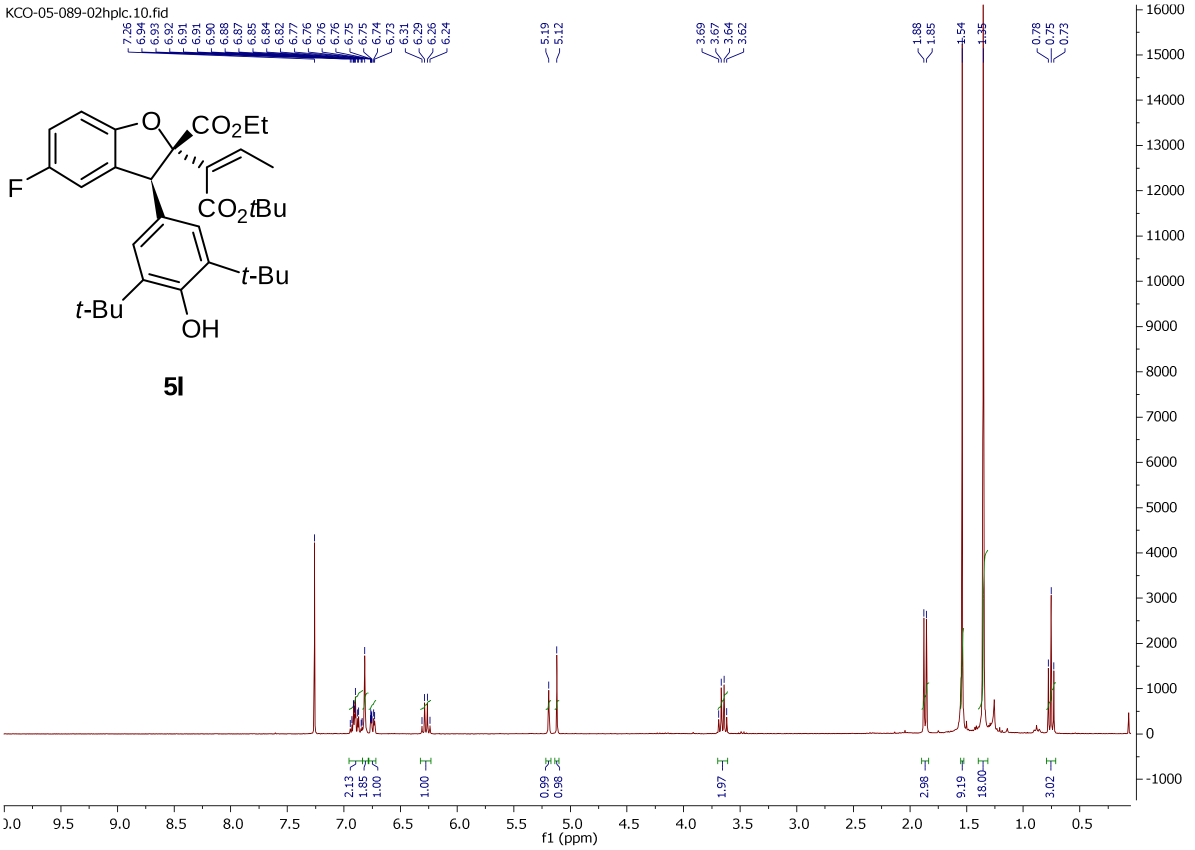
KCO-05-089-02hplc.10.fid
7.26 6.94 6.93 6.92 6.91 6.91 6.90 6.88 6.87 6.85 6.84 6.82 6.77 6.76 6.76 6.76 6.75 6.75 6.74 6.73 6.31 6.29 6.26 6.24
5.19 5.12
3.69 3.67 3.64 3.62
1.88 1.85 1.54 1.35 0.78 0.75 0.73
CO2Et
CO2tBu
F
t-Bu
t-Bu
OH
5l
2.13 1.85 1.00 1.00 0.99 0.98 1.97 2.98 9.19 18.00 3.02
f1 (ppm)

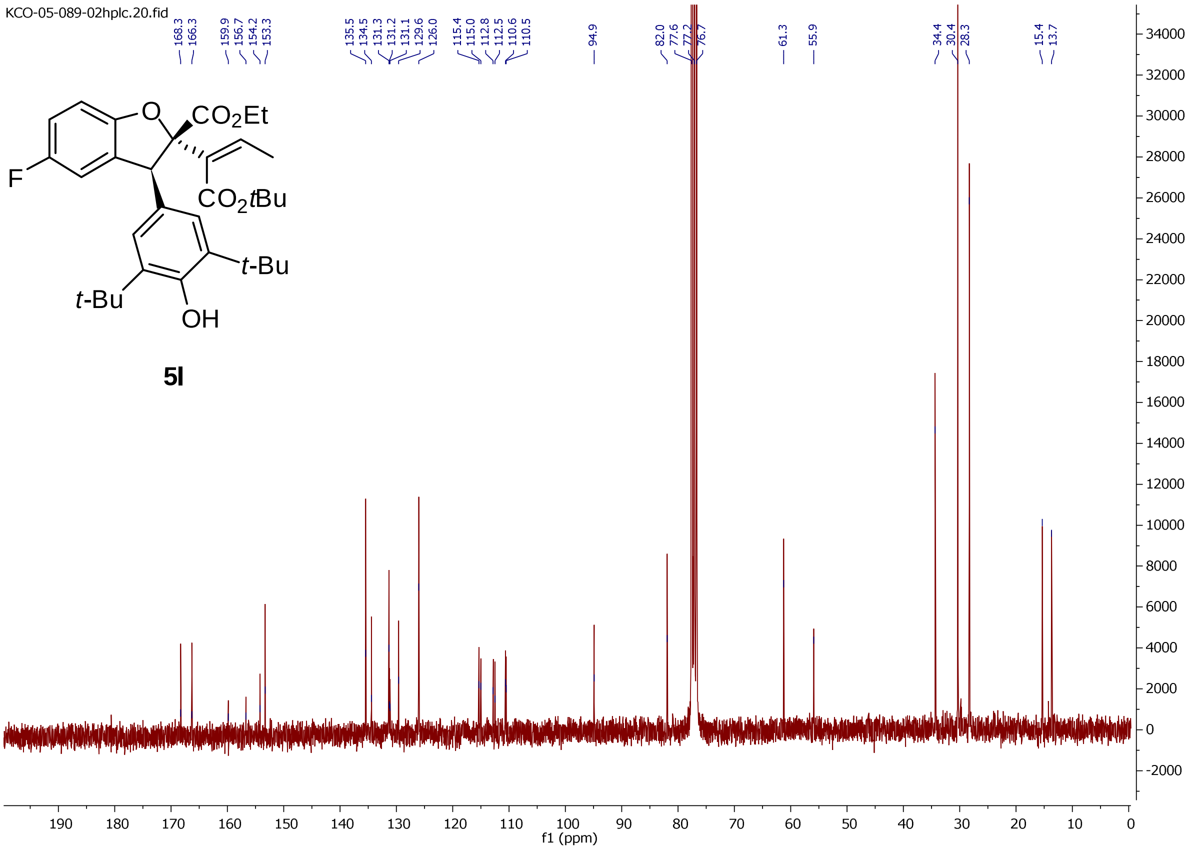
KCO-05-089-02hplc.20.fid
168.3
166.3
159.9
156.7
154.2
153.3
135.5
134.5
131.3
131.2
131.1
129.6
126.0
115.4
115.0
112.8
112.5
110.6
110.5
94.9
82.0
77.6
77.2
76.7
61.3
55.9
34.4
30.4
28.3
15.4
13.7
CO2Et
F
CO2tBu
t-Bu
t-Bu
OH
5l
f1 (ppm)

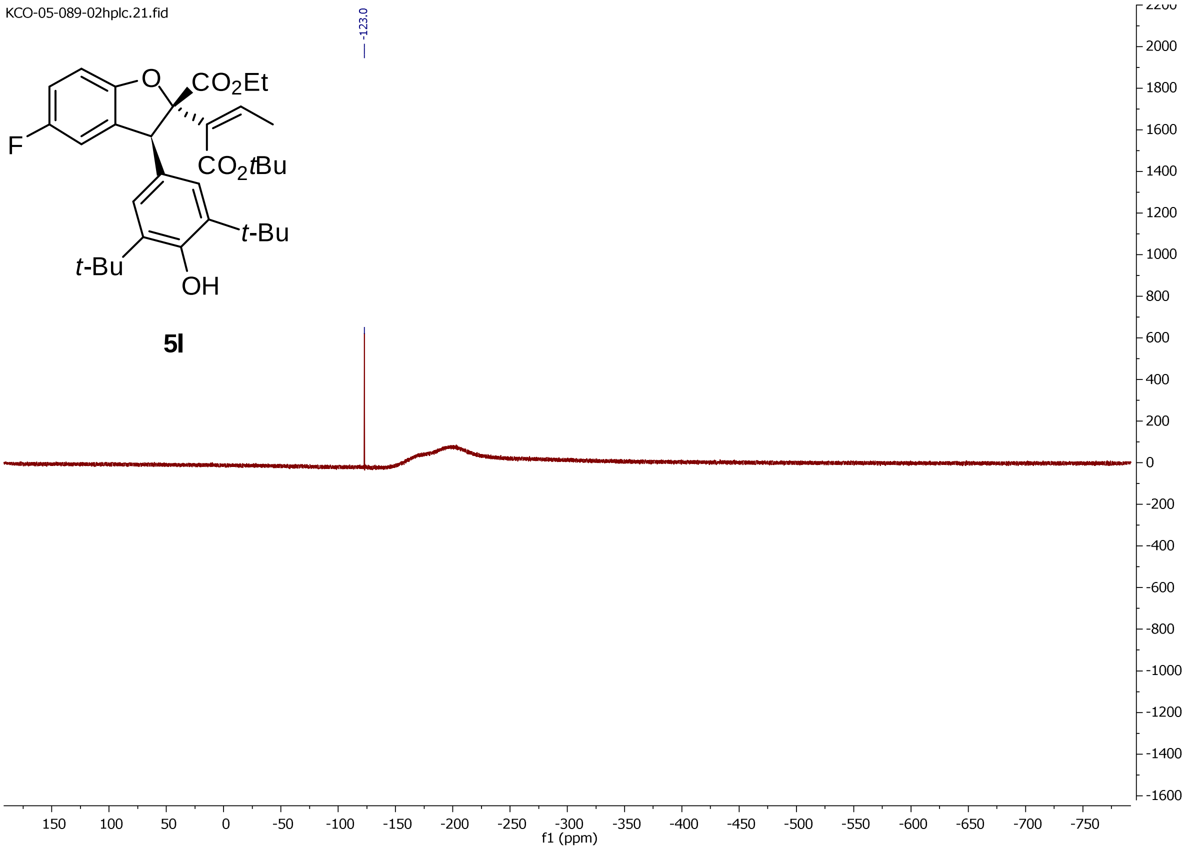
KCO-05-089-02hplc.21.fid
-123.0
CO2Et
F
CO2tBu
t-Bu
t-Bu
OH
5l
f1 (ppm)

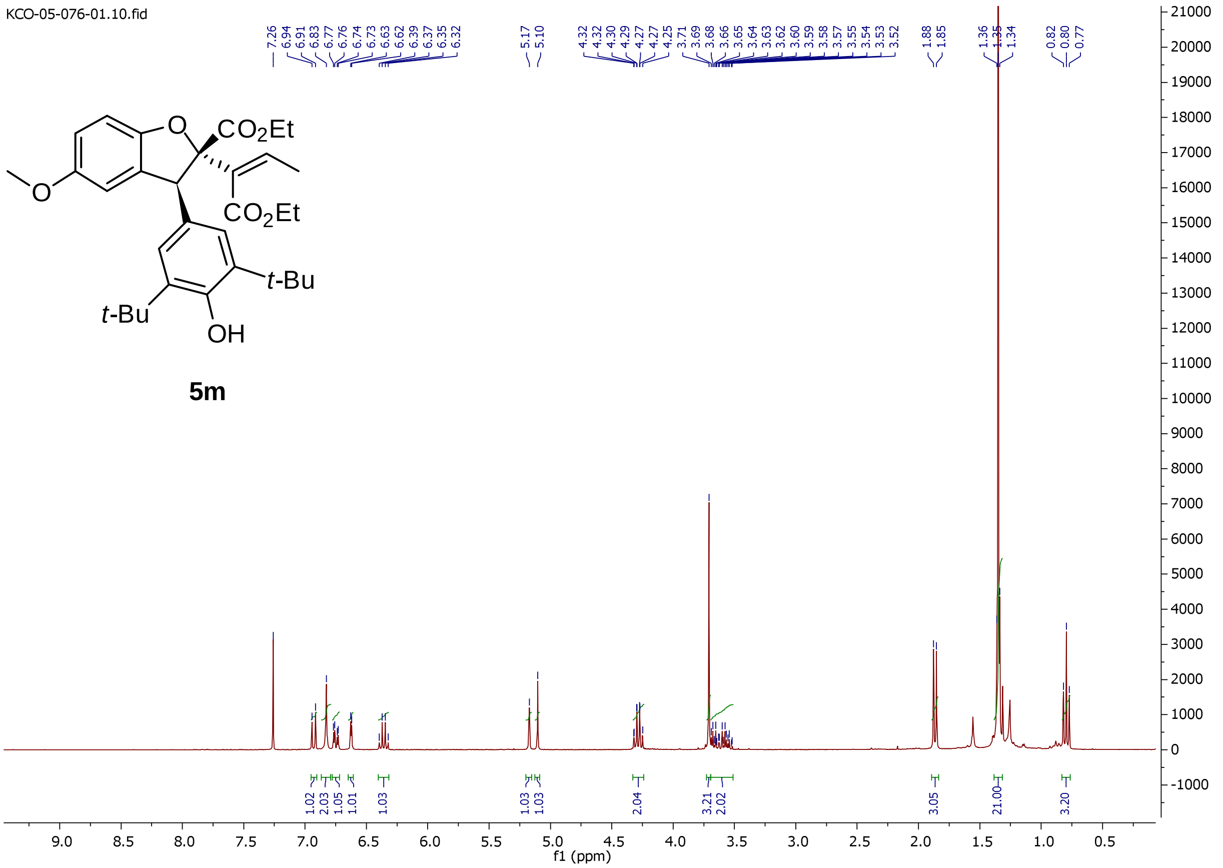
KCO-05-076-01.10.fid
5m
t-Bu
OH
f1 (ppm)

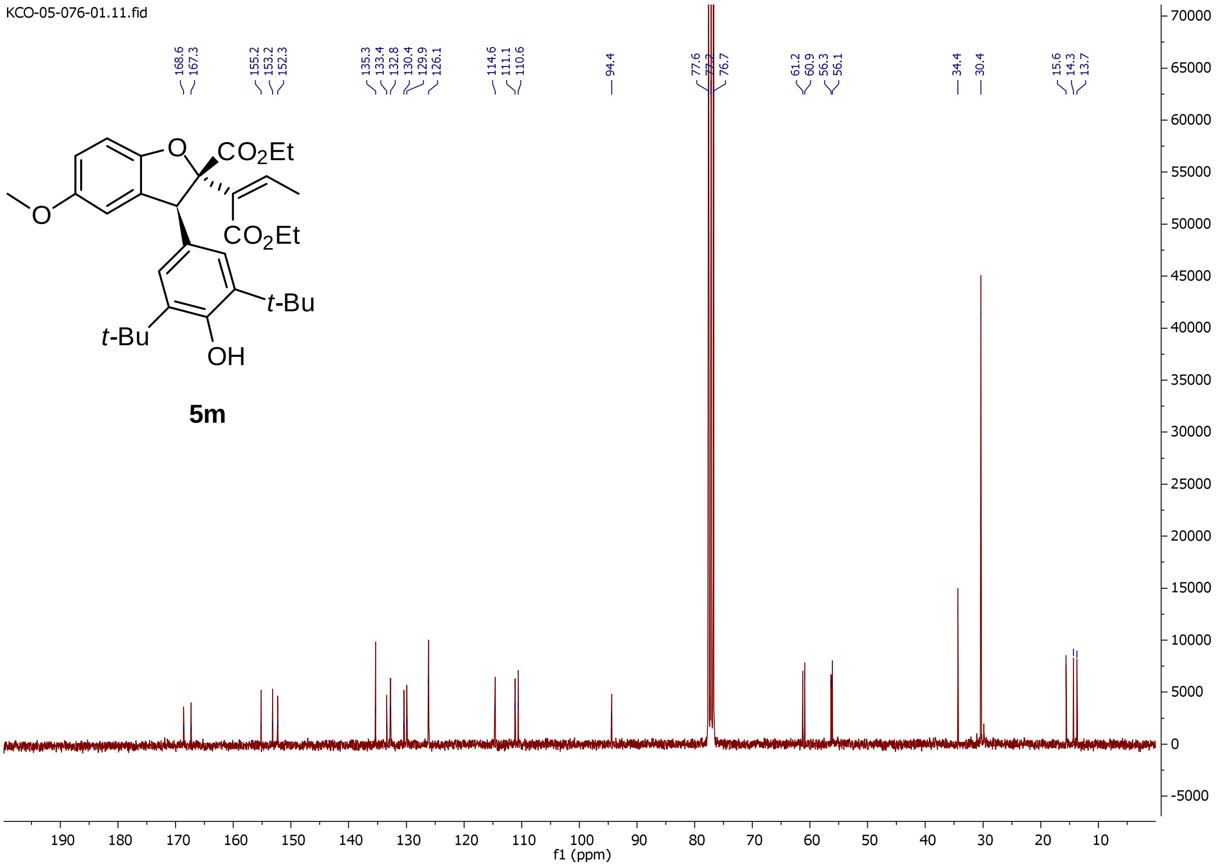
KCO-05-076-01.11.fid
5m
t-Bu
OH
f1 (ppm)

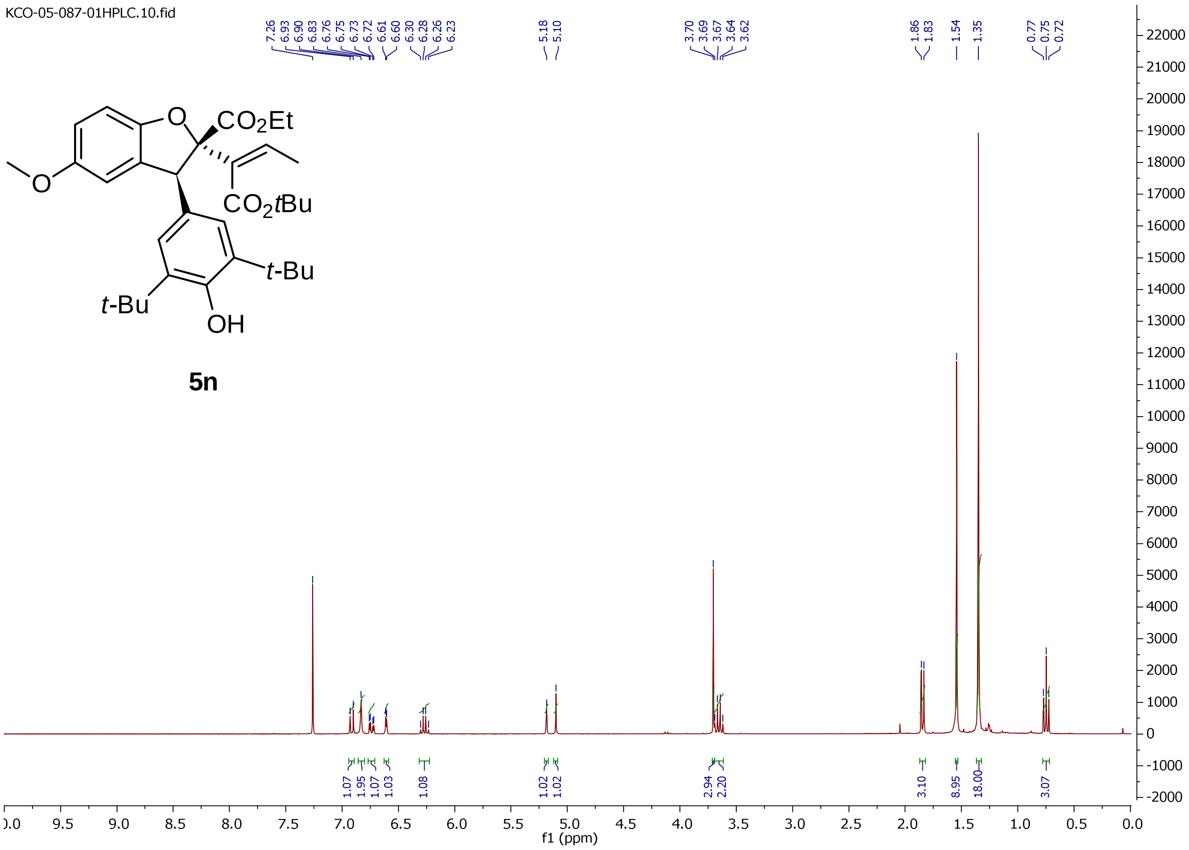
KCO-05-087-01HPLC.10.fid
7.26 6.93 6.90 6.83 6.76 6.75 6.73 6.72 6.61 6.60 6.30 6.28 6.26 6.23
5.18 5.10
3.70 3.69 3.67 3.64 3.62
1.86 1.83 1.54 1.35 0.77 0.75 0.72
CO_2Et
CO_2tBu
t-Bu
t-Bu
OH
O
5n
1.07 1.95 1.07 1.03 1.08 1.02 1.02 2.94 2.20 3.10 8.95 18.00 3.07
f1 (ppm)

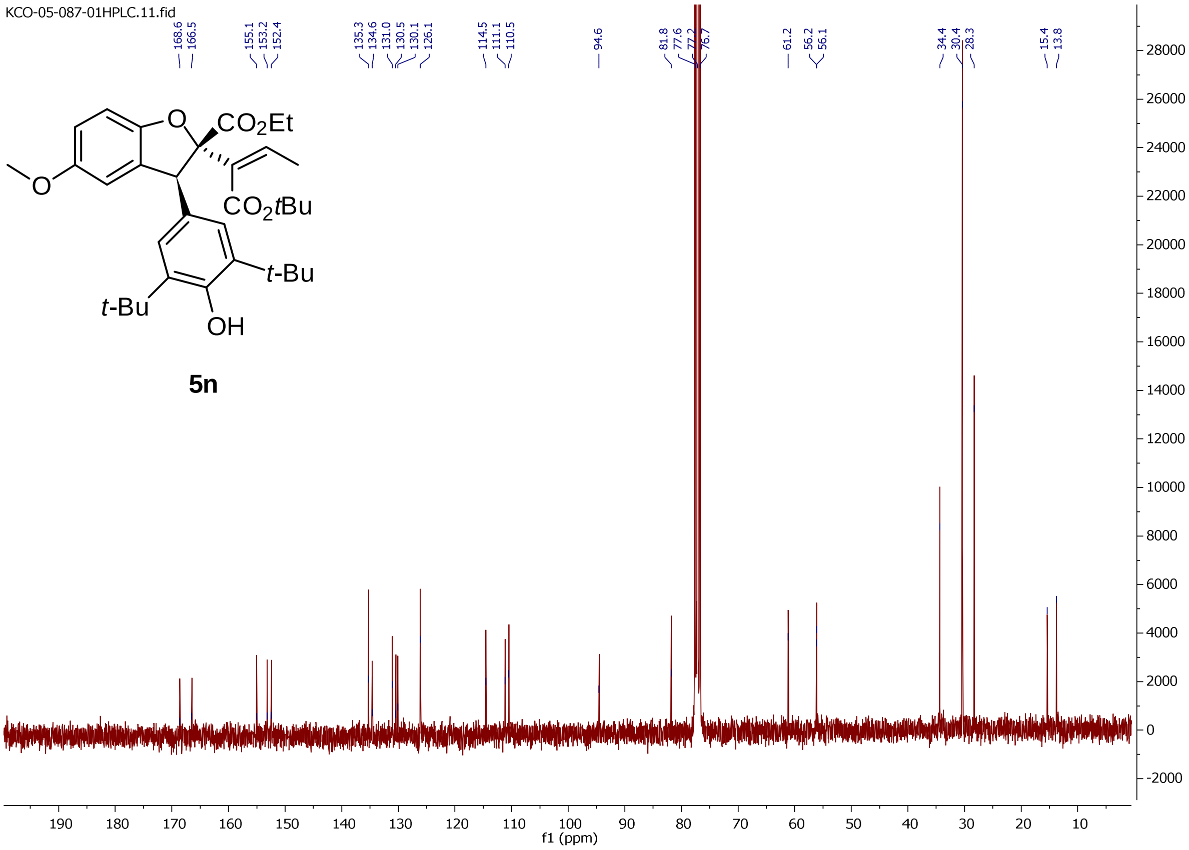
KCO-05-087-01HPLC.11.fid
168.6 166.5 155.1 153.2 152.4 135.3 134.6 131.0 130.5 130.1 126.1 114.5 111.1 110.5 94.6 81.8 77.6 77.2 76.7 61.2 56.2 56.1 34.4 30.4 28.3 15.4 13.8
CO_2Et
CO_2tBu
t-Bu
t-Bu
OH
O
5n
f1 (ppm)

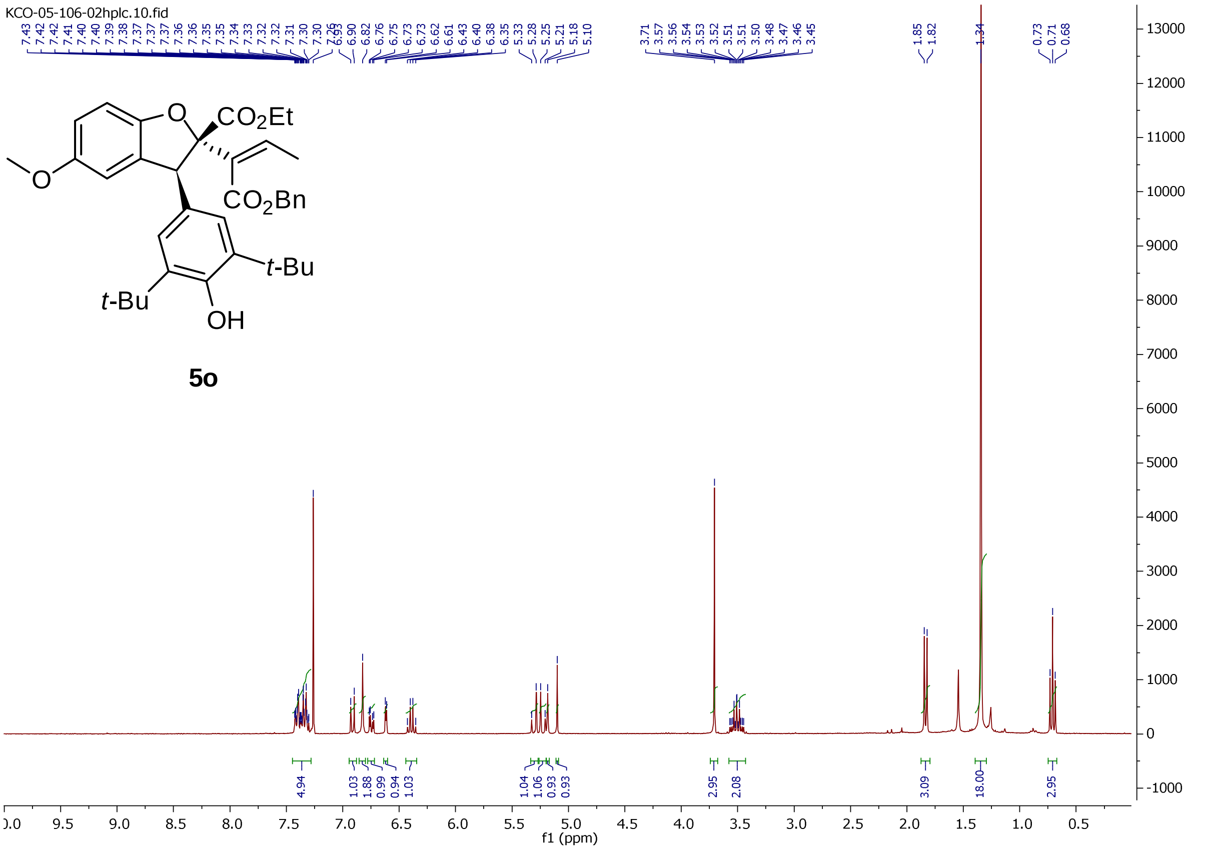

KCO-05-106-02hplc.10.fid
5o
f1 (ppm)

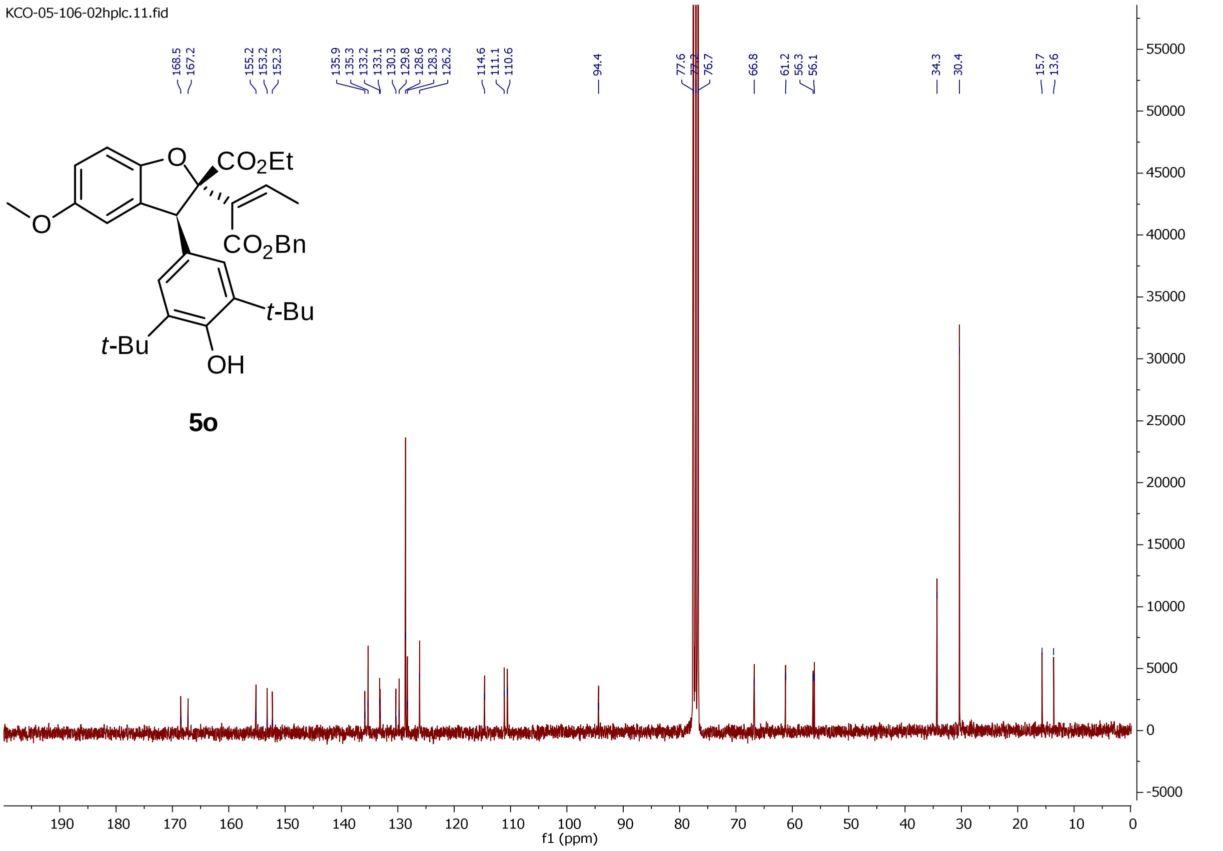

KCO-05-106-02hplc.11.fid
5o
f1 (ppm)

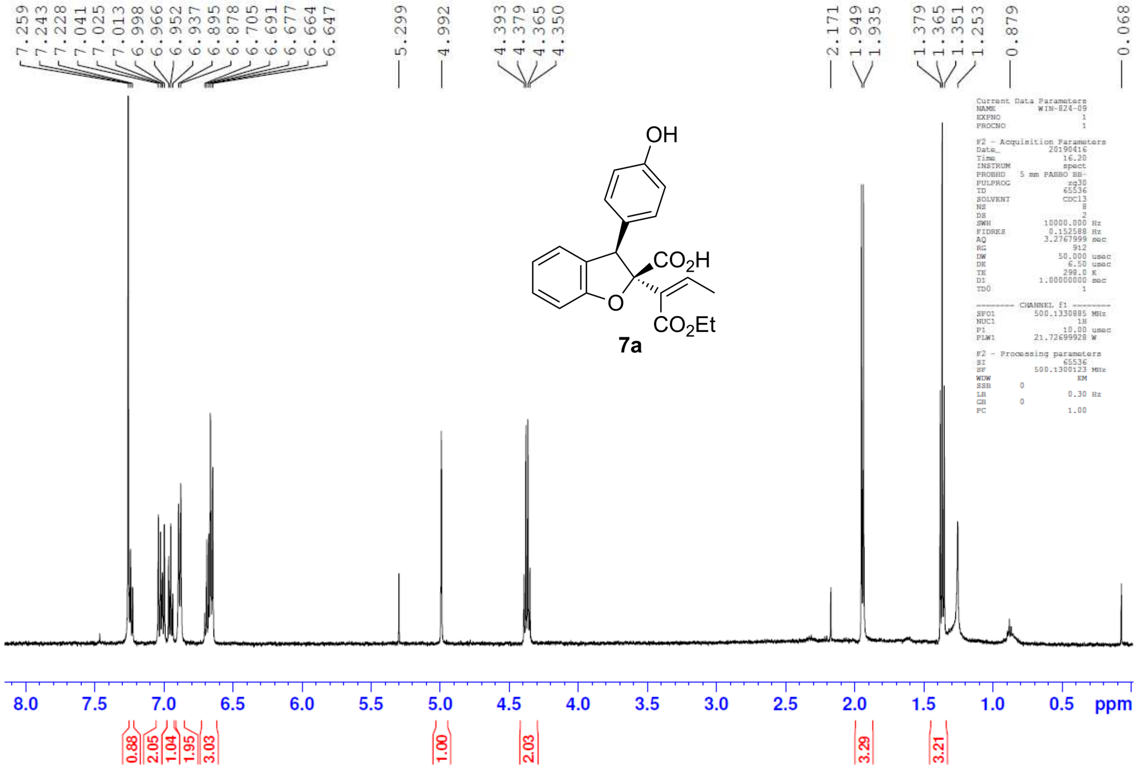
7.259
7.243
7.228
7.041
7.025
7.013
6.998
6.966
6.952
6.937
6.895
6.878
6.705
6.691
6.677
6.664
6.647
5.299
4.992
4.393
4.379
4.365
4.350
2.171
1.949
1.935
1.379
1.365
1.351
1.253
0.879
0.068
OH
CO_2H
O
CO_2Et
7a
8.0 7.5 7.0 6.5 6.0 5.5 5.0 4.5 4.0 3.5 3.0 2.5 2.0 1.5 1.0 0.5 ppm
0.88
2.05
1.04
1.95
3.03
1.00
2.03
3.29
3.21

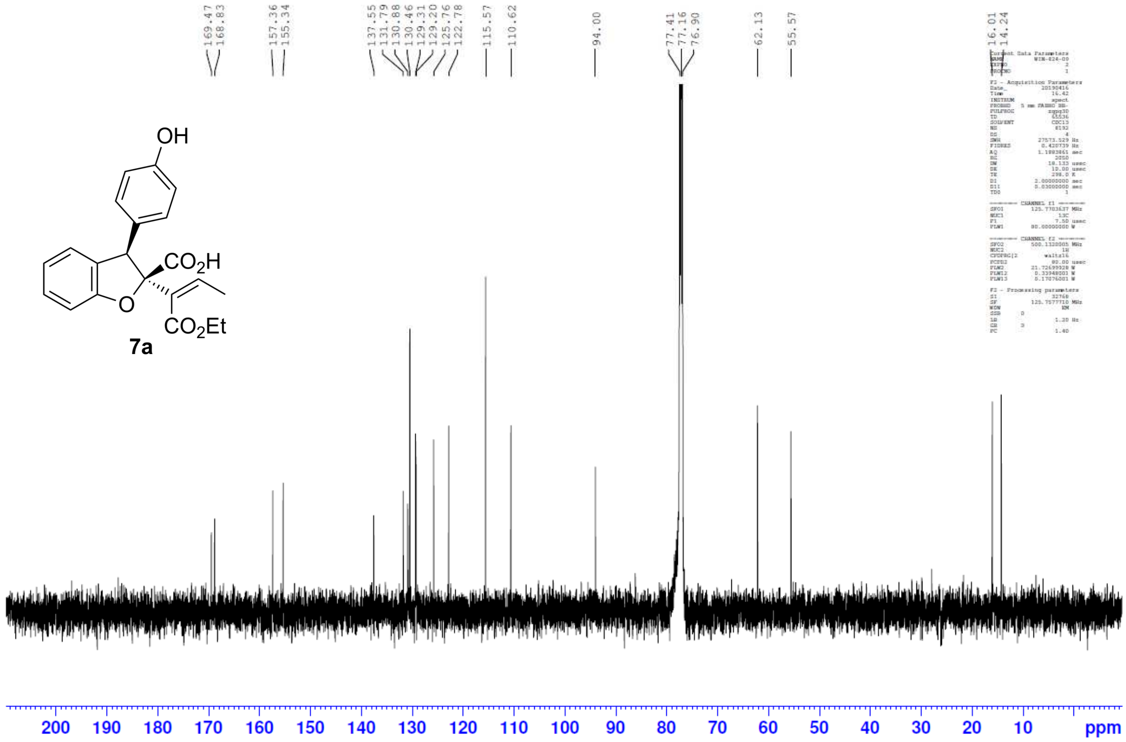
169.47
168.83
157.36
155.34
137.55
131.79
130.88
130.46
129.31
129.20
125.76
122.78
115.57
110.62
94.00
77.41
77.16
76.90
62.13
55.57
16.01
14.24
OH
CO_2H
O
CO_2Et
7a
200 190 180 170 160 150 140 130 120 110 100 90 80 70 60 50 40 30 20 10 ppm

4 Copies of HPLC Chromatograms

Operator:Admin Timebase:U-3000_DAD Sequence:WAS_20180907_ZIE_YMC

Page 1-2
12.3.2019 6:23 PM

2 ZIE-1222-01 95Hexan_5IPA_0,5flow

Sample Name:	**ZIE-1222-01 95Hexan_5IPA_0,5flow**	*Injection Volume:*	**20,0**
Vial Number:	**RC1**	*Channel:*	**UV_VIS_1**
Sample Type:	**unknown**	*Wavelength:*	**220**
Control Program:	**YMC60min_100A_flow0,5**	*Bandwidth:*	**4**
Quantif. Method:	**default**	*Temperature/Column:*	**10**
Recording Time:	**7.9.2018 10:31**	*Flow ml/min:*	**0,500**
Run Time (min):	**17,98**	*Sample Amount:*	**1,0000**

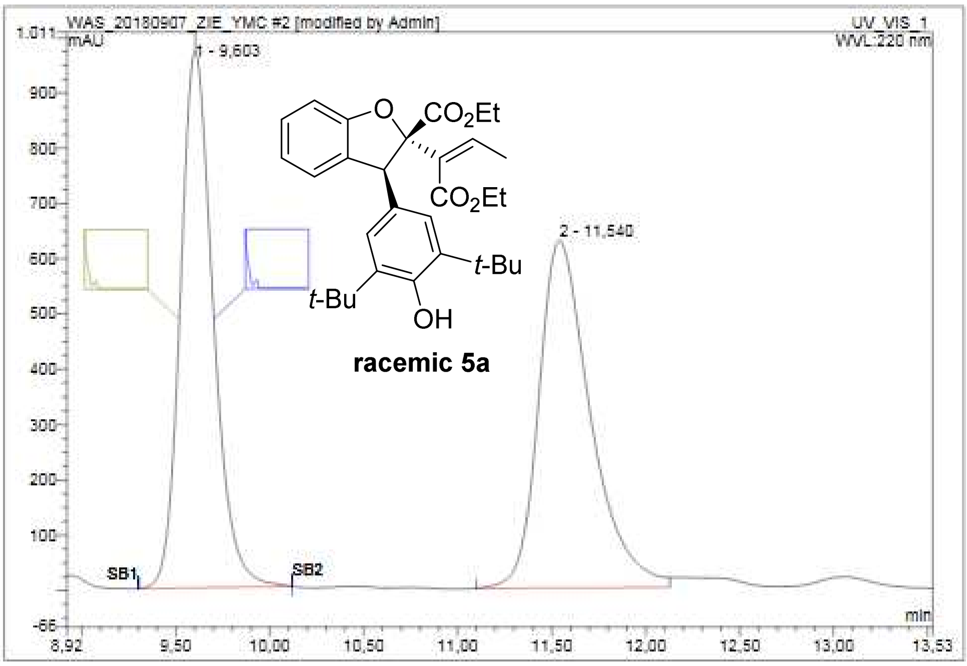

racemic 5a

No.	Ret.Time min	Peak Name	Height mAU	Area mAU*min	Rel.Area %	Amount	Type
1	9,60	n.a.	971,265	197,154	49,63	n.a.	BMB*
2	11,54	n.a.	628,077	200,122	50,37	n.a.	BM *
**Total:**			1599,341	397,276	100,00	0,000	

default/Integration

Chromeleon (c) Dionex 1996-2006
Version 6.80 SR12 Build 3578 (207169)

7 ZIE-1259-01 95Hexan_5IPA_0,5flow

Sample Name:	**ZIE-1259-01 95Hexan_5IPA_0,5flow**	*Injection Volume:*	**20,0**
Vial Number:	**RA4**	*Channel:*	**UV_VIS_1**
Sample Type:	**unknown**	*Wavelength:*	**220**
Control Program:	**YMC15min_100A_flow0,5**	*Bandwidth:*	**4**
Quantif. Method:	**default**	*Temperature/Column:*	**10**
Recording Time:	**11.9.2018 14:10**	*Flow ml/min:*	**0,500**
Run Time (min):	**15,00**	*Sample Amount:*	**1,0000**

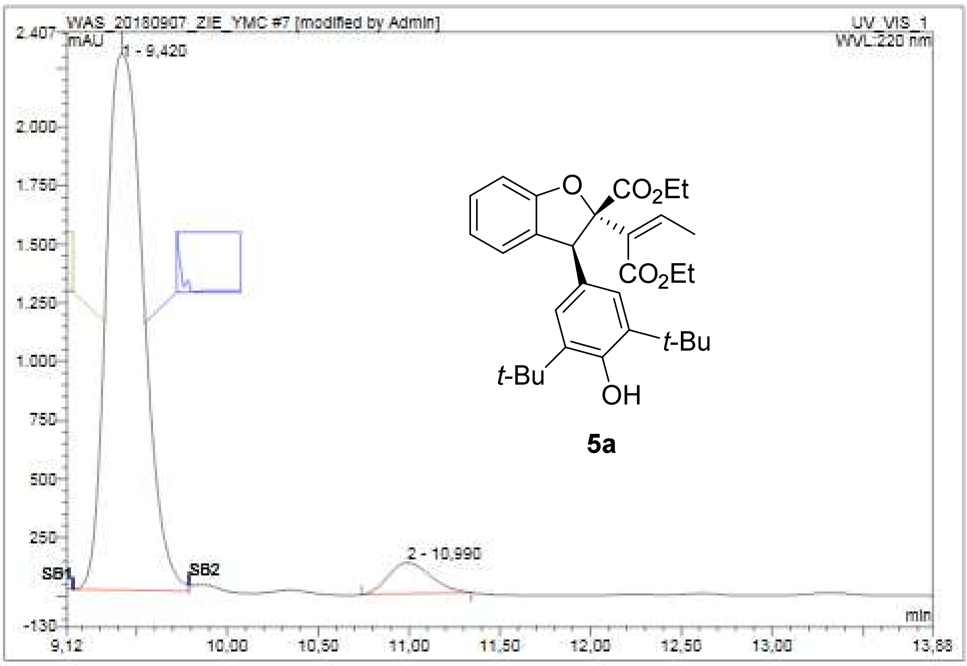

No.	Ret.Time min	Peak Name	Height mAU	Area mAU*min	Rel.Area %	Amount	Type
1	9,42	n.a.	2286,765	546,243	93,85	n.a.	BM *
2	10,99	n.a.	132,449	35,766	6,15	n.a.	BMB*
Total:			2419,213	582,009	100,00	0,000	

6 KCO-05-107-01hplc 90Hexan_10IPA0,5flow

Sample Name:	**KCO-05-107-01hplc 90Hexan_10IPA0,5flow**	*Injection Volume:*	**20,0**
Vial Number:	**BB1**	*Channel:*	**UV_VIS_1**
Sample Type:	**unknown**	*Wavelength:*	**220**
Control Program:	**YMC_120Min_100A_flow0_5**	*Bandwidth:*	**4**
Quantif. Method:	**default**	*Temperature/Column:*	**10**
Recording Time:	**25.2.2019 14:24**	*Flow ml/min:*	**0,500**
Run Time (min):	**15,36**	*Sample Amount:*	**1,0000**

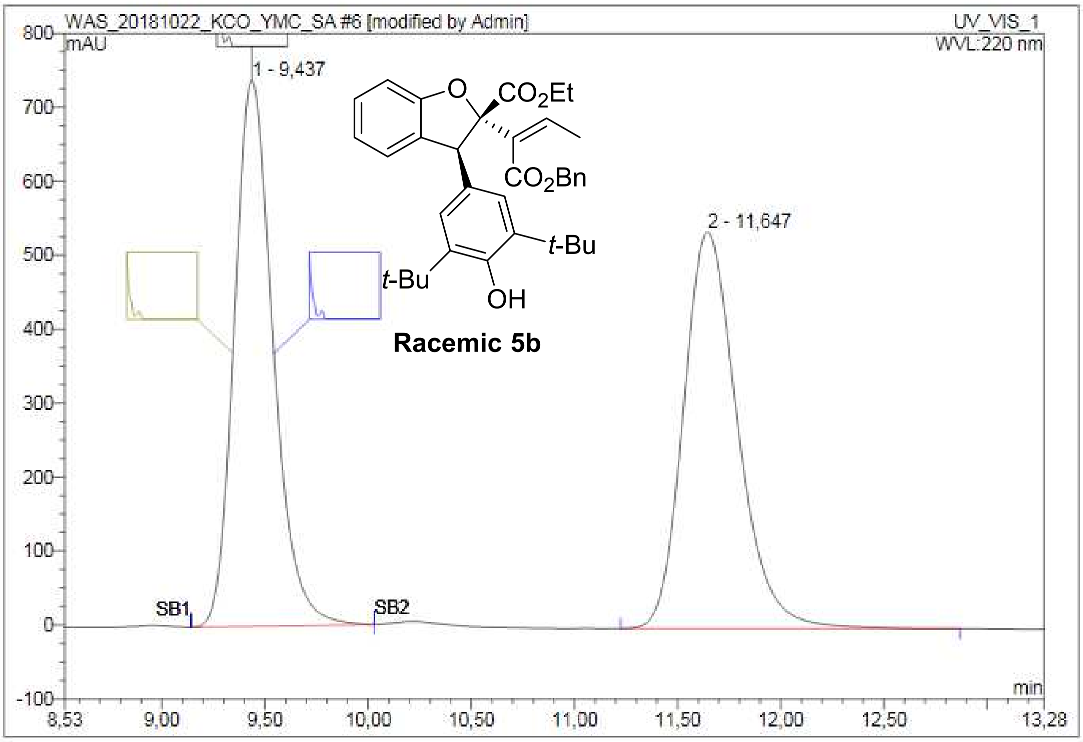

Racemic 5b

No.	Ret.Time min	Peak Name	Height mAU	Area mAU*min	Rel.Area %	Amount	Type
1	9,44	n.a.	738,502	161,401	49,75	n.a.	BMB
2	11,65	n.a.	535,817	163,030	50,25	n.a.	BMB
**Total:**			1274,318	324,431	100,00	0,000	

5 KCO-05-111-01hplc 90Hexan_10IPA0,5flow

Sample Name:	**KCO-05-111-01hplc 90Hexan_10IPA0,5flow**	*Injection Volume:*	**20,0**
Vial Number:	**BB1**	*Channel:*	**UV_VIS_1**
Sample Type:	**unknown**	*Wavelength:*	**220**
Control Program:	**YMC_120Min_100A_flow0_5**	*Bandwidth:*	**4**
Quantif. Method:	**default**	*Temperature/Column:*	**10**
Recording Time:	**25.2.2019 15:27**	*Flow ml/min:*	**0,500**
Run Time (min):	**15,16**	*Sample Amount:*	**1,0000**

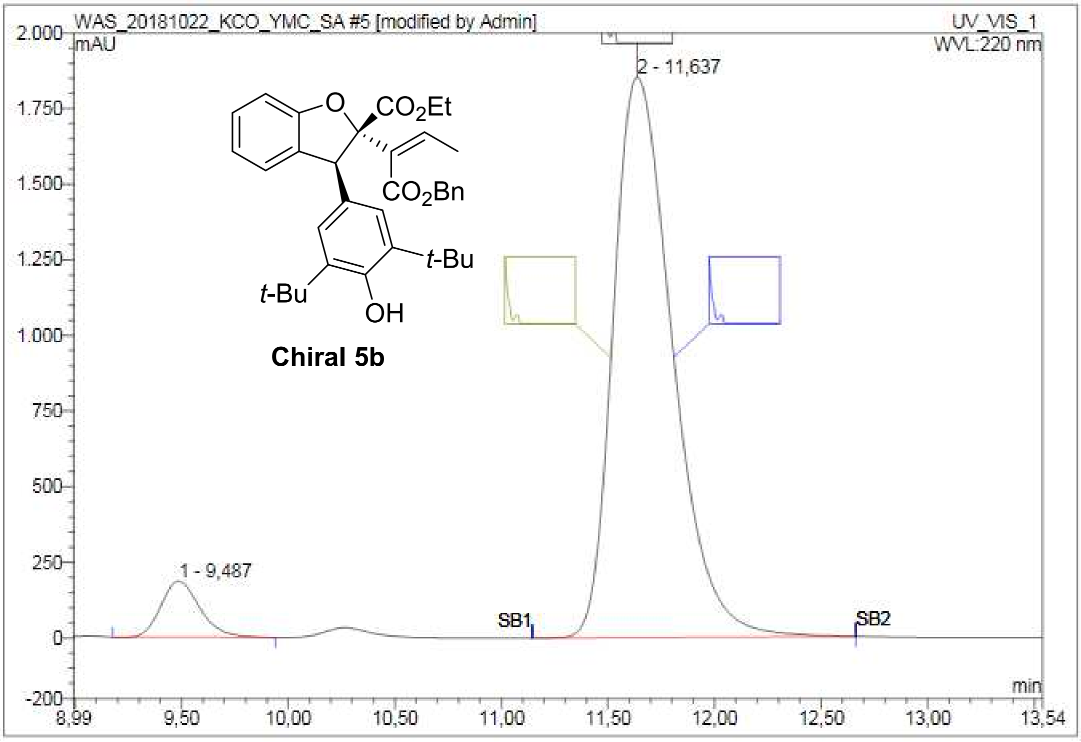

No.	Ret.Time min	Peak Name	Height mAU	Area mAU*min	Rel.Area %	Amount	Type
1	9,49	n.a.	185,687	39,350	6,19	n.a.	BMB*
2	11,64	n.a.	1852,120	596,124	93,81	n.a.	BMB*
Total:			2037,807	635,475	100,00	0,000	

12 KCO-05-104-02hplc 90Hexan_10IPA0,5flow

Sample Name:	**KCO-05-104-02hplc 90Hexan_10IPA0,5flow**	*Injection Volume:*	**20,0**
Vial Number:	**BB1**	*Channel:*	**UV_VIS_1**
Sample Type:	**unknown**	*Wavelength:*	**220**
Control Program:	**YMC_120Min_100A_flow0_5**	*Bandwidth:*	**4**
Quantif. Method:	**default**	*Temperature/Column:*	**10**
Recording Time:	**19.2.2019 18:55**	*Flow ml/min:*	**0,500**
Run Time (min):	**25,77**	*Sample Amount:*	**1,0000**

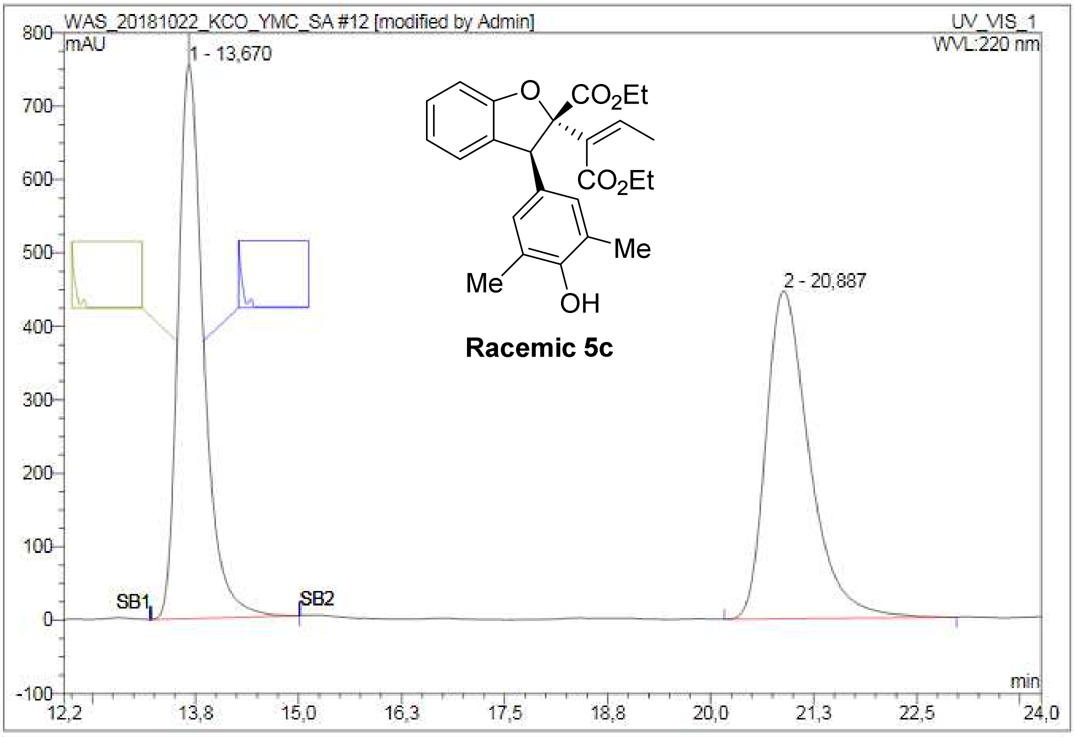

No.	Ret.Time min	Peak Name	Height mAU	Area mAU*min	Rel.Area %	Amount	Type
1	13,67	n.a.	755,071	272,183	49,94	n.a.	BMB
2	20,89	n.a.	446,622	272,800	50,06	n.a.	BMB
**Total:**			1201,693	544,983	100,00	0,000	

7 KCO-05-110-02 90Hexan_10IPA0,5flow

Sample Name:	**KCO-05-110-02 90Hexan_10IPA0,5flow**	*Injection Volume:*	**20,0**
Vial Number:	**BB1**	*Channel:*	**UV_VIS_1**
Sample Type:	**unknown**	*Wavelength:*	**220**
Control Program:	**YMC_120Min_100A_flow0_5**	*Bandwidth:*	**4**
Quantif. Method:	**default**	*Temperature/Column:*	**10**
Recording Time:	**24.2.2019 17:09**	*Flow ml/min:*	**0,500**
Run Time (min):	**25,39**	*Sample Amount:*	**1,0000**

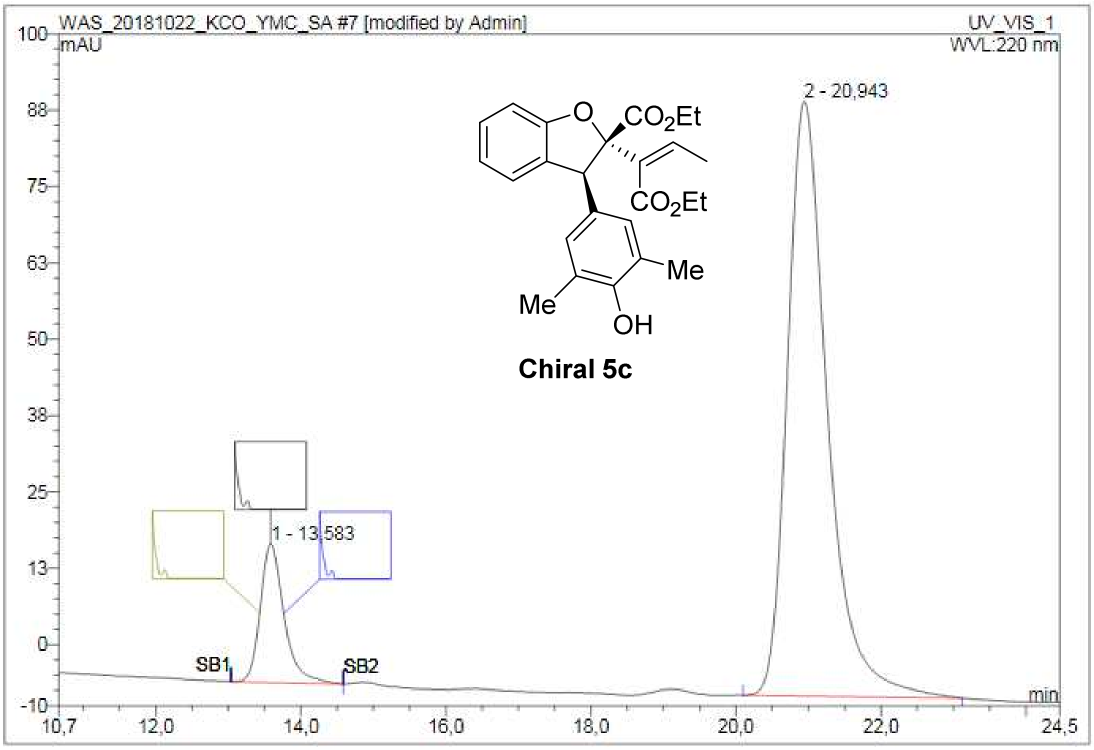

No.	Ret.Time min	Peak Name	Height mAU	Area mAU*min	Rel.Area %	Amount	Type
1	13,58	n.a.	22,713	8,642	12,17	n.a.	BMB
2	20,94	n.a.	97,276	62,343	87,83	n.a.	BMB
**Total:**			119,989	70,985	100,00	0,000	

45 KCO-05-051-01 98Hexan_2IPA_0,5flow

Sample Name:	**KCO-05-051-01 98Hexan_2IPA_0,5flow**	*Injection Volume:*	**20,0**
Vial Number:	**BA2**	*Channel:*	**UV_VIS_1**
Sample Type:	**unknown**	*Wavelength:*	**220**
Control Program:	**YMC120min_100A_flow0,5**	*Bandwidth:*	**4**
Quantif. Method:	**default**	*Temperature/Column:*	**10**
Recording Time:	**13.12.2018 17:47**	*Flow ml/min:*	**0,500**
Run Time (min):	**22,64**	*Sample Amount:*	**1,0000**

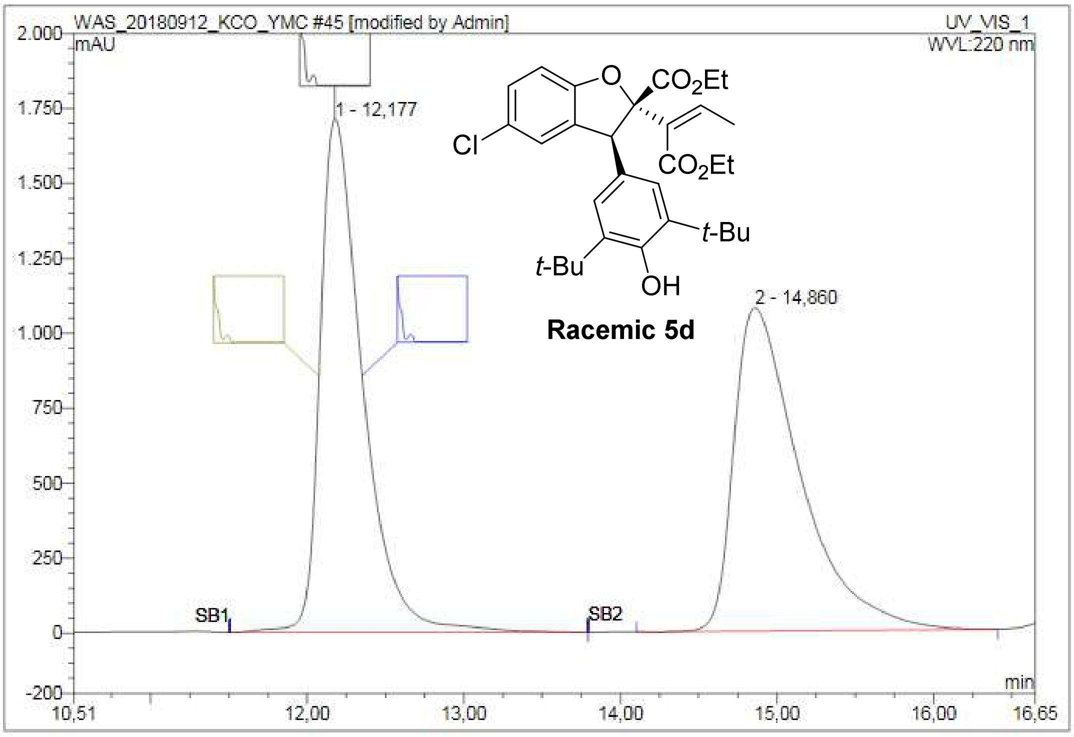

No.	Ret.Time min	Peak Name	Height mAU	Area mAU*min	Rel.Area %	Amount	Type
1	12,18	n.a.	1709,180	538,891	50,49	n.a.	BMB
2	14,86	n.a.	1076,644	528,404	49,51	n.a.	BMB*
**Total:**			2785,824	1067,296	100,00	0,000	

37 KCO-05-074-01 98Hexan_2IPA_0,5flow

Sample Name:	**KCO-05-074-01 98Hexan_2IPA_0,5flow**	*Injection Volume:*	**20,0**
Vial Number:	**BA1**	*Channel:*	**UV_VIS_1**
Sample Type:	**unknown**	*Wavelength:*	**220**
Control Program:	**YMC120min_100A_flow0,5**	*Bandwidth:*	**4**
Quantif. Method:	**default**	*Temperature/Column:*	**10**
Recording Time:	**10.1.2019 18:24**	*Flow ml/min:*	**0,500**
Run Time (min):	**17,13**	*Sample Amount:*	**1,0000**

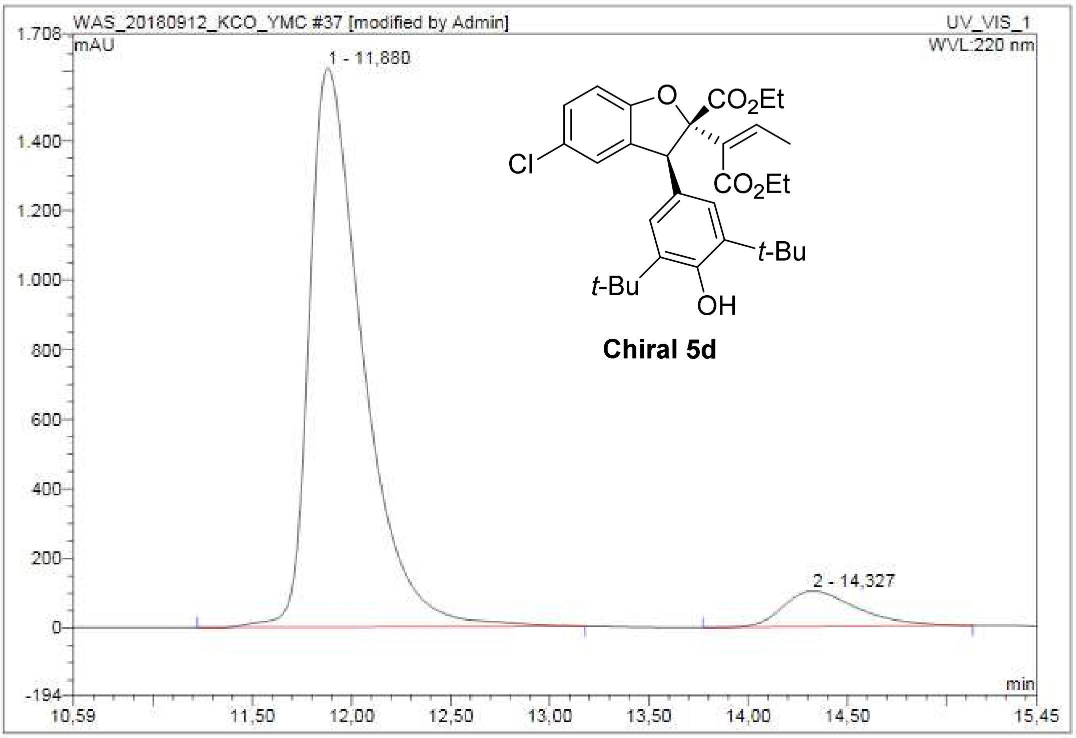

No.	Ret.Time min	Peak Name	Height mAU	Area mAU*min	Rel.Area %	Amount	Type
1	11,88	n.a.	1605,300	502,189	91,88	n.a.	BMB*
2	14,33	n.a.	102,466	44,377	8,12	n.a.	BMB*
**Total:**			1707,767	546,566	100,00	0,000	

49 KCO-05-046-01 95Hexan_5IPA_0,5flow

Sample Name:	**KCO-05-046-01 95Hexan_5IPA_0,5flow**	*Injection Volume:*	**20,0**
Vial Number:	**BA1**	*Channel:*	**UV_VIS_1**
Sample Type:	**unknown**	*Wavelength:*	**220**
Control Program:	**YMC120min_100A_flow0,5**	*Bandwidth:*	**4**
Quantif. Method:	**default**	*Temperature/Column:*	**10**
Recording Time:	**13.12.2018 10:10**	*Flow ml/min:*	**0,500**
Run Time (min):	**20,60**	*Sample Amount:*	**1,0000**

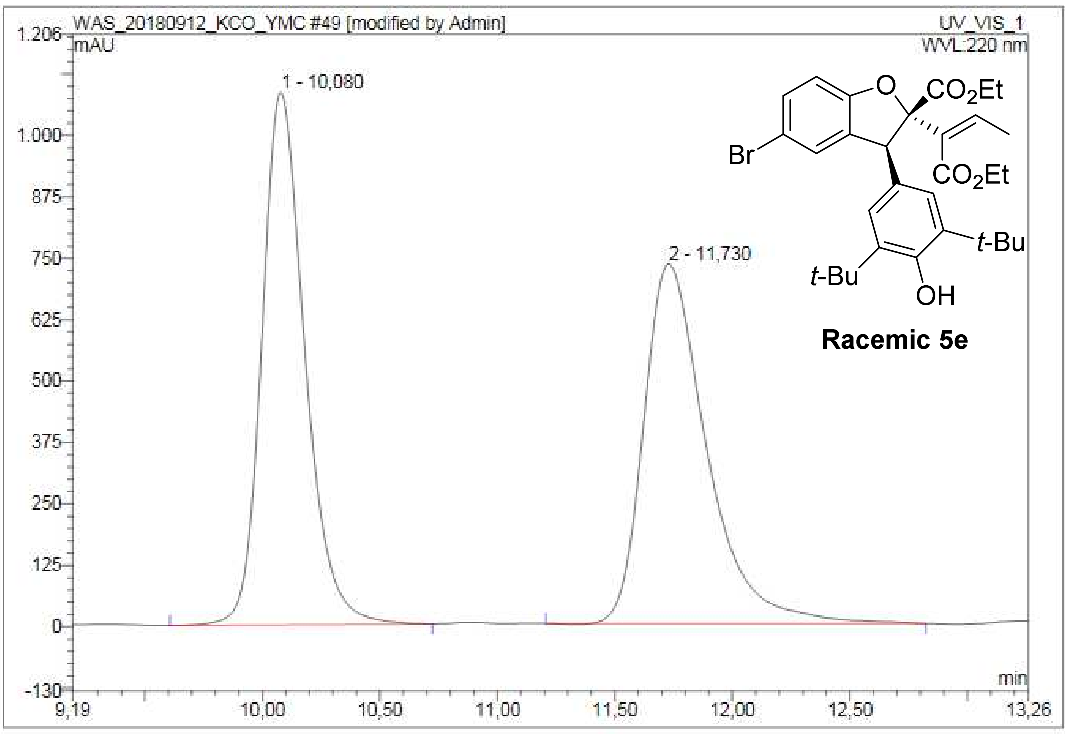

No.	Ret.Time min	Peak Name	Height mAU	Area mAU*min	Rel.Area %	Amount	Type
1	10,08	n.a.	1083,130	230,014	50,22	n.a.	BMB*
2	11,73	n.a.	731,580	228,012	49,78	n.a.	BMB*
**Total:**			1814,710	458,026	100,00	0,000	

42 KCO-05-064-01 95Hexan_5IPA_0,5flow

Sample Name:	**KCO-05-064-01 95Hexan_5IPA_0,5flow**	*Injection Volume:*	**20,0**
Vial Number:	**BA4**	*Channel:*	**UV_VIS_1**
Sample Type:	**unknown**	*Wavelength:*	**220**
Control Program:	**YMC120min_100A_flow0,5**	*Bandwidth:*	**4**
Quantif. Method:	**default**	*Temperature/Column:*	**10**
Recording Time:	**15.12.2018 12:50**	*Flow ml/min:*	**0,500**
Run Time (min):	**14,60**	*Sample Amount:*	**1,0000**

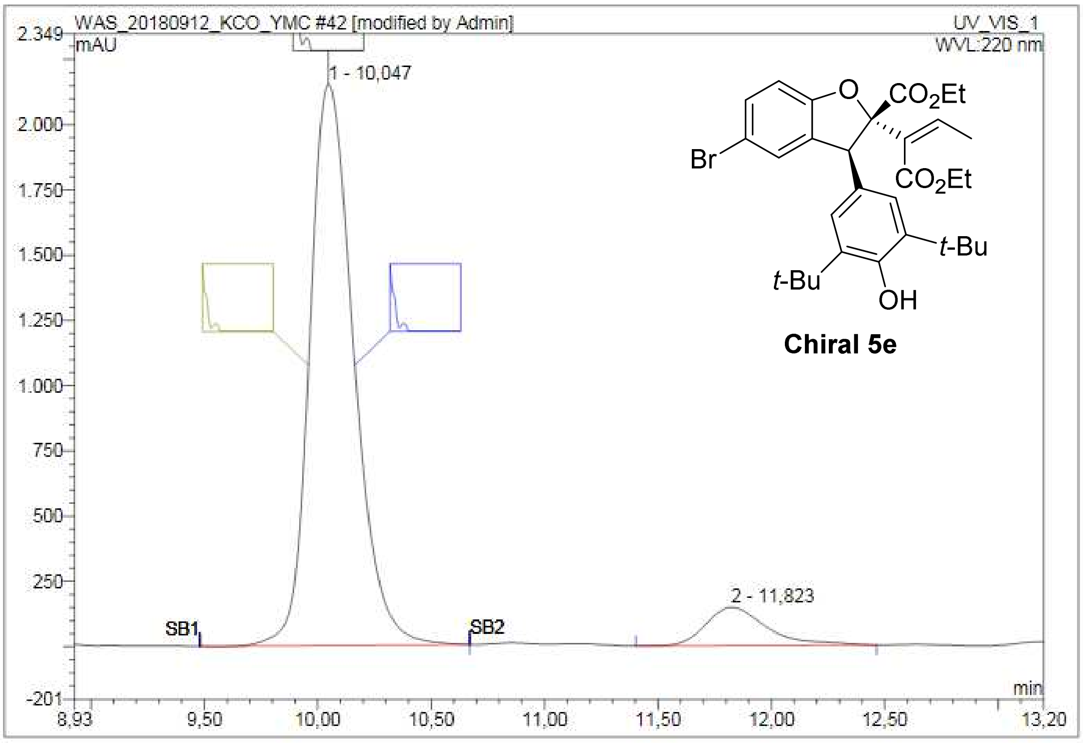

Chiral 5e

No.	Ret.Time min	Peak Name	Height mAU	Area mAU*min	Rel.Area %	Amount	Type
1	10,05	n.a.	2149,693	484,909	91,46	n.a.	BMB*
2	11,82	n.a.	146,656	45,266	8,54	n.a.	BMB*
Total:			2296,350	530,175	100,00	0,000	

32 KCO-05-071-01 100Hexan_1IPA_0,5flow

Sample Name:	**KCO-05-071-01 100Hexan_1IPA_0,5flow**	*Injection Volume:*	**20,0**
Vial Number:	**BA3**	*Channel:*	**UV_VIS_1**
Sample Type:	**unknown**	*Wavelength:*	**220**
Control Program:	**YMC120min_100A_flow0,5**	*Bandwidth:*	**4**
Quantif. Method:	**default**	*Temperature/Column:*	**10**
Recording Time:	**14.1.2019 12:37**	*Flow ml/min:*	**0,500**
Run Time (min):	**19,82**	*Sample Amount:*	**1,0000**

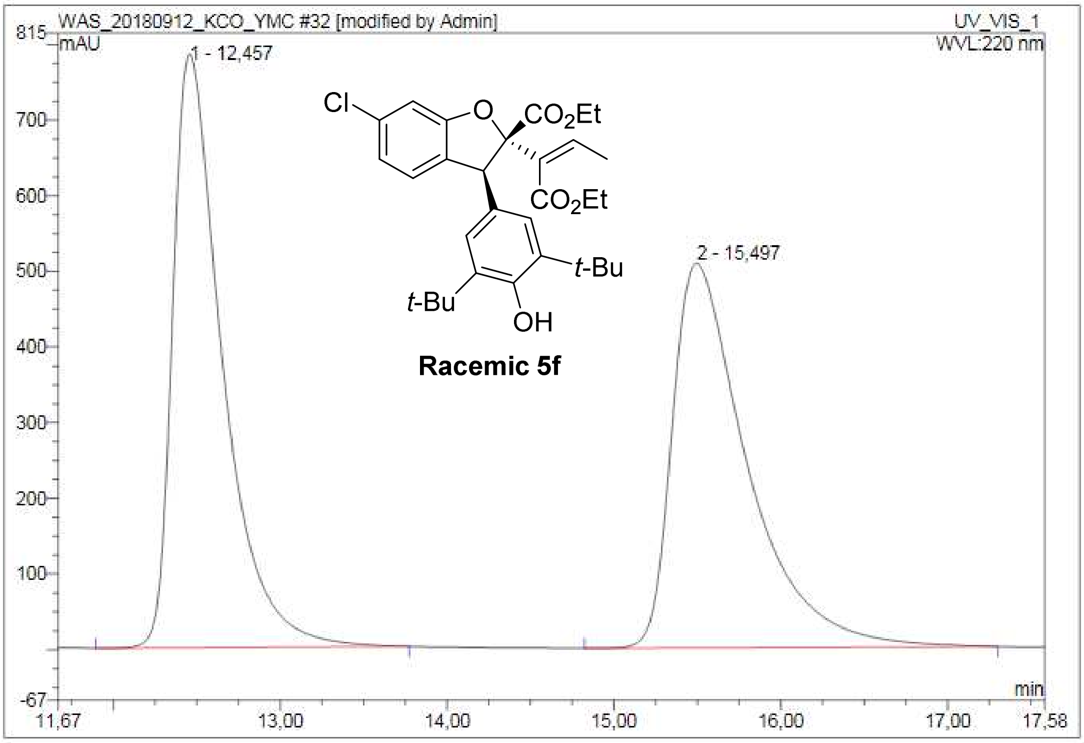

No.	Ret.Time min	Peak Name	Height mAU	Area mAU*min	Rel.Area %	Amount	Type
1	12,46	n.a.	785,077	271,386	50,61	n.a.	BMB*
2	15,50	n.a.	507,977	264,839	49,39	n.a.	BMB*
**Total:**			1293,055	536,225	100,00	0,000	

28 KCO-05-079-01 100Hexan_1IPA_0,5flow

Sample Name:	**KCO-05-079-01 100Hexan_1IPA_0,5flow**	*Injection Volume:*	**20,0**
Vial Number:	**BB1**	*Channel:*	**UV_VIS_1**
Sample Type:	**unknown**	*Wavelength:*	**220**
Control Program:	**YMC120min_100A_flow0,5**	*Bandwidth:*	**4**
Quantif. Method:	**default**	*Temperature/Column:*	**10**
Recording Time:	**18.1.2019 16:03**	*Flow ml/min:*	**0,500**
Run Time (min):	**18,22**	*Sample Amount:*	**1,0000**

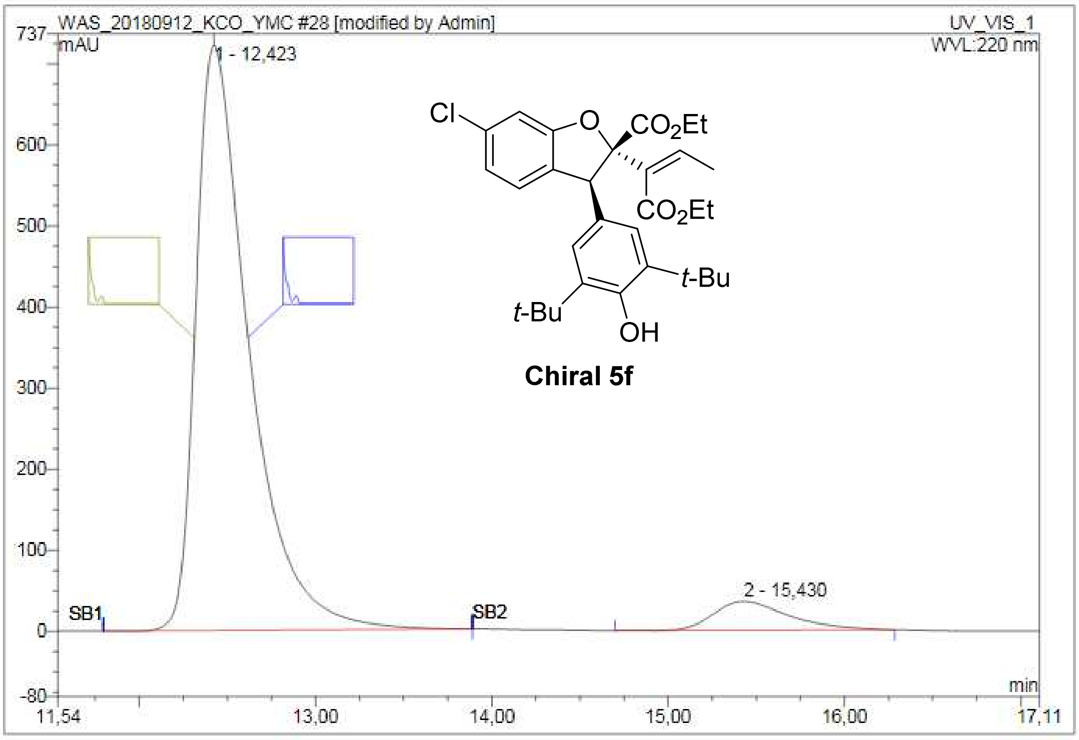

No.	Ret.Time min	Peak Name	Height mAU	Area mAU*min	Rel.Area %	Amount	Type
1	12,42	n.a.	721,541	252,972	93,56	n.a.	BMB*
2	15,43	n.a.	35,531	17,416	6,44	n.a.	BMB*
Total:			757,072	270,389	100,00	0,000	

3 KCO-05-102-01hplc 100Hexan_1IPA_0,5flow

Sample Name:	**KCO-05-102-01hplc 100Hexan_1IPA_0,5flow**	*Injection Volume:*	**20,0**
Vial Number:	**BB1**	*Channel:*	**UV_VIS_1**
Sample Type:	**unknown**	*Wavelength:*	**220**
Control Program:	**YMC120min_100A_flow0,5**	*Bandwidth:*	**4**
Quantif. Method:	**default**	*Temperature/Column:*	**10**
Recording Time:	**14.2.2019 16:19**	*Flow ml/min:*	**0,500**
Run Time (min):	**31,85**	*Sample Amount:*	**1,0000**

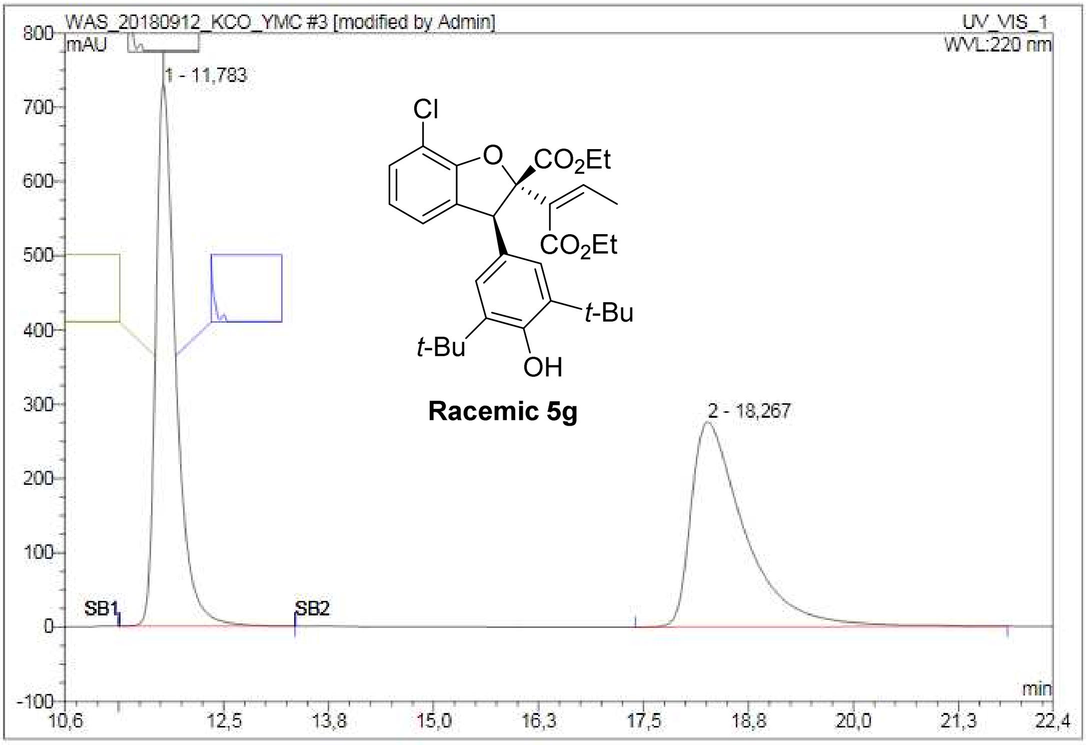

No.	Ret.Time min	Peak Name	Height mAU	Area mAU*min	Rel.Area %	Amount	Type
1	11,78	n.a.	728,339	202,981	49,76	n.a.	BMB
2	18,27	n.a.	275,568	204,911	50,24	n.a.	BMB
Total:			1003,908	407,892	100,00	0,000	

2 KCO-05-103-01hplc 100Hexan_1IPA_0,5flow

Sample Name:	**KCO-05-103-01hplc 100Hexan_1IPA_0,5flow**	*Injection Volume:*	**20,0**
Vial Number:	**BB2**	*Channel:*	**UV_VIS_1**
Sample Type:	**unknown**	*Wavelength:*	**220**
Control Program:	**YMC120min_100A_flow0,5**	*Bandwidth:*	**4**
Quantif. Method:	**default**	*Temperature/Column:*	**10**
Recording Time:	**14.2.2019 17:30**	*Flow ml/min:*	**0,500**
Run Time (min):	**23,68**	*Sample Amount:*	**1,0000**

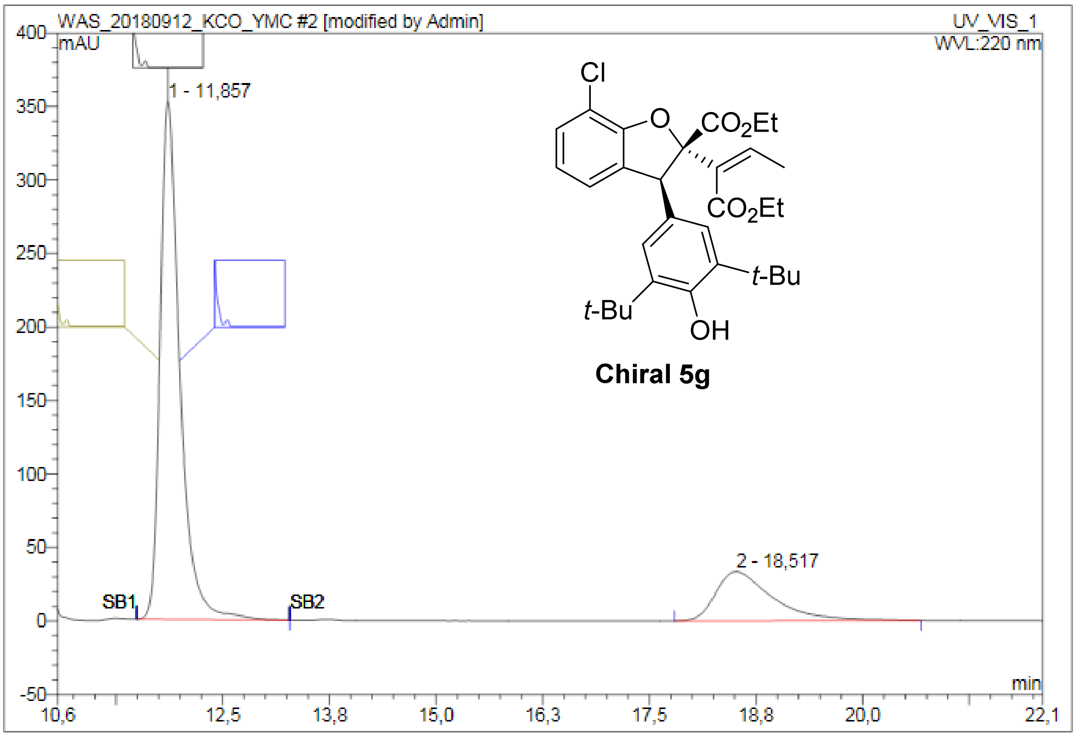

No.	Ret.Time min	Peak Name	Height mAU	Area mAU*min	Rel.Area %	Amount	Type
1	11,86	n.a.	352,387	94,440	78,69	n.a.	BMB
2	18,52	n.a.	33,439	25,580	21,31	n.a.	BMB
**Total:**			385,826	120,021	100,00	0,000	

62 KCO-04-186-01 95Hexan_5IPA_0,5flow

Sample Name:	**KCO-04-186-01 95Hexan_5IPA_0,5flow**	*Injection Volume:*	**20,0**
Vial Number:	**RE1**	*Channel:*	**UV_VIS_1**
Sample Type:	**unknown**	*Wavelength:*	**220**
Control Program:	**YMC60min_100A_flow0,5**	*Bandwidth:*	**4**
Quantif. Method:	**default**	*Temperature/Column:*	**10**
Recording Time:	**6.11.2018 14:45**	*Flow ml/min:*	**0,500**
Run Time (min):	**24,36**	*Sample Amount:*	**1,0000**

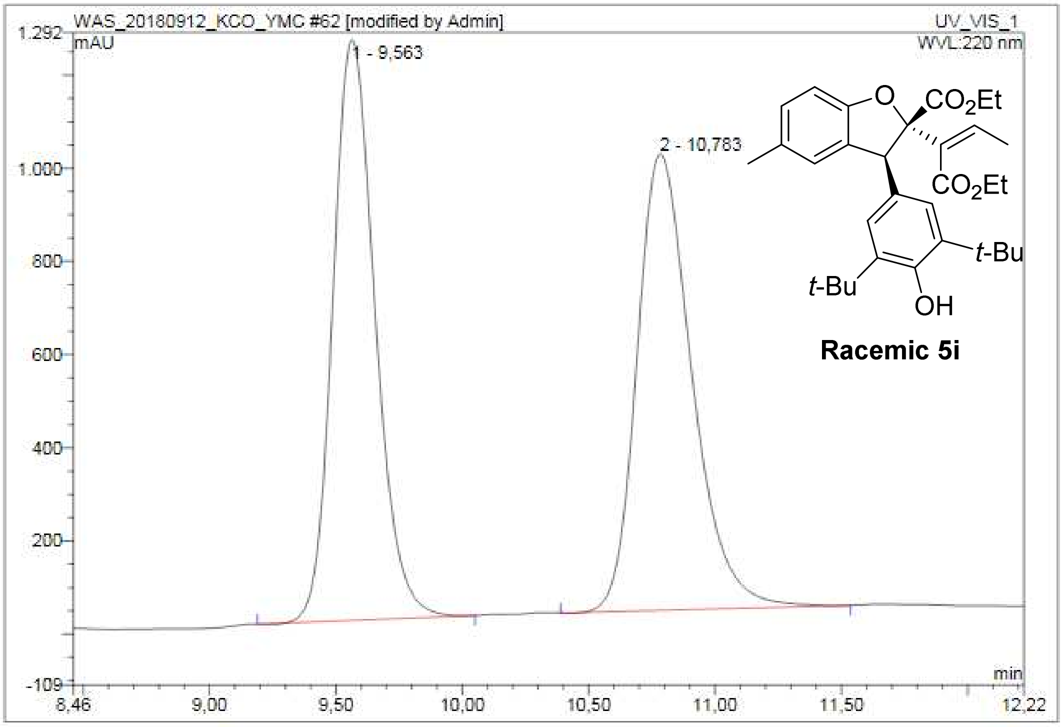

No.	Ret.Time min	Peak Name	Height mAU	Area mAU*min	Rel.Area %	Amount	Type
1	9,56	n.a.	1246,240	241,750	49,43	n.a.	BMB*
2	10,78	n.a.	979,796	247,374	50,57	n.a.	BMB*
**Total:**			2226,036	489,124	100,00	0,000	

50 KCO-05-047-03prep 95Hexan_5IPA_0,5flow

Sample Name:	**KCO-05-047-03prep 95Hexan_5IPA_0,5flow**	*Injection Volume:*	**20,0**
Vial Number:	**BA2**	*Channel:*	**UV_VIS_1**
Sample Type:	**unknown**	*Wavelength:*	**220**
Control Program:	**YMC60min_100A_flow0,5**	*Bandwidth:*	**4**
Quantif. Method:	**default**	*Temperature/Column:*	**10**
Recording Time:	**6.12.2018 20:19**	*Flow ml/min:*	**0,500**
Run Time (min):	**15,84**	*Sample Amount:*	**1,0000**

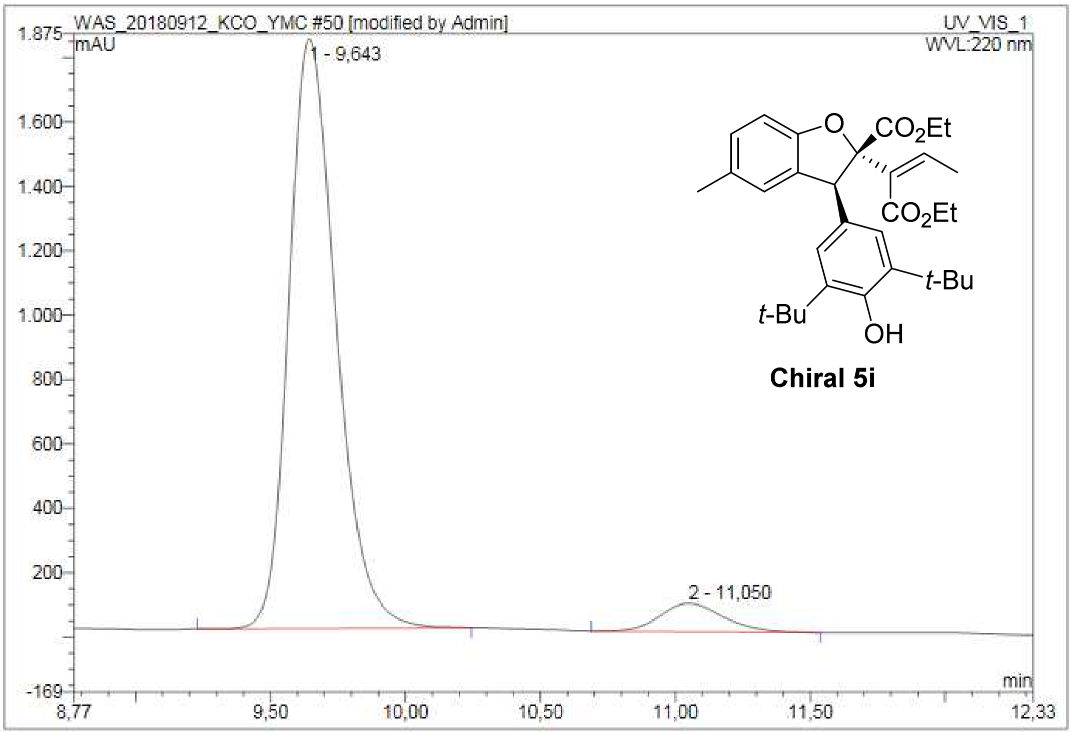

No.	Ret.Time min	Peak Name	Height mAU	Area mAU*min	Rel.Area %	Amount	Type
1	9,64	n.a.	1829,683	371,506	93,93	n.a.	BMB*
2	11,05	n.a.	88,398	24,021	6,07	n.a.	BMB*
**Total:**			1918,081	395,527	100,00	0,000	

29 KCO-05-080-01 98Hexan_2IPA_0,5flow

Sample Name:	**KCO-05-080-01 98Hexan_2IPA_0,5flow**	*Injection Volume:*	**20,0**
Vial Number:	**BA2**	*Channel:*	**UV_VIS_1**
Sample Type:	**unknown**	*Wavelength:*	**220**
Control Program:	**YMC120min_100A_flow0,5**	*Bandwidth:*	**4**
Quantif. Method:	**default**	*Temperature/Column:*	**10**
Recording Time:	**16.1.2019 16:59**	*Flow ml/min:*	**0,500**
Run Time (min):	**14,37**	*Sample Amount:*	**1,0000**

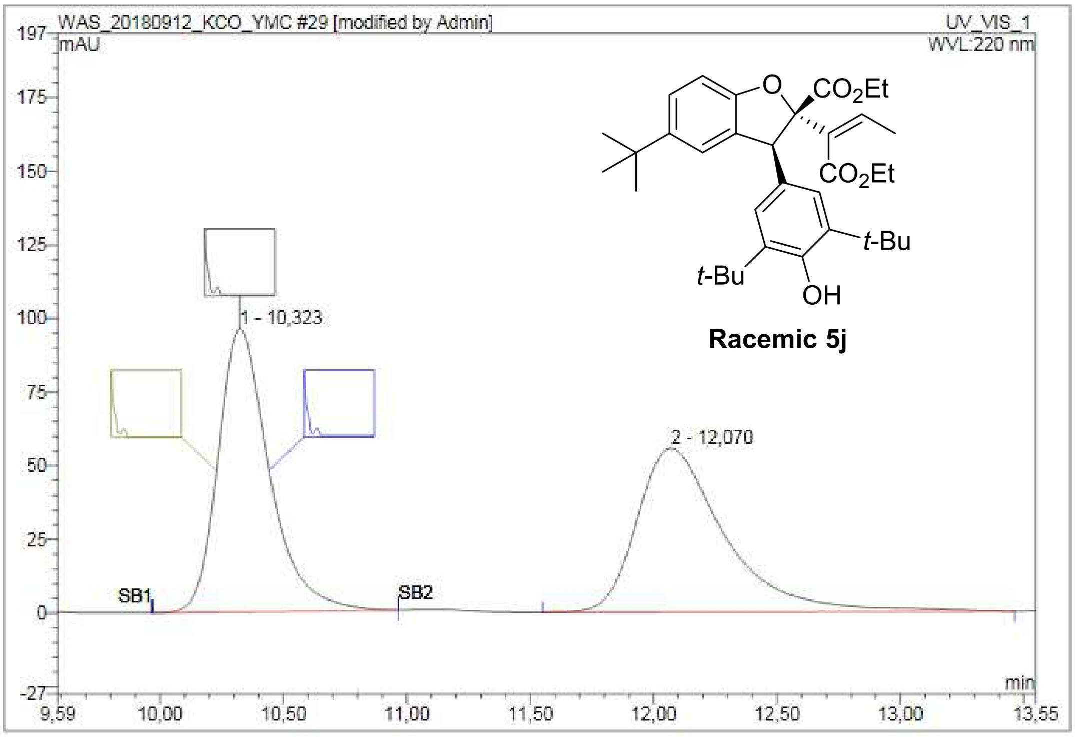

No.	Ret.Time min	Peak Name	Height mAU	Area mAU*min	Rel.Area %	Amount	Type
1	10,32	n.a.	96,017	23,146	50,19	n.a.	BMB
2	12,07	n.a.	55,506	22,969	49,81	n.a.	BMB
**Total:**			151,523	46,115	100,00	0,000	

27 KCO-05-082-01 98Hexan_2IPA_0,5flow

Sample Name:	**KCO-05-082-01 98Hexan_2IPA_0,5flow**	*Injection Volume:*	**20,0**
Vial Number:	**BA1**	*Channel:*	**UV_VIS_1**
Sample Type:	**unknown**	*Wavelength:*	**220**
Control Program:	**YMC120min_100A_flow0,5**	*Bandwidth:*	**4**
Quantif. Method:	**default**	*Temperature/Column:*	**10**
Recording Time:	**22.1.2019 17:59**	*Flow ml/min:*	**0,500**
Run Time (min):	**15,00**	*Sample Amount:*	**1,0000**

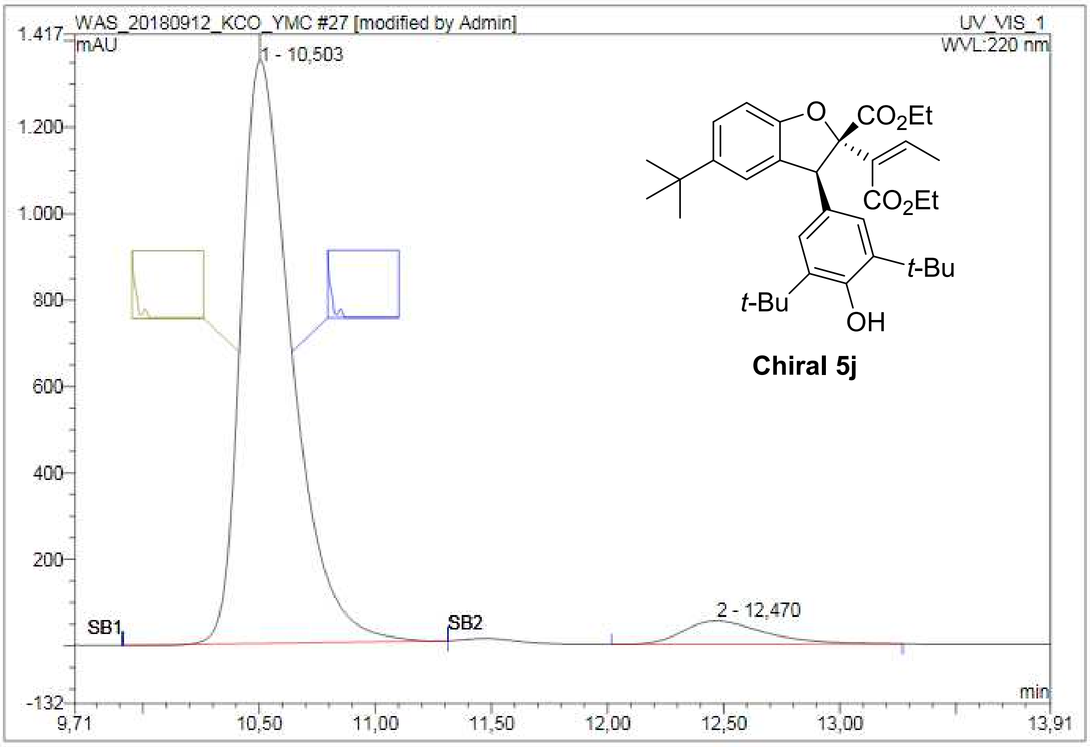

Chiral 5j

No.	Ret.Time min	Peak Name	Height mAU	Area mAU*min	Rel.Area %	Amount	Type
1	10,50	n.a.	1348,444	343,036	93,95	n.a.	BMB*
2	12,47	n.a.	54,296	22,083	6,05	n.a.	BMB*
**Total:**			1402,739	365,120	100,00	0,000	

38 KCO-05-059-01 99Hexan_1IPA_0,5flow

Sample Name:	**KCO-05-059-01 99Hexan_1IPA_0,5flow**	*Injection Volume:*	**20,0**
Vial Number:	**BA1**	*Channel:*	**UV_VIS_1**
Sample Type:	**unknown**	*Wavelength:*	**220**
Control Program:	**YMC120min_100A_flow0,5**	*Bandwidth:*	**4**
Quantif. Method:	**default**	*Temperature/Column:*	**10**
Recording Time:	**4.1.2019 11:49**	*Flow ml/min:*	**0,500**
Run Time (min):	**23,74**	*Sample Amount:*	**1,0000**

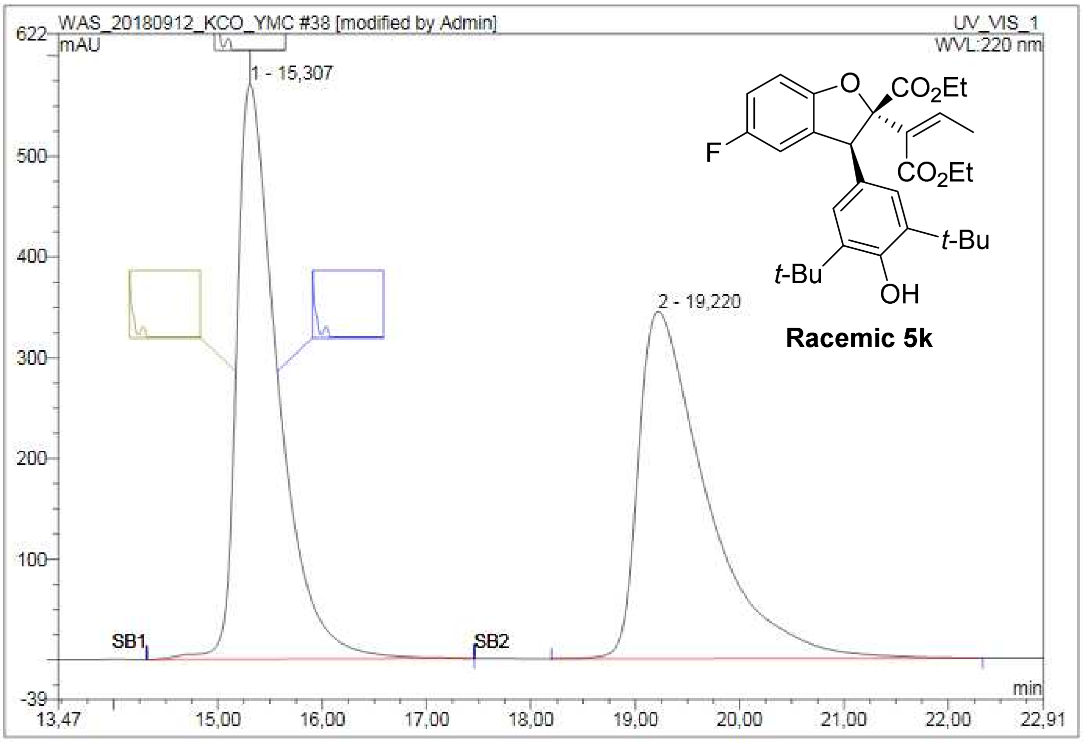

No.	Ret.Time min	Peak Name	Height mAU	Area mAU*min	Rel.Area %	Amount	Type
1	15,31	n.a.	571,043	262,279	50,07	n.a.	BMB
2	19,22	n.a.	344,511	261,538	49,93	n.a.	BMB
**Total:**			915,554	523,818	100,00	0,000	

31 KCO-05-078-01 99Hexan_1IPA_0,5flow

Sample Name:	**KCO-05-078-01 99Hexan_1IPA_0,5flow**	*Injection Volume:*	**20,0**
Vial Number:	**BA1**	*Channel:*	**UV_VIS_1**
Sample Type:	**unknown**	*Wavelength:*	**220**
Control Program:	**YMC120min_100A_flow0,5**	*Bandwidth:*	**4**
Quantif. Method:	**default**	*Temperature/Column:*	**10**
Recording Time:	**15.1.2019 17:31**	*Flow ml/min:*	**0,500**
Run Time (min):	**23,34**	*Sample Amount:*	**1,0000**

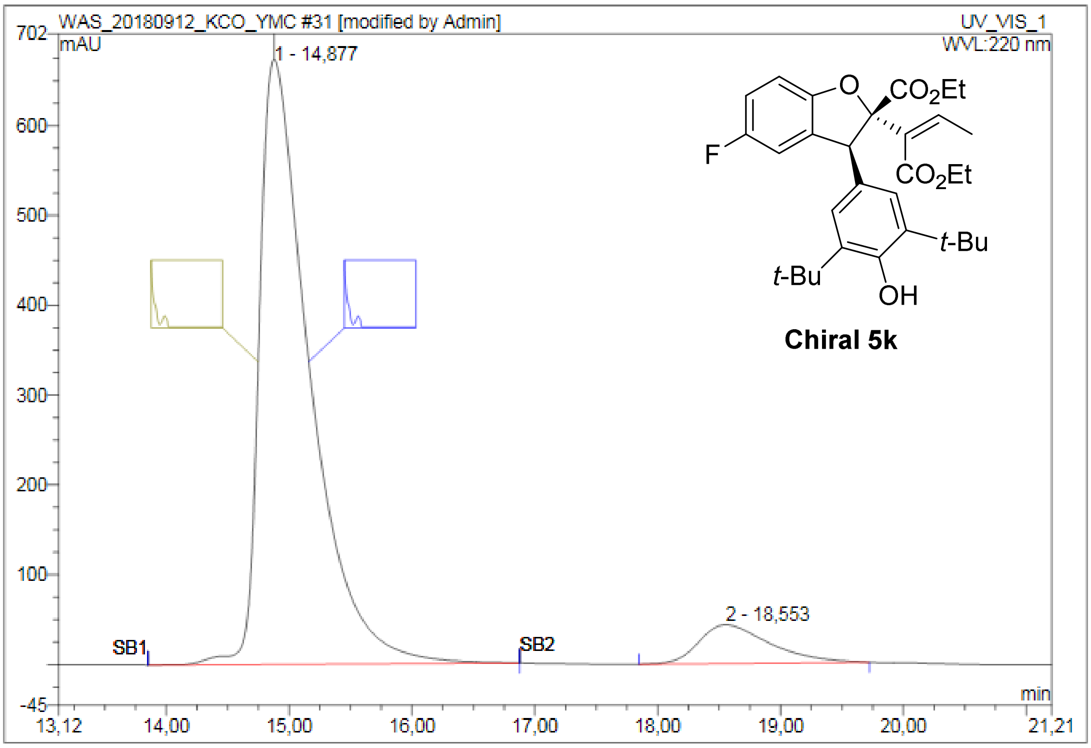

No.	Ret.Time min	Peak Name	Height mAU	Area mAU*min	Rel.Area %	Amount	Type
1	14,88	n.a.	673,454	320,398	91,76	n.a.	BMB*
2	18,55	n.a.	43,014	28,759	8,24	n.a.	BMB*
**Total:**			716,468	349,157	100,00	0,000	

17 KCO-05-089-02hplc 98Hexan_2IPA0,5flow

Sample Name:	**KCO-05-089-02hplc 98Hexan_2IPA0,5flow**	*Injection Volume:*	**20,0**
Vial Number:	**BB1**	*Channel:*	**UV_VIS_1**
Sample Type:	**unknown**	*Wavelength:*	**220**
Control Program:	**YMC_120Min_100A_flow0_5**	*Bandwidth:*	**4**
Quantif. Method:	**default**	*Temperature/Column:*	**10**
Recording Time:	**12.2.2019 16:35**	*Flow ml/min:*	**0,500**
Run Time (min):	**15,47**	*Sample Amount:*	**1,0000**

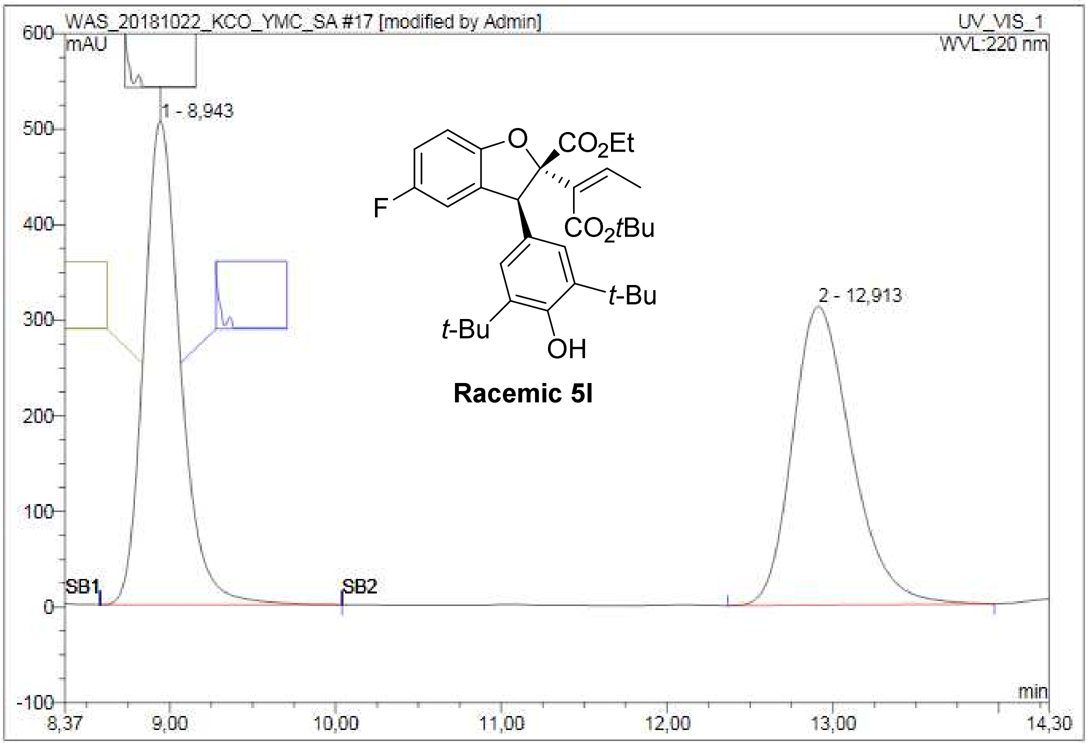

Racemic 5l

No.	Ret.Time min	Peak Name	Height mAU	Area mAU*min	Rel.Area %	Amount	Type
1	8,94	n.a.	506,290	129,763	50,11	n.a.	BMB*
2	12,91	n.a.	312,321	129,197	49,89	n.a.	BMB
**Total:**			818,611	258,960	100,00	0,000	

9 KCO-05-109-02hplc 98Hexan_2IPA0,5flow

Sample Name:	**KCO-05-109-02hplc 98Hexan_2IPA0,5flow**	*Injection Volume:*	**20,0**
Vial Number:	**BB1**	*Channel:*	**UV_VIS_1**
Sample Type:	**unknown**	*Wavelength:*	**220**
Control Program:	**YMC_120Min_100A_flow0_5**	*Bandwidth:*	**4**
Quantif. Method:	**default**	*Temperature/Column:*	**10**
Recording Time:	**21.2.2019 19:46**	*Flow ml/min:*	**0,500**
Run Time (min):	**16,42**	*Sample Amount:*	**1,0000**

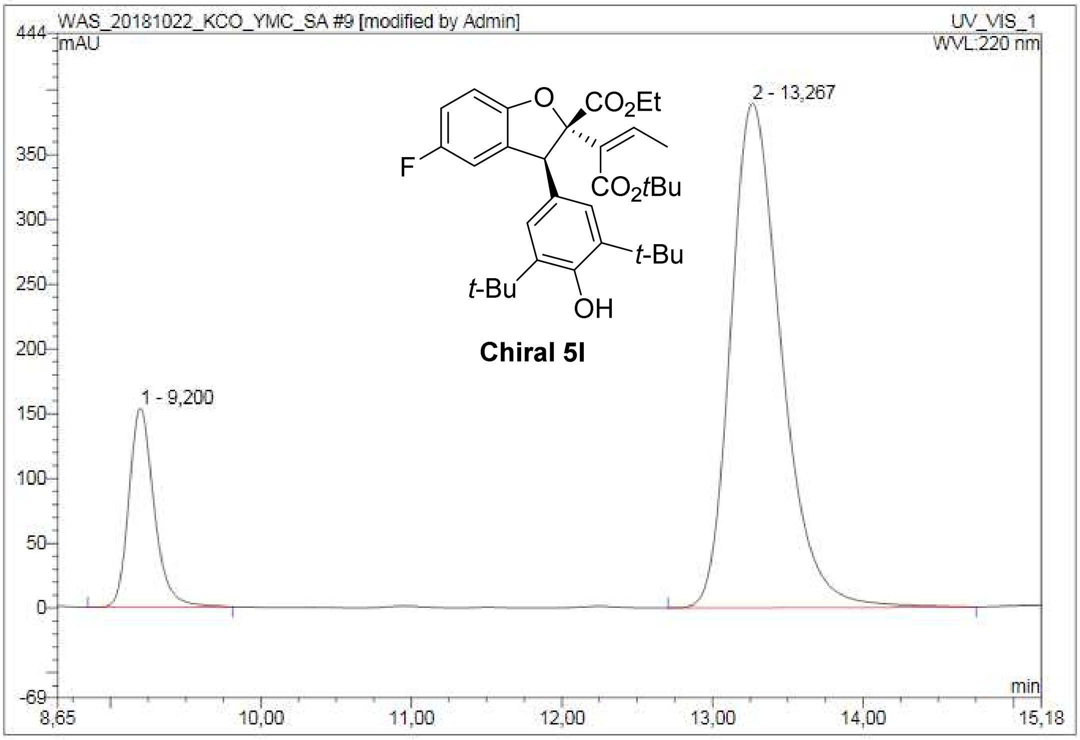

No.	Ret.Time min	Peak Name	Height mAU	Area mAU*min	Rel.Area %	Amount	Type
1	9,20	n.a.	153,423	29,732	16,42	n.a.	BMB*
2	13,27	n.a.	389,413	151,329	83,58	n.a.	BMB
**Total:**			542,836	181,062	100,00	0,000	

43 KCO-05-056-01 95Hexan_5IPA_0,5flow

Sample Name:	**KCO-05-056-01 95Hexan_5IPA_0,5flow**	*Injection Volume:*	**20,0**
Vial Number:	**BA3**	*Channel:*	**UV_VIS_1**
Sample Type:	**unknown**	*Wavelength:*	**220**
Control Program:	**YMC120min_100A_flow0,5**	*Bandwidth:*	**4**
Quantif. Method:	**default**	*Temperature/Column:*	**10**
Recording Time:	**15.12.2018 11:09**	*Flow ml/min:*	**0,500**
Run Time (min):	**20,51**	*Sample Amount:*	**1,0000**

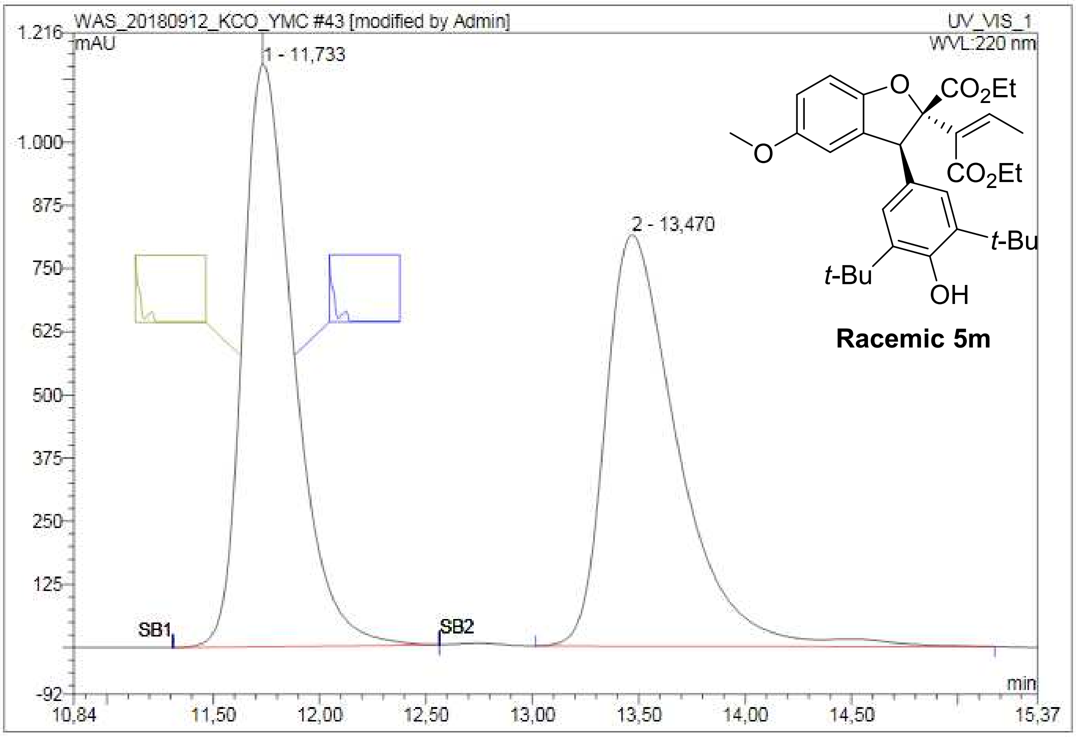

No.	Ret.Time min	Peak Name	Height mAU	Area mAU*min	Rel.Area %	Amount	Type
1	11,73	n.a.	1152,120	320,942	49,85	n.a.	BMB
2	13,47	n.a.	813,294	322,915	50,15	n.a.	BMB*
**Total:**			1965,413	643,857	100,00	0,000	

35 KCO-05-076-01 95Hexan_5IPA_0,5flow

Sample Name:	**KCO-05-076-01 95Hexan_5IPA_0,5flow**	*Injection Volume:*	**20,0**
Vial Number:	**BA2**	*Channel:*	**UV_VIS_1**
Sample Type:	**unknown**	*Wavelength:*	**220**
Control Program:	**YMC120min_100A_flow0,5**	*Bandwidth:*	**4**
Quantif. Method:	**default**	*Temperature/Column:*	**10**
Recording Time:	**11.1.2019 17:27**	*Flow ml/min:*	**0,500**
Run Time (min):	**16,14**	*Sample Amount:*	**1,0000**

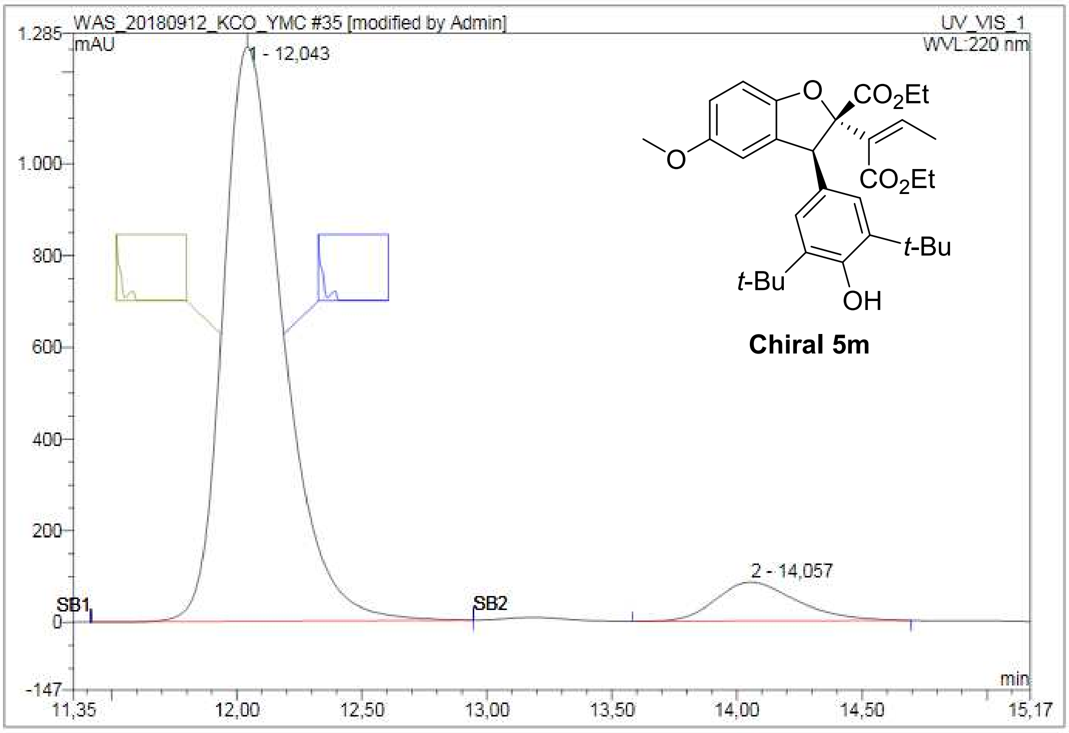

No.	Ret.Time min	Peak Name	Height mAU	Area mAU*min	Rel.Area %	Amount	Type
1	12,04	n.a.	1253,653	349,069	91,60	n.a.	BMB*
2	14,06	n.a.	84,806	32,006	8,40	n.a.	BMB*
**Total:**			1338,459	381,075	100,00	0,000	

21 KCO-05-087-01hplc 95Hexan_5IPA0,5flow

Sample Name:	**KCO-05-087-01hplc 95Hexan_5IPA0,5flow**	*Injection Volume:*	**20,0**
Vial Number:	**GA1**	*Channel:*	**UV_VIS_1**
Sample Type:	**unknown**	*Wavelength:*	**220**
Control Program:	**YMC_120Min_100A_flow0_5**	*Bandwidth:*	**4**
Quantif. Method:	**default**	*Temperature/Column:*	**10**
Recording Time:	**10.2.2019 13:32**	*Flow ml/min:*	**0,500**
Run Time (min):	**16,16**	*Sample Amount:*	**1,0000**

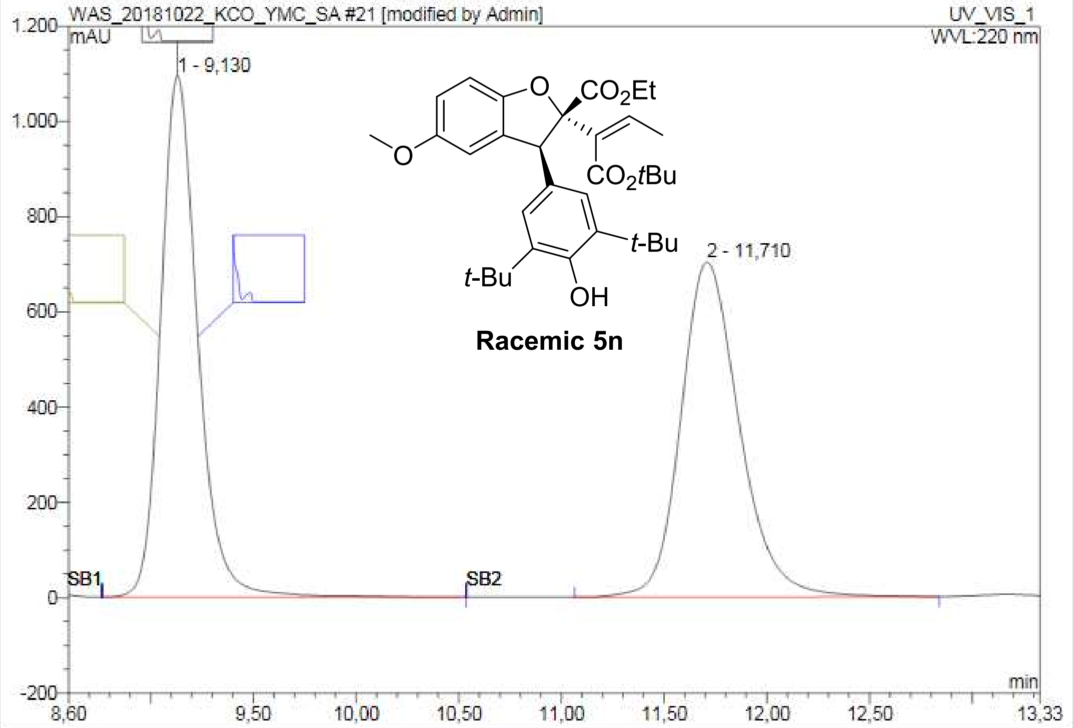

Racemic 5n

No.	Ret.Time min	Peak Name	Height mAU	Area mAU*min	Rel.Area %	Amount	Type
1	9,13	n.a.	1094,895	228,873	49,61	n.a.	BMB
2	11,71	n.a.	703,018	232,441	50,39	n.a.	BMB
Total:			1797,913	461,314	100,00	0,000	

14 KCO-05-105-01hplc 95Hexan_5IPA0,5flow

Sample Name:	**KCO-05-105-01hplc 95Hexan_5IPA0,5flow**	*Injection Volume:*	**20,0**
Vial Number:	**BB1**	*Channel:*	**UV_VIS_1**
Sample Type:	**unknown**	*Wavelength:*	**220**
Control Program:	**YMC_120Min_100A_flow0_5**	*Bandwidth:*	**4**
Quantif. Method:	**default**	*Temperature/Column:*	**10**
Recording Time:	**18.2.2019 18:49**	*Flow ml/min:*	**0,500**
Run Time (min):	**14,58**	*Sample Amount:*	**1,0000**

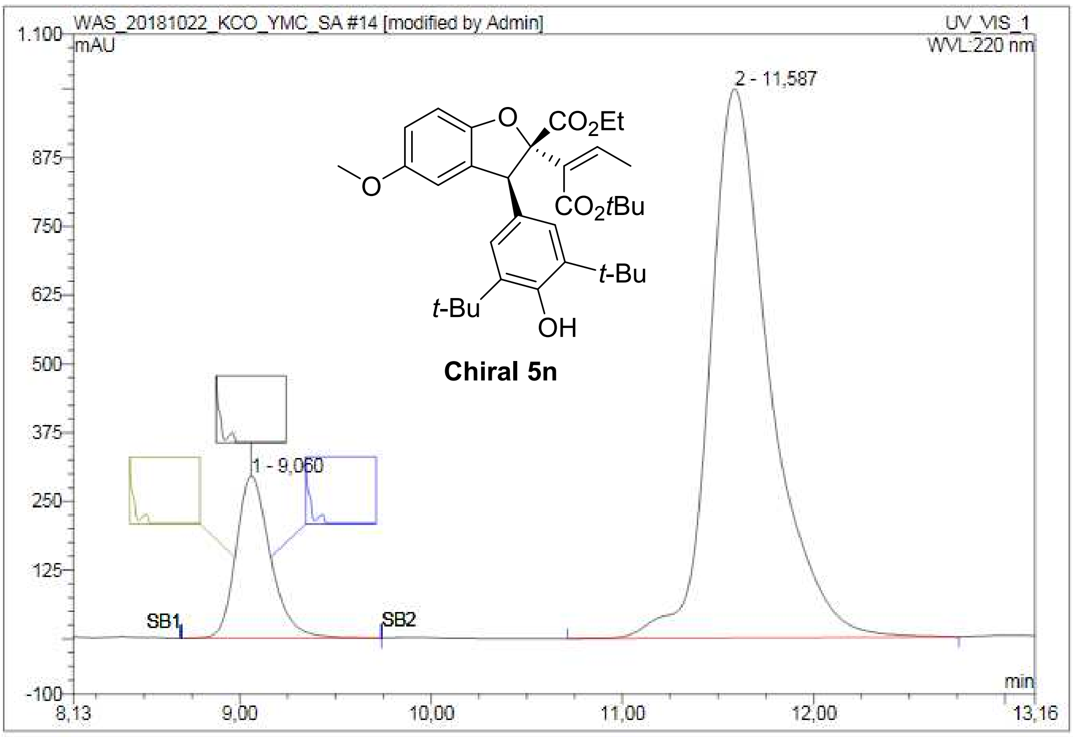

No.	Ret.Time min	Peak Name	Height mAU	Area mAU*min	Rel.Area %	Amount	Type
1	9,06	n.a.	295,006	61,589	14,54	n.a.	BMB
2	11,59	n.a.	998,463	361,968	85,46	n.a.	BMB
**Total:**			1293,469	423,558	100,00	0,000	

2 KCO-05-106-02hplc 90Hexan_10IPA0,5flow

Sample Name:	**KCO-05-106-02hplc 90Hexan_10IPA0,5flow**	*Injection Volume:*	**20,0**
Vial Number:	**BB1**	*Channel:*	**UV_VIS_1**
Sample Type:	**unknown**	*Wavelength:*	**220**
Control Program:	**YMC_120Min_100A_flow0_5**	*Bandwidth:*	**4**
Quantif. Method:	**default**	*Temperature/Column:*	**10**
Recording Time:	**25.2.2019 19:44**	*Flow ml/min:*	**0,500**
Run Time (min):	**19,76**	*Sample Amount:*	**1,0000**

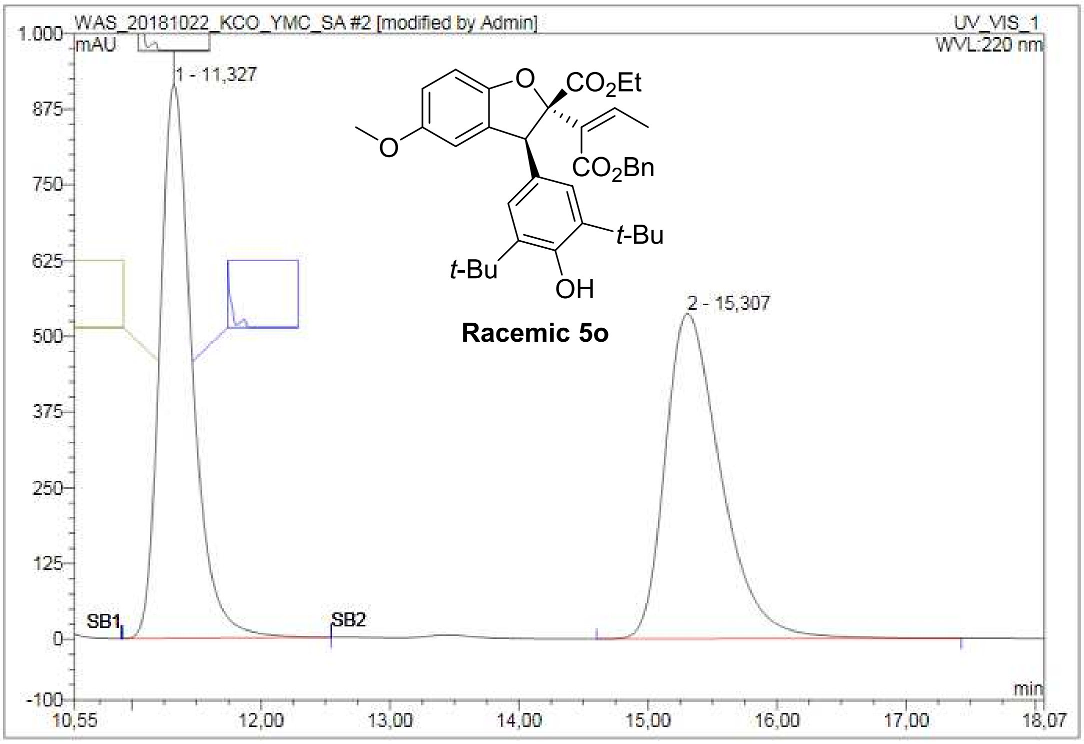

No.	Ret.Time min	Peak Name	Height mAU	Area mAU*min	Rel.Area %	Amount	Type
1	11,33	n.a.	914,171	265,475	49,68	n.a.	BMB
2	15,31	n.a.	537,293	268,899	50,32	n.a.	BMB
**Total:**			1451,464	534,374	100,00	0,000	

3 KCO-05-112-01hplc 90Hexan_10IPA0,5flow

Sample Name:	**KCO-05-112-01hplc 90Hexan_10IPA0,5flow**	*Injection Volume:*	**20,0**
Vial Number:	**BB1**	*Channel:*	**UV_VIS_1**
Sample Type:	**unknown**	*Wavelength:*	**220**
Control Program:	**YMC_120Min_100A_flow0_5**	*Bandwidth:*	**4**
Quantif. Method:	**default**	*Temperature/Column:*	**10**
Recording Time:	**25.2.2019 18:55**	*Flow ml/min:*	**0,500**
Run Time (min):	**21,73**	*Sample Amount:*	**1,0000**

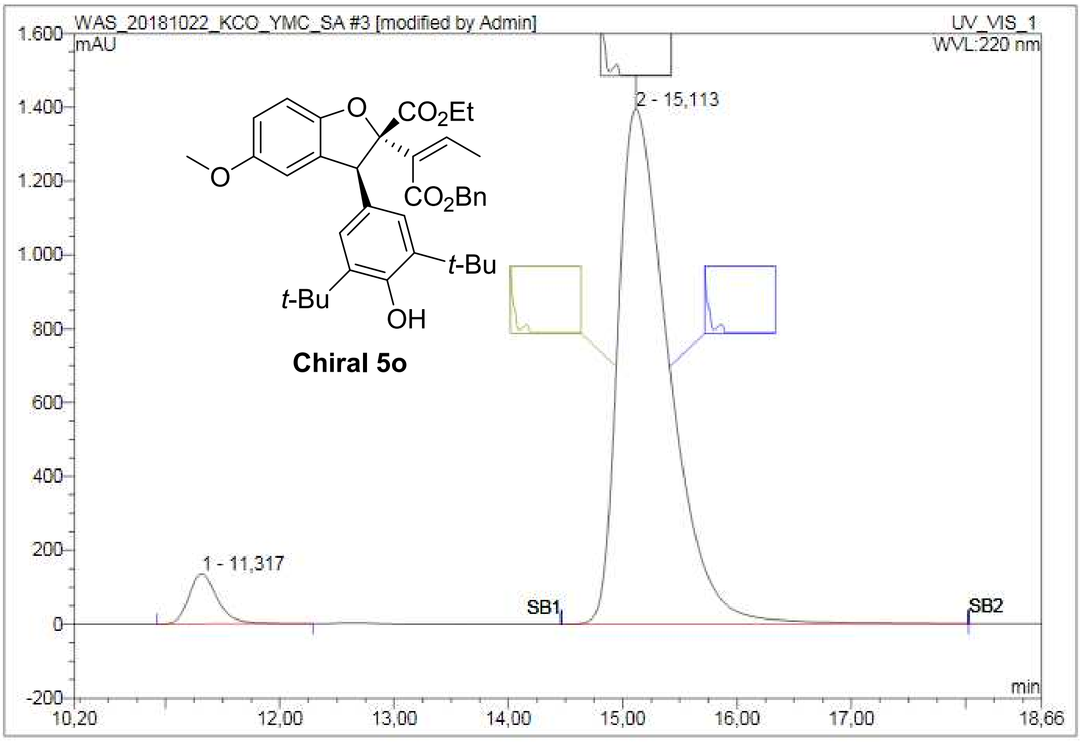

Chiral 5o

No.	Ret.Time min	Peak Name	Height mAU	Area mAU*min	Rel.Area %	Amount	Type
1	11,32	n.a.	135,746	39,363	5,19	n.a.	BMB
2	15,11	n.a.	1393,745	718,832	94,81	n.a.	BMB
**Total:**			1529,491	758,194	100,00	0,000	
